# Systematic review of rodent studies of deep brain stimulation for the treatment of neurological, developmental and neuropsychiatric disorders

**DOI:** 10.1038/s41398-023-02727-5

**Published:** 2024-04-11

**Authors:** Kristina K. Zhang, Rafi Matin, Carolina Gorodetsky, George M. Ibrahim, Flavia Venetucci Gouveia

**Affiliations:** 1 Institute of Medical Science, University of Toronto, Toronto, ON Canada; 2 Program in Neuroscience and Mental Health, The Hospital for Sick Children, Toronto, ON Canada; 3 Division of Neurology, The Hospital for Sick Children, Toronto, ON Canada; 4 Division of Neurosurgery, The Hospital for Sick Children, Toronto, ON Canada

**Keywords:** Neuroscience, Psychiatric disorders

## Abstract

Deep brain stimulation (DBS) modulates local and widespread connectivity in dysfunctional networks. Positive results are observed in several patient populations; however, the precise mechanisms underlying treatment remain unknown. Translational DBS studies aim to answer these questions and provide knowledge for advancing the field. Here, we systematically review the literature on DBS studies involving models of neurological, developmental and neuropsychiatric disorders to provide a synthesis of the current scientific landscape surrounding this topic. A systematic analysis of the literature was performed following PRISMA guidelines. 407 original articles were included. Data extraction focused on study characteristics, including stimulation protocol, behavioural outcomes, and mechanisms of action. The number of articles published increased over the years, including 16 rat models and 13 mouse models of transgenic or healthy animals exposed to external factors to induce symptoms. Most studies targeted telencephalic structures with varying stimulation settings. Positive behavioural outcomes were reported in 85.8% of the included studies. In models of psychiatric and neurodevelopmental disorders, DBS-induced effects were associated with changes in monoamines and neuronal activity along the mesocorticolimbic circuit. For movement disorders, DBS improves symptoms via modulation of the striatal dopaminergic system. In dementia and epilepsy models, changes to cellular and molecular aspects of the hippocampus were shown to underlie symptom improvement. Despite limitations in translating findings from preclinical to clinical settings, rodent studies have contributed substantially to our current knowledge of the pathophysiology of disease and DBS mechanisms. Direct inhibition/excitation of neural activity, whereby DBS modulates pathological oscillatory activity within brain networks, is among the major theories of its mechanism. However, there remain fundamental questions on mechanisms, optimal targets and parameters that need to be better understood to improve this therapy and provide more individualized treatment according to the patient’s predominant symptoms.

## Introduction

The use of deep brain stimulation (DBS) as a treatment for neuropsychiatric disorders and symptoms is among the most important recent advances in clinical neuromodulation. DBS is a neurosurgical procedure that involves the implantation of electrodes into specific brain targets to modulate local and widespread connectivity in dysfunctional networks [1]. To date, several thousands of patients have undergone DBS for various neuropsychiatric conditions [2–6]. Notably, patients with Parkinson’s disease (PD) are among the most common candidates for this treatment option [7]. In select cases, DBS can also induce long-term alleviation of symptoms in patients with dystonia [8], Tourette’s syndrome [9], or epilepsy [10]. In terms of psychiatric conditions, clinical DBS studies have shown promise in relieving symptoms of obsessive-compulsive disorder (OCD) [11, 12], major depressive disorder [13], and substance use disorder (SUD) [14, 15]. Despite encouraging reports, the optimal DBS brain targets and underlying mechanisms that lead to benefits and/or side effects in distinct pathologies remain unclear.

Experimental DBS in animal models plays an essential role in our understanding of the multiscale neurobiological mechanisms of DBS, as well as the development of new technologies. Rodents — in particular, the Norway rat (*Rattus norvegicus*) and the house mouse (*Mus musculus*) — are especially useful for this purpose. Different models can capture core features observed in neuropsychiatric disorders and can be objectively tested in a series of standardized behavioural tests. In addition, the underlying mechanisms of DBS may be explored using healthy rodent strains [16, 17]. In this article, we present a systematic review of preclinical DBS studies to synthesize the literature on the current landscape of DBS in rodent models.

## Methods

A systematic analysis of the international literature was performed in accordance with PRISMA [18] guidelines (See Supplementary Fig. 1 for PRISMA flow diagram). The PubMed MEDLINE (National Library of Medicine) database was used to search articles published between January 2000 and December 2022, using the search terms “deep brain stimulation”, “DBS”, “rodent”, “mouse”, and “rat”. Duplicates and unrelated reports were excluded. The remaining reports were first screened by title and abstract, then selected articles progressed to full-text review based on prespecified inclusion and exclusion criteria (Inclusion: Studies published in English, Original articles with full-text availability, Articles describing studies of DBS in rats or mice, Articles reporting stimulation parameters used; Exclusion: Non-original studies (i.e., conference abstracts, reviews, meta-analyses, commentaries, editorials, and protocols), Articles reporting on clinical population, Articles using other neuromodulatory techniques, Investigations performed in-vitro or using other species) See Supplementary References for full list of included studies. Data extraction was performed by three authors (KZ, RM, FVG) focusing on 5 categories: Bibliographic data, Animal model characteristics (group based on DSM-5 classification [19]; Supplementary Table 1), DBS target and settings, Behavioural outcomes, Mechanisms of action.

## Results

Of the 1674 reports screened, 407 original research articles were included in this review (rat: 356, mouse: 51; Supplementary Fig. 1, Supplementary References). Considering the differences in disease models between species (which may involve distinct cellular and molecular targets), the data extracted from mouse and rat studies are presented separately (see Supplementary Table 2 for the summary of combined data).

### Rodent models of DBS

**Fig. 1:**
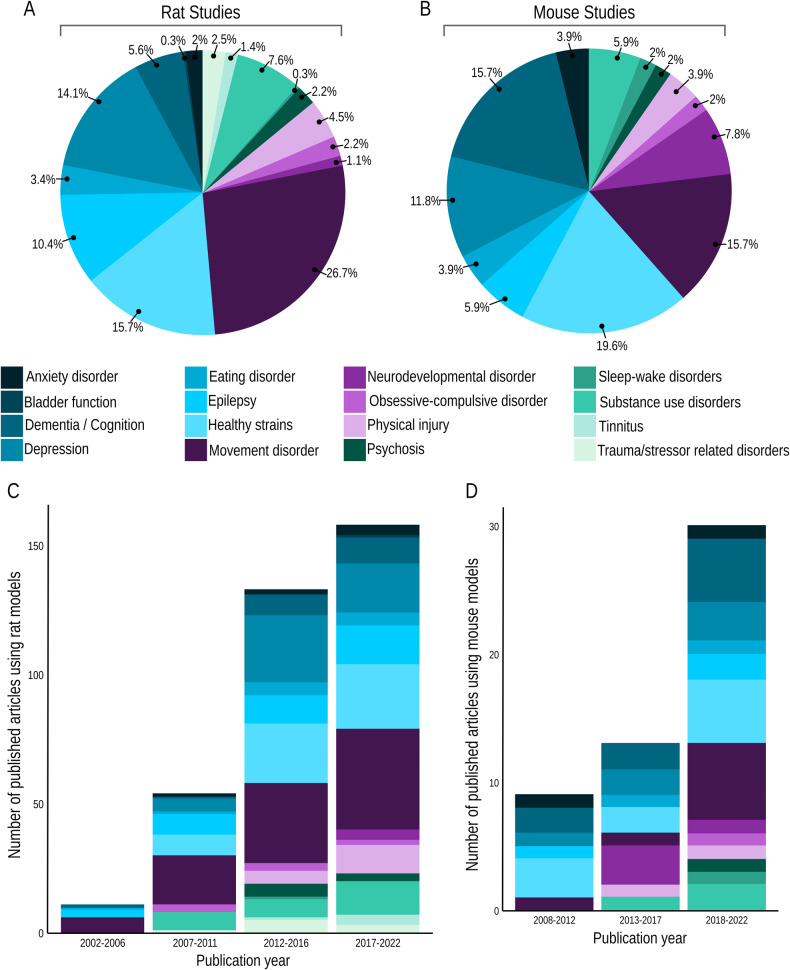
Rodent studies by disease model. Total percentage of rodent studies in each disease model, published after the year 2000, for rats (**A**) and mice (**B**). Number of published articles by period using rat models (**C**) and mouse models (**D**).

Figure 1 shows the proportion of articles published by the disease model and the distribution of these articles over the years. Among rat studies, DBS effects have been investigated in 15 distinct models of disorders and in animals presenting no pathology (i.e., standard healthy strains; Fig. 1A). DBS for movement disorders was the most researched field and included Parkinson’s disease (PD; 24.2%), motor impairments (1.1%), dyskinesia (0.8%), and tremor (0.6%). Depression and epilepsy were the second and third most studied models, followed by SUD (Cocaine 2.8%, Morphine 2.8%, Alcohol 0.8%, Methamphetamine 0.6%, Heroin 0.3%). Models of dementia/cognition focused on cognitive performance (3.8%), Alzheimer’s disease (AD; 1.1%), and dementia (1.1%). Models of physical injury (traumatic brain injury, TBI: 1.4%, pain: 1.1%, spinal cord injury: 1.1%, stroke: 0.6%, ischemia: 0.3%) and eating disorders (obesity: 1.4%, food intake: 0.8%, hedonic feeding: 0.8%, glucose metabolism: 0.3%), occupied smaller portions of the rat DBS landscape. Trauma or stressor-related disorders (i.e., post-traumatic stress disorder, PTSD), obsessive-compulsive disorder, psychosis, anxiety disorders, tinnitus, neurodevelopmental disorders, bladder function (related to neurological disorders), and sleep-wake disorders represented the least studied rat models for DBS.

For mouse studies, DBS effects have been investigated in 12 distinct models of disease and in healthy strains (Fig. 1B). Dementia/cognition (AD: 9.8%, cognitive performance: 3.9%, dementia: 2.0%) and movement disorders (PD: 9.8%, ataxia: 3.9%, tremor: 2.0%) were the most studied, followed by depression models. Neurodevelopmental disorders (Rett syndrome: 5.9%, Autism Spectrum Disorder, ASD: 2.0%), SUD (alcohol: 2.0%, cocaine: 3.9%), epilepsy, anxiety disorders, eating disorder (hedonic feeding: 2.0%, obesity: 2.0%), and physical injury (TBI: 2.0%, stroke: 2.0%) were investigated less frequently. OCD, psychosis, and sleep-wake disorder represented the least studied mouse models for DBS. There has been an upward trend in preclinical DBS publications since the early 2000s (Fig. 1C & D). To our knowledge, no rat DBS study was published between 2000–2002, and no mouse DBS study was published between 2000–2007. In the early stages, these studies focused on PD, epilepsy, or healthy animals; however, the disease models evaluated for DBS effects have substantially diversified over time.

### Model characteristics

**Fig. 2:**
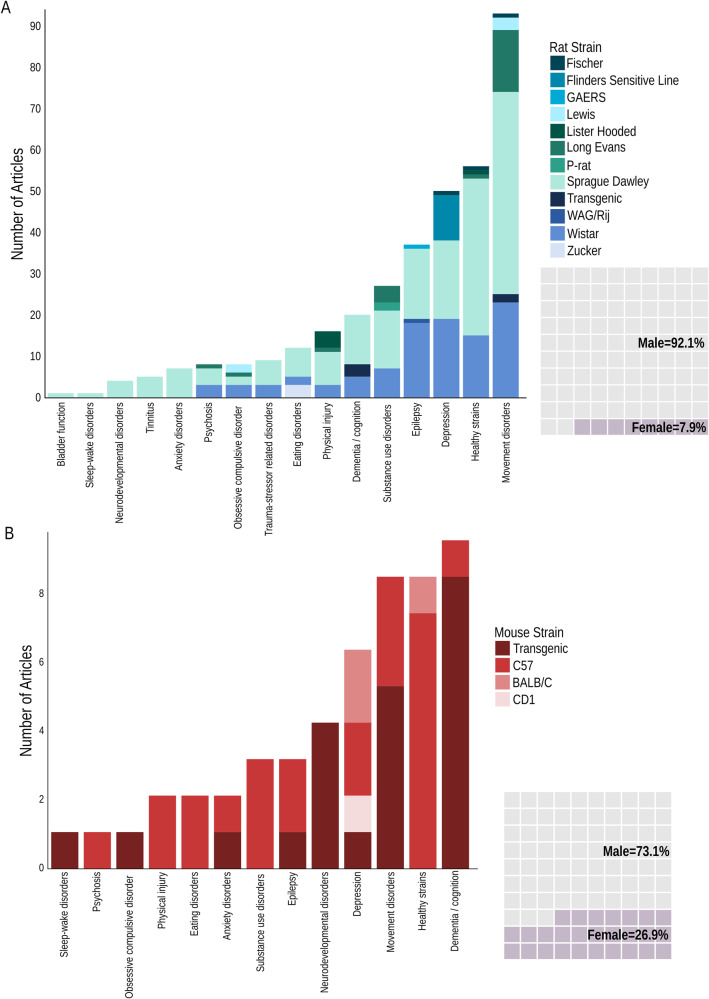
Rodent strain by disease model, and percentage of animals used by sex. **A** Rat studies. **B** Mouse studies. Abbreviations: C57: C57BL/6, CD-1: Cluster of Differentiation 1, GAERS: Genetic Absence Epilepsy Rat, P-rat: alcohol-preferring rat, WAG/Rij: Wistar Albino Glaxo from Rijswijk.

Although Sprague-Dawley (SD; 52.9%) and Wistar rats (30.1%) were the main healthy strains used (Fig. 2A), a handful of studies used Long Evans or Lewis rats to model psychosis [20], OCD [21–23], cocaine [24, 25] or alcohol addiction [26], catalepsy [27–29], PD [30–44], TBI [45, 46], and spinal cord injury [27, 28]. The unilateral 6-hydroxydopamine (6-OHDA) nigrostriatal lesion PD model was the most commonly used ( > 87% of included studies). While neurodevelopmental disorders were modelled in SD rats by exposure to valproic acid [47, 48], apomorphine [49] or antinuclear antibody [50], the Flinders Sensitive Line and Zucker rats were used exclusively to model depression and obesity, respectively. For modelling epilepsy, most studies used SD and Wistar rats receiving chemoconvulsants (e.g., kainic acid, pilocarpine, pentylenetetrazol, or FeCl_3_ solution in one study [51]) or chronic electrical stimulation of the temporal lobes, predominantly the amygdala. Transgenic models of absence seizures (i.e., Generalized Absence Epilepsy in Rats from Strasbourg [52], and Wistar Albino Glaxo/Rijwijk [52, 53]), AD [54, 55] and Huntington’s disease [56] were less frequently used. Conversely, almost half of mouse models (44.2%) were generated using transgenic mice (Fig. 2B), especially for studying neurodevelopmental disorders (i.e., *MECP2* [57–60], *Shank3* [57], or *CDKL5* [61]), OCD (i.e., *Sapap3* [62]) and sleep-wake disorders (i.e., *Tg(HCRT-MJD)1Stak* [63]) which used transgenic animals exclusively. The C57BL/6J mouse (46.2%) was the most common healthy strain used, with animals being exposed to external factors to induce symptoms. The high percentage of studies using transgenic mouse models may help explain the great difference between the rat and mouse literature reporting on using both males and females in the same study (Rat: 1.9%, Mouse: 19.2%). Nevertheless, most studies utilized only male animals for both species (Fig. 2). Supplementary Table 3 describes the validity of rodent models of disease.

### Stimulation parameters

DBS parameters are generally programmed by amplitude (Amperes [A] or Volt [V]), pulse width (seconds), and frequency (Hertz [Hz]). Among the included studies, DBS was predominantly administered at high frequency (130 Hz), short pulse duration (60 µs), and amplitudes of 100 µA or 2.7 V. Although the average parameters used in rat and mouse models fell within similar ranges, the average stimulation parameters were higher in rats. Understanding the pattern of stimulation is fundamental for advancing the preclinical field of research and interpreting the results reported. See Supplementary Table 4 for a detailed description of the stimulation parameters reported. In rats, the highest average amplitude was observed in healthy strains, neurodevelopmental disorders and epilepsy, and the lowest average amplitude was reported in models of OCD and tinnitus. However, studies using healthy strains also showed the largest amplitude range, followed by models of movement disorders and dementia/cognition. For mouse studies, the largest average amplitude was observed in models of OCD and physical injury, and the lowest was reported in models of neurodevelopmental disorders. Notably, a few studies reported stimulation intensities above 1000 µA [64–66], resulting in a large overall range. Most studies used high-frequency stimulation, with 1 kHz being the max for healthy rats and mice. The lowest frequency was observed in rat and mouse models of epilepsy, as well as rat studies of dementia/cognition and movement disorders. On average, studies in rats employed longer pulse widths; however, both species showed the same mode (60 µs).

**Fig. 3:**
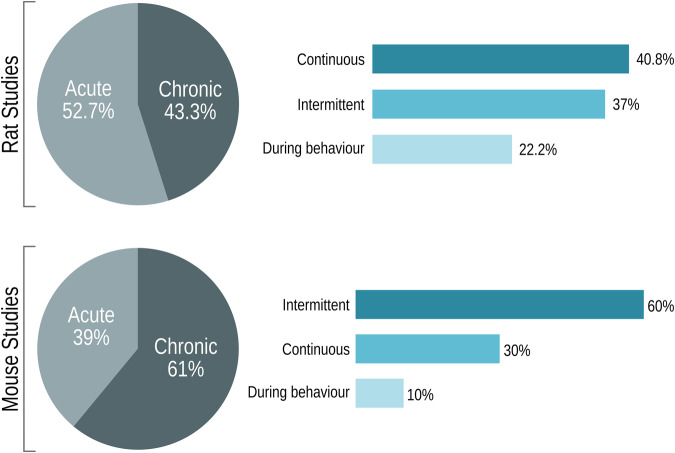
Stimulation settings for rat and mouse studies.

There was large heterogeneity surrounding stimulation duration and administration pattern among the included studies. While acute stimulation (i.e., stimulation ≤ 1 day) was more commonly observed in rat studies, chronic stimulation (i.e., stimulation ≥ 2 days) was more frequently applied in mouse studies (Fig. 3). DBS has also been applied intermittently (series of sessions with DBS-on separated by periods of DBS-off), continuously (DBS-on only), or during behavioural testing (the least explored option in both species). Although rat studies similarly used continuous or intermittent stimulation, mouse studies focused on intermittent stimulation, performed twice as often as continuous stimulation.

### Brain targets

**Fig. 4:**
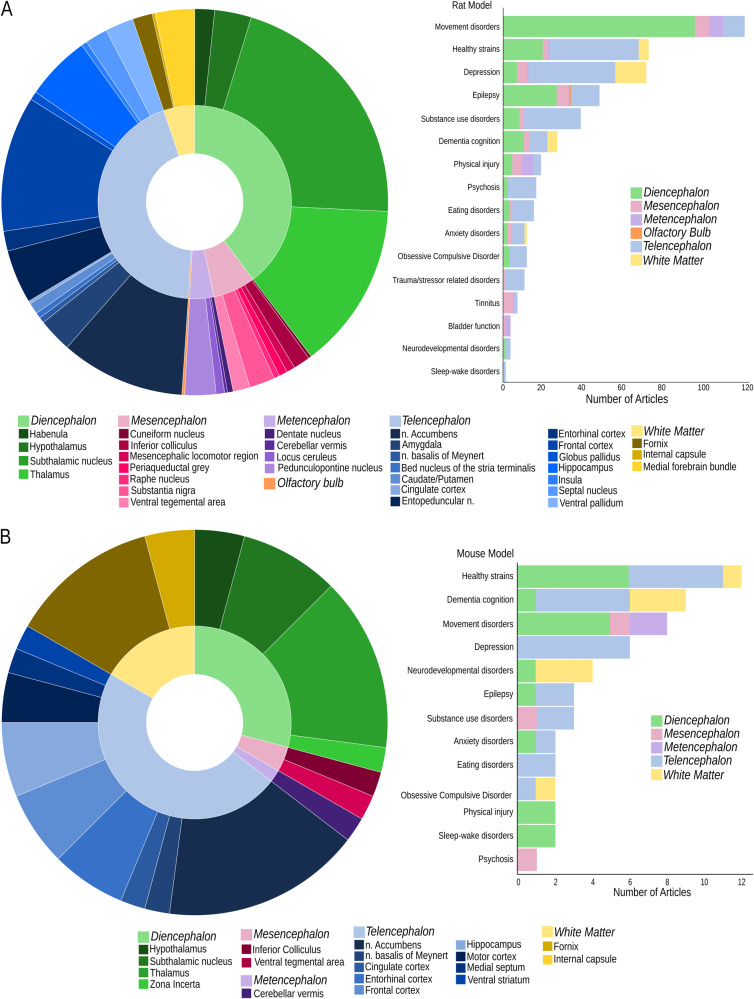
Brain targets for neurostimulation. The proportion of studies targeting diverse brain areas and the number of articles published by disease model colour-coded for the brain target region. **A** Rat studies. **B** Mouse studies.

The majority of studies targeted telencephalic brain areas (rat: 43.7%, mouse: 47.9%) and diencephalic areas (rat: 39.7%, mouse: 29.2%). While white matter structures were more commonly targeted in mouse studies (rat: 5.2%, mouse: 16.7%), mesencephalic areas were more frequently targeted in rat studies (rat: 7.1%, mouse 4.2%). The metencephalon was the target of DBS in 4% of rat studies and 2.1% of mouse studies, and the olfactory bulb was targeted in rats only (0.2%; Fig. 4). Across rat studies, the subthalamic nucleus (STN) was the most common target (22.2%), followed by the thalamus (13.5%), nucleus accumbens (nAcc; 11.7%), and frontal cortex (FC; 11.2%, Fig. 4A). Although the majority of studies targeting the STN investigated models of PD, a few articles have also explored this target in models of SUD [24, 67], depression [68, 69], epilepsy [70, 71], OCD [23], and psychosis [72]. Thalamic targets have been studied in various models, with the anterior nucleus of the thalamus (ANT) being predominantly studied in epilepsy [73–83]. The nAcc was targeted in over half of studies investigating SUD and has also been explored in models of eating disorders, depression, OCD, psychosis, and anxiety disorder. Overall, 17% of the studies using healthy strains targeted the core and shell aspects of the nAcc. FC structures have been extensively targeted in healthy strains [84–90] and in models of depression, SUD [91–93], psychosis [72, 94, 95], cognitive performance [96, 97], anxiety [98], ASD [48], eating disorder [99], and PTSD [100], with most studies applying chronic stimulation, including chronic continuous stimulation for several days [68]. Among mouse studies (Fig. 4B), the thalamus and nAcc were the most commonly targeted brain structures (both 14.8%), followed by the STN (13%) and fornix (10.9%). The central nucleus of the thalamus was the thalamic region most commonly targeted (40%), mainly in studies investigating underlying mechanisms of DBS in healthy strains. Stimulation of the nAcc was investigated in models of depression [101, 102], anxiety disorder [103], eating disorder [104], and SUD [105, 106] however, the number of articles on each model is limited. The STN was predominantly explored in the context of movement disorders [107–109] (71%), and forniceal stimulation has been investigated exclusively in models of neurodevelopmental disorders [58–60] and dementia/cognition [61, 110, 111].

### Mechanisms of action

DBS effects on neurochemistry and electrophysiology were the most commonly investigated mechanisms. These studies focused on neuronal activity (26%), gene/protein expression (25.3%), neurotransmitter levels (23%), neuroinflammation and neuroprotection (15.5%), and neural/synaptic plasticity (6.6%). Electrophysiological studies accounted for 31.7% of published articles (Table 1). In models of depression, anxiety disorder, OCD, and PTSD, the DBS-induced antidepressant- and/or anxiolytic effects were associated with changes in serotonin and dopamine levels, as well as modulation of neuronal activity along the mesocorticolimbic circuit. In DBS for PD and movement disorders, the improvements in motor and non-motor symptoms following treatment have primarily been attributed to modulation of the striatal dopaminergic system through the protection of dopaminergic cells and normalization of dopamine signalling. In models of dementia and epilepsy, DBS attenuates aberrant cellular and molecular changes in the hippocampus, a key structure involved in both memory function and seizure generation. Electrophysiological data were limited in models of dementia and memory, OCD, physical injury, and SUD and were not reported in anxiety disorder, bladder function, and eating disorders. In contrast, the majority of studies using non-pathological strains reported on DBS-induced electrophysiology effects, which were target- and parameter-specific.

**Table 1:** Behavioural, neurochemical, and electrophysiological changes associated with deep brain stimulation based on disease modeled and brain target.

Model	Brain Target	Behaviour	Neurochemical/ Electrophysiological Effects
Anxiety Disorder	Hypothalamus	–Ventromedial hypothalamus HFS induced panic-related behaviours	*Neurochemistry:* –Ventromedial hypothalamus DBS increased local neuronal activity (*c-Fos*)
	RNu	–Anxiolytic- and panicolytic-like effect	*Neurochemistry:* –Increased neuronal activity (*c-Fos*) in medial amygdala, MS, and CC
Dementia/Cognition	Thalamus	–Improved memory deficits	*Neurochemistry:* –Increased neuronal activity (*c-Fos*) in cerebral regions, somatosensory cortex, striatum, and/or hippocampus –Activation of ACC, motor cortex, somatosensory cortex, CPu, hypothalamus, thalamus, and hippocampus, as measured by fMRI –Central thalamic DBS increased expression of DA receptors (i.e. D1R, D2R) and ACh receptor (i.e. ɑ4-nAChR) in striatum and hippocampus –Increased synaptic plasticity (dendritic spine density) in hippocampus and mPFC *Electrophysiology:* –Central thalamic DBS increased theta and alpha LFP oscillations in thalamic central lateral nuclei and striatum
	EC	–Improved memory deficits	*Neurochemistry:* –Increased neurogenesis (BrdU/NeuN staining) in DG –Decreased beta-amyloid plaque deposition in hippocampus and cortex
	Fornix	–Improved learning and memory deficits	*Neurochemistry* –Increased neuronal activity (*c-Fos*) in hippocampal CA1 and CA3 –Decreased astrogliosis and microglial activation, lowered neuronal loss in cortex and hippocampus –Reduced beta-amyloid deposition in hippocampus and cortex –Increased extracellular ACh and/or glutamate in hippocampus –Increased hippocampal glucose metabolism
	NBM	–Improved memory deficits	–*Neurochemistry:* –Induced neuroprotective effects—increased neuron survival, reduced apoptotic cells in hippocampus and cortex, mitigated oxidative stress and regulated ACh –Increased neuronal activity (*c-Fos*) in perirhinal cortex, CA1, CA3, DG –Increased cholinergic fibre length in DG
	Hippocampus	–Restored memory loss in object location task	*Neurochemistry:* –Increased neuronal activity (*c-Fos*) in anterior cingulate gyrus
	Medial septum	–Restored spatial memory	*Neurochemistry:* –Increased hippocampal cholinergic activity and neurogenesis
	Frontal cortex	–OFC DBS induced cognitive impairment –PL DBS improved spatial learning and memory in Morris water maze	*Neurochemistry:* –PL-DBS altered the expression of glutamate and neurogenesis-related genes, including G protein-coupled receptor pathways
	VS	–Decreased fear extinction and improved extinction memory	*Neurochemistry:* –Increased neuronal activity (*c-Fos*) and BDNF in PL and IL cortices
Depression	Frontal cortex	–Antidepressant-, anxiolytic-, and/or antianhedonic-like effects	*Neurochemistry:* –Depleting 5-HT transmission prevents some DBS-induced antidepressant-like effects –Chronic-DBS induced long-term elevation of 5-HT levels –Increased hippocampal 5-HT release –DBS effects are independent of SERT –Increased expression or activity of BDNF, Akt, and mTOR in hippocampus –Increased neuronal activity and/or plasticity (*c-Fos, zif268*) in PFC and structures along limbic circuit –Increased NE release in PFC –Adenosine A1 and glutamatergic AMPA receptor antagonists block DBS-induced antidepressant-like effects *Electrophysiology:* –Normalized beta, theta, and high gamma band activity at VTA, vmPFC, and/or hippocampus –Enhanced DR 5-HT neuronal excitability –Increased coherence in beta and gamma bands between vmPFC and hippocampus
	nAcc	–Antidepressant- and/or anxiolytic-like effects –Shell DBS worsened performance in learned helplessness paradigm	*Neurochemistry:* –Acute core DBS increased 5-HT in mPFC –Acute core DBS increased NE in OFC –Acute core DBS increased DA in mPFC and OFC –Chronic shell DBS decreased DA and tyrosine hydroxylase in mPFC –Shell DBS increased dendrite length in PFC pyramidal neurons –Chronic core DBS led to hippocampal neurogenesis
	MFB	–Antidepressant- and anxiolytic-like effects –Improved memory function	*Neurochemistry:* –Increased 5-HT and NE levels in PFC –Increased DA levels in nAcc, PFC –Enhanced D2R and DAT expression in hippocampus and PFC –Increased neuronal activity and/or plasticity (*c-Fos, zif268*) in target regions of mesocorticolimbic system *Electrophysiology:* –Increased gamma band oscillations in PFC of FSL rats
	LHb	–Antidepressant- and anxiolytic-like effects	*Neurochemistry:* –Increased 5-HT, DA, NE in stressed animals *Electrophysiology:* –Potentiated EPSCs in hippocampus
	VTA	–Long-term antidepressant-like effects	*Neurochemistry:* –Increased BDNF expression in hippocampus *Electrophysiology:* –Acute LFS normalized intra-VTA LFP activity and increased VTA LFP synchronicity
	EPN	–Reduced vacuous chewing movements	*Neurochemistry:* –Decreased neuronal activity and plasticity (*zif268*) in motor cortex, thalamus, all basal ganglia structures and raphe –Decreased hippocampal BDNF and trkB expression
	STN	–Increased depression-like behaviours –Reduced vacuous chewing movements	*Neurochemistry:* –Decreased neuronal activity and plasticity (*zif268*) in motor cortex and thalamus –Increased neuronal activity and plasticity (*zif268*) in GP and SN
	CBv	–Antidepressant-, anxiolytic-, and anti anhedonia-like effects	*Neurochemistry:* –DBS effects on neural activity blocked by 5-HT_1A_ antagonist *Electrophysiology:* –Enhanced neural firing activity of dorsal raphe 5-HT neurons but not mPFC neurons
Eating Disorder	nAcc	–Anti-binge eating-like effects –Dissociation of effects depending on subregion targeted — core DBS decreased high fat/ high sucrose intake when DBS applied *before* binge, shell DBS decreased intake *during* binge –Mixed effects on body weight — weight gain in female SD rats, weight loss in male C57BL/6 mice	*Neurochemistry:* –Shell DBS increased glucagon and glucose concentrations in plasma, associated with increased neuronal activity (*c-Fos*) in LHA –Increased DA levels and D2R gene expression in model of anorexia nervosa –D2R antagonist attenuated DBS effects, D1R antagonist did not alter DBS effects
	LHA	–Decreased body weight and food intake	*Neurochemistry:* –Decreased density of puncta-expressing PSA-NCAM in hippocampus and EC –Decreased density of punta-expressing VGAT in EC –Increased metabolism in mammillary body, hippocampus, amygdala, decreased metabolism in thalamus, caudate, temporal cortex, cerebellum
Epilepsy	ANT	–Decreased frequency and severity of focal and generalized seizures –Increased latency of seizure onset –Delayed the progression of kindling –Increased duration of REM sleep	*Neurochemistry:* –Neuroprotective effects in hippocampus—increased neurogenesis, decreased neuron cell loss, increased inflammatory cytokine levels –Increased GABAergic interneurons in hippocampus –Decreased neuronal activity (*c-Fos*) in hippocampus –Increased adenosine and decreased ADK expression in hippocampus –Decreased mossy fibre sprouting in CA3 and DG –Increased levels of 5-HT metabolite in thalamus –Altered expression of genes involved with calcium, glutamate, and NOD-like receptor signalling *Electrophysiology:* –Decreased cortical theta and increased cortical gamma oscillations –Suppressed delta oscillations during nREM sleep
	Hippocampus	–Decreased frequency and severity of electrographic and behavioural seizures –Increased latency and threshold of evoked afterdischarges	*Neurochemistry:* –Increased GABA-A receptor expression in hippocampus
	BLA	–Decreased frequency and duration of seizures	*Neurochemistry:* –No effect on density of PV and NPY expressing hippocampal interneurons Electrophysiology: –Increased hippocampal theta power –Reduced pathologically increased phase-amplitude coupling in hippocampus
	SN	–Decreased frequency of seizures	*Neurochemistry:* –HFS decreased neuronal activity (*c-Fos*) in SN
	VP	–Decreased frequency and duration of seizures –Increased latency of seizure onset	*Electrophysiology:* –Increased GABAergic neuronal firing activity in VP
	Posterior hypothalamus (TMN, PFN)	–TMN- and PFN-DBS decrease seizure severity –TMN-DBS decreased seizure duration and increased latency of seizure onset	*Neurochemistry:* –Increased histamine release in frontal cortex *Electrophysiology:* –Desynchronization of cortical EEG
	MS	–Reduced frequency of spontaneous seizures –Improved memory performance	*Electrophysiology:* –Increased hippocampal theta power
Healthy	Thalamus	–Mixed effects on motor activity—higher current amplitude increased motor activity	*Neurochemistry:* –Decreased activated local microglia –Increased hippocampal neurogenesis –ANT DBS increased tyrosine hydroxylase immunoreactivity in VTA –ANT DBS increased neuronal activity (*c-Fos*) in structures along mesocorticolimbic circuit –Glutamate concentration increased linearly with increasing DBS duration, frequency, intensity, and pulse width *Electrophysiology:* –Increased spectral power of slow waves in cortical EEG -–Oscillatory activity in low-frequency band in cortex and GP associated with tremor
	Hippocampus	–Not described	*Neurochemistry:* –Increased neuronal activity (*c-Fos*) in hippocampus –Evoked BOLD response in hippocampus and other mesolimbic structures, dependent on DBS intensity –Decreased glucose metabolism in hippocampus and limbic structures, measure by FDG-PET *Electrophysiology:* –Low DBS amplitudes reduced EPSCs, more long-lasting effects with longer DBS duration –LFS decreased firing rates of pyramidal cells –HFS with different frequencies did not affect neuronal firing rate –HFS with smaller pulse width generated more randomness in neuronal firing time –HFS extended duration of axonal refractory period
	nAcc	–Not described	*Neurochemistry:* –Increased DA in mPFC and OFC, 5-HT in mPFC, and NE in OFC –HFS increased GABA levels *Electrophysiology:* –Decreased PFC neuronal firing, selectively modulated afferent input to PFC, and potentiated OFC oscillatory activity –Decreased alpha and increased gamma band coherence in nAcc
	Frontal cortex	–Not described	*Neurochemistry:* –Increased 5-HT levels and SERT expression –Activated widespread networks and brain regions connected with IL cortex, as measured by fMRI –Increased hippocampal neurogenesis –Mixed effects on metabolic activity—mPFC DBS increased metabolism in striatum, amygdala, and PL cortex but reduced metabolism in cerebellum, brainstem, and PAG
	EC	–Enhanced memory performance	*Neurochemistry:* –Increased hippocampal neurogenesis and expression of insulin receptor proteins –Increased functional connectivity among PFC, hippocampus, and EC *Electrophysiology:* –Increased power spectra in PFC- and hippocampal-related networks
	EPN	–Not described	*Neurochemistry:* –HFS decreased expression of GDNF-family receptor isoforms –Frequency-dependent modulation of functional connectivity *Electrophysiology:* –Altered spontaneous and stimulus-induced LFP oscillations along motor cortical-basal-ganglia-thalamic circuit — reduced beta and enhanced gamma synchronization
	GPe	-–Not described	*Neurochemistry:* –Decreased cerebral blood volume in striatum
	MS	–Reduces response to pressure stimuli	*Neurochemistry:* –Increased neuronal activity (*c-Fos, EGR1, NPAS4*), neurotrophins, and inflammatory cytokines in ventral hippocampus
	Fornix	–Not described	*Neurochemistry:* –Increased neuronal activity (*c-Fos*) –Mixed effects on BDNF expression –Decreased density of synaptophysin immunoreactive presynaptic boutons in CA1 and CA3
	PAG	–Nearly completely inhibited reflexive isovolumetric bladder contractions, augmented in LC DBS	*Neurochemistry:* –Decreased neuronal activity (*c-Fos*) and parvalbumin co-localization in hippocampus
	MFB	–Not described	*Neurochemistry:* –Ambiently increased extracellular DA concentration in striatum
	LCN	–Not described	*Electrophysiology:* –Increased cortical excitability
	SN	–Not described	*Electrophysiology:* –Blocked excitatory influence of 5-HT_1A_ receptor activation on AMPAR-mediated EPSCs in ventral hippocampus
Movement Disorder	STN	–Improved dysfunctional motor behaviours in PD models –Decreased tremor and dyskinesia like behaviour –Increased depressive and anhedonia-like behaviour	*Neurochemistry:* –Induced neuroprotective effects—increased DA neuron survival in the SN –Increased BDNF in the nigrostriatal system and primary motor cortex –Induced anti-inflammatory and anti-apoptotic effects –Reduced neuronal activity (*c-Fos*) in raphe nucleus and decreased extracellular 5-HT in striatum, PFC, and hippocampus –Increased striatal DA; mixed effects on DA metabolites, with both increased & decreased levels reported –Altered glutamate and GABA transmission in striatum –DBS-induced effects blocked with NMDA receptor antagonist *Electrophysiology:* –Reduced beta oscillations and abnormal oscillations (i.e. LFOs, HVSs) in cortical and basal ganglia networks –Increased alpha oscillations in STN, mPFC, and motor cortex –Reduced STN firing rate –Induced complex effects on the neuronal activity of basal ganglia network structures –Recovered functional output of motor cortex
	EPN	–Improved dysfunctional motor behaviours in PD models—mixed reports on extent of improvement –Increased impulsivity and anhedonia-like behaviour –Decreased tardive dyskinesia-like behaviours	*Neurochemistry:* –Increased extracellular glutamate in striatum –No effect on 5-HT, BDNF, and neuroprotective factors *Electrophysiology:* –Normalized EPN firing rate and restored LFP power
	Thalamus	–Anti-akinetic effects –Mixed effects on memory—improved memory in animals with severe cognitive impairment, impaired memory in animals with normal or mild cognitive impairment	*Neurochemistry:* –Normalized expression marker of striatal neurons of the indirect pathway –Normalized neuronal activity in GP, as measured by cytochrome oxidase subunit 1 expression *Electrophysiology:* –VA/VL DBS increased firing of VL neurons –VL DBS decreasing firing of motor cortex neurons and increased thalamocortical theta and HFO power –Reduced cortical beta and gamma oscillations
	Inferior colliculus	–Improved dysfunctional motor behaviours in models of catalepsy or PD –Anxiolytic-like effects	*Neurochemistry:* –Activated PAG, SN, PPTg, superior colliculus, and cuneiform nucleus Electrophysiology: –Increased neuronal firing at inferior capsule
	PPTg	–Improved dysfunctional motor behaviours in PD model –Anterior PPTg DBS worsened motor behaviours	*Neurochemistry:* –Increased local neuronal activity (*c-Fos*) Electrophysiology: –Reduced STN neuronal firing and beta oscillations
	Cerebellum	–Improved memory and motor recovery in ataxia model	*Neurochemistry:* –Increased anti-inflammatory cytokine levels
	Hypothalamus	–Anti-cataleptic effects –Restored motor behaviours in cataleptic animals –Anti-akinetic effects in PD model	*Electrophysiology:* –Restored hippocampal-striatal EEG synchrony
	SN	–Improved dysfunctional motor behaviours –Induced anhedonia and decreased motivation	*Neurochemistry:* –Decreased 5-HT levels in mPFC *Electrophysiology:* –Decreased SN neural activity and increased neural activity at the ventromedial thalamus –Decreased SN beta oscillations
Neurodevelopmental Disorder	Fornix	–Rescued memory deficits (i.e. contextual fear memory and spatial memory)	*Neurochemistry:* –Normalized expression of up to 25% of genes altered in mouse models of intellectual disabilities *Electrophysiology:* –DBS rescued long term potentiation in PP-DG pathway in *CDKL5*^*−/−*^ mice –Restored feedforward inhibition in DG of *CDKL5*^*−/−*^ mice –Chronic DBS normalized synchrony between CA1 pyramidal neurons and restored spontaneous EPSC frequency and amplitude in *Mecp2*^*−/−*^ mice
	Thalamus	–Decreased excessive self-grooming behaviour in *Shank3B*^*−/−*^ and *Mecp2*^*−/−*^ mice –Mixed effects on exploration and sociability	*Neurochemistry:* –Decreased D2R expression in striatum *Electrophysiology:* –Activated cortical areas, limbic areas, and dorsal striatum –Restored functional connectivity in corticostriatal and corticolimbic circuits –Reduced theta band activity between centromedian-Pf complex LFPs and SMCtx EcoG in striatal regions of Tourette’s rat model
	PFC	–Improved sociability, anxiety-like behaviour, and hyperlocomotion	*Neurochemistry:* –5-HT_1A_ antagonist blocked DBS effects –Decreased expression of NR2B subunit of NMDA receptors and β3 subunit of GABA receptors in PFC
	EPN	–Decreased tic behaviour score in rat model of Tourette’s syndrome	*Neurochemistry:* –Decreased DA concentration and DAT expression in striatum of Tourette’s rat model
Obsessive-compulsive Disorder	nAcc	–Mixed behavioural effects dependent on sub-region targeted—core DBS decreased impulsivity and perseverative-like behaviour, shell DBS increased impulsivity but decreased perseverative behaviour	*Neurochemistry:* –Shell DBS increased DA and 5-HT in nAcc *Electrophysiology:* –Core HFS reduced OFC neuronal firing –Core HFS enhanced OFC spontaneous LFP oscillatory activity in slow (0.5–4 Hz) frequency band
	Thalamus	–MD thalamus DBS increased impulsivity –Pf thalamus DBS alleviated PPI –STN DBS reduced maladaptive decision making in a rat gambling task	*Neurochemistry:* –MD thalamus DBS decreased neuronal activity (*c-Fos*) in all cerebellar nuclei and PFC
	VS	–DBS during extinction training reduced fear expression and strengthened extinction memory—most effective when targeting dorsomedial VS –Eliminated persistent avoidance in rats treated with therapy comparable to exposure with response prevention	*Neurochemistry:* –Dorsomedial VS DBS increased number of pERK-labelled neurons in PL and IL cortices, OFC, and amygdala
	IC	–Decreased excessive self-grooming in *Sapap3*^*−/−*^ mice	*Neurochemistry:* –Increased neuronal activity (*c-Fos*) locally and in PFC
Physical injury	Mesencephalic locomotor region	–Improved motor function in rat models of SCI	Neurochemistry: –Increased BDNF expression and TrkB-Akt-mTOR pathway signalling in spinal cord tissue –Enhanced synaptic plasticity (i.e. SVP38 and PSD95 expression)
	LCN	–Enhanced motor recovery in rat models of TBI	*Neurochemistry:* –Increased expression of excitability-related genes –Suppressed expression of pro-inflammatory genes –Suppressed apoptosis and activation of microglia and astrocytes at perilesional site
	LHA	–Restores consciousness in comatose animals following TBI	*Neurochemistry:* –Increased orexin receptor type 1 expression in LHA –Increased noradrenergic signalling (a1-AR expression) and decreased GABAergic signalling (GABA-B receptor expression) in PFC *Electrophysiology:* –Reduced delta oscillations in LHA
	MS, hippocampus	–Improved cognitive function (object exploration and spatial learning) in TBI rat model	*Electrophysiology:* –Increased hippocampal theta oscillations
	Posterior insula	–Decreased mechanical and cold allodynia in rat models of neuropathic pain	*Neurochemistry:* –DBS-induced effects blocked with NMDA receptor antagonist
Psychosis	Hippocampus	–Antipsychotic-like effects –Restored cognitive deficits	*Electrophysiology:* –Normalized DA neural activity in VTA
	MS	–Antipsychotic-like effects	*Electrophysiology:* –Reduced hippocampal gamma oscillations
	VTA	–Antipsychotic-like effects	*Electrophysiology:* –Increased neural activity of GABA and DA neurons in VTA
	mPFC	–Decreased cognitive deficits	*Neurochemistry:* –Restored normal transmission of DA and 5-HT –Altered metabolic activity in parietal cortex, striatum, ventral hippocampus, nAcc, and brainstem
	Thalamus	–Not described	*Electrophysiology:* –Alleviated aberrant thalamic oscillatory activity—reduced number, duration, and amplitude of SWDs –Modified power spectra and coherence in thalamo-cortical networks
Sleep-wake Disorders	LHA, ZI	–Improved sleep-wake consolidation, and ameliorated cataplexy-like behaviour	*Neurochemistry:* –Increased neuronal activity (*c-Fos*) in wake-active nuclei (i.e., within basal forebrain, hypothalamus, thalamus, ventral midbrain)
	GPe	–Increased REM and nREM sleep	*Electrophysiology:* –DBS-induced EEG power spectrum similar to baseline sleep
Substance abuse/addictive Disorders	nAcc	–Decreased consumption, preference, and/or motivation for taking addictive substances –No effect on consumption or preference for water or natural rewards –Mixed behavioural effects depending on sub-region of nAcc targeted	*Neurochemistry:* –Increased GABA and reduced glutamate in mesocorticolimbic region –Activates inhibitory GABA interneurons in afferent structures –Increased GluR1 and GluR2 in amygdala and nAcc –Acute DBS increased local DA levels –Shell DBS enhanced neuronal activity (*c-Fos*) in nAcc and IL-PFC –Core DBS increased expression of pCREB and ΔFosB in nAcc *Electrophysiology:* –Modulate dysfunctional neuronal activity between OFC and thalamocortical circuit –Activation of mPFC and CPu, as measured by fMRI
	VS	–Dorsal VS DBS had no effect on drug reinstatement –Dorsal VS HFS impaired extinction training and memory, LFS strengthened extinction memory	*Neurochemistry:* –Increased neuronal activity (*c-Fos*) in IL and PFC –LFS increased neuronal activity (*c-Fos*) in amygdala
	VTA	–Not described	*Neurochemistry:* –Rapidly increased neuronal Ca^2+^ in mPFC followed by plateau for 5 Hz DBS and immediate decay for 50 Hz DBS
	STN	–30 Hz DBS reduced cocaine seeking	*Electrophysiology:* –8 Hz DBS increased alpha/theta oscillatory activity in STN
	Anterior insula	–HFS decreased morphine preference but relapse occurred 10 days after DBS cessation	*Neurochemistry:* –Normalized expression of 8 morphine-regulated proteins in anterior insula
Tinnitus	Medial geniculate body	–Alleviated tinnitus-like behaviour	*Neurochemistry:* –Enhanced neuronal activity (*c-Fos*) in TRN *Electrophysiology:* –Desynchronized thalamocortical oscillations
	Caudate nucleus	–Alleviated tinnitus-like behaviour	*Electrophysiology:* –Bursting activity reduced in CPu
Trauma/Stress related Disorders	Amygdala	–Anxiolytic-like effects	*Neurochemistry:* –Increased serum corticosterone levels –Decreased neuronal activity (*c-Fos*) in amygdala *Electrophysiology:* –Decreased EPSCs in cortico-amygdala pathway
	IL cortex	–Anxiolytic-like effects	*Electrophysiology:* –Reduced neuronal firing of BLA cells

*5-HT* serotonin, *ɑ4-nAChR* ɑ4-nicotinic acetylcholine receptor, *ACC* anterior cingulate cortex, *ACh* acetylcholine, *ADK* adenosine kinase, *Akt* protein kinase B, *AMPA* α-amino-3-hydroxy-5-methyl-4-isoxazolepropionic acid, *ANT* anterior nucleus of thalamus, *BDNF* brain derived neurotrophic factor, *BLA* basolateral amygdala, *BOLD* blood-oxygen level dependent, *CBv* cerebellar vermis, *CC* cingulate cortex, *CPu* caudate putamen, *D1R/D2R* dopamine 1/2 receptor, *DA* dopamine, *DAT* dopamine transporter, *DBS* deep brain stimulation, *DG* dentate gyrus, *DR* dorsal raphe, *EC* entorhinal cortex, *EcoG* electrocorticography, *EEG* electroencephalogram, *EPN* entopeduncular nucleus, *EPSC* excitatory postsynaptic currents, *FDG-PET* fluorodeoxyglucose positron emission tomography, *fMRI* functional magnetic resonance imaging, *FSL* Flinder’s Sensitive Line, *GABA* γ-Aminobutyric acid, *GDNF* glial cell-line derived neurotrophic factor, *GluR1/2* glutamate receptor 1/2, *GP* globus pallidum, *GPe* external globus pallidus, *GPi* globus pallidus internus, *HFO* high frequency oscillation, *HFS* high frequency stimulation, *HVS* high-voltage spike, *IC* internal capsule, *IL* infralimbic, *LCN* lateral cerebellar nucleus, *LFO* low frequency oscillation, *LFP* local field potential, *LFS* low frequency stimulation, *LHA* lateral hypothalamus, *MD* mediodorsal, *MFB* medial forebrain bundle, *mPFC* medial prefrontal cortex, *MS* medial septum, *mTOR* mammalian target of rapamycin, *nAcc* nucleus accumbens, *NE* norepinephrine, *NMDA* *N*-methyl-d-aspartate, *NOD* Nucleotide-binding oligomerization domain, *NOR* novel object recognition, *NPY* neuropeptide Y, *nREM* non-rapid eye movement, *OFC* orbitofrontal cortex, *PAG* periaqueductal grey, *pCREB* phosphorylated cAMP response element-binding protein, *PD* Parkinson’s disease, *PENK* proenkephalin-A, *pERK* phosphorylated extracellular signal-regulated kinase, *Pf* parafascicular nucleus, *PFC* prefrontal cortex, *PFN* perifornical area, *PL* prelimbic, *PP* perforant path, *PPI* prepulse inhibition, *PPTg* pedunculopontine tegmental nucleus, *PSA-NCAM* polysialylated form of the neural cell adhesion molecule, *PSD95* postsynaptic density 95, *PV* parvalbumin, *REM* rapid eye movement, *RNu* raphe nucleus, *SCI* spinal cord injury, *SD* Sprague-Dawley, *SERT* serotonin transporter, *SMCtx* sensorimotor cortex, *SN* substantia nigra, *STN* subthalamic nucleus, *SVP38* synaptophysin antibody, *SWD* spike-wave discharge, *TBI* traumatic brain injury, *TMN* tuberomammillary nucleus, *TrkB* tropomyosin receptor kinase B, *TRN* thalamic reticular nucleus, *VA/VL* ventral anterior/ventral lateral, *VGAT* vesicular GABA transporter, *vmPFC* ventromedial prefrontal cortex, *VP* ventral pallidum, *VS* ventral striatum, *VTA* ventral tegmental area, *ZI* zona incerta.

### Behavioural outcomes

To investigate if DBS treatment would improve symptoms or behaviours, a large portion of the studies included behavioural tests performed either at the end or during treatment (Tables 1–2, Supplementary Table 5). While 73% of rat studies employed behavioural tests, with positive outcomes reported in 83.4%, 81.4% of mouse research investigated behavioural outcomes, reporting improvement in 88.6% of the studies. Although some behavioural tests are more frequently used in one species over the other, several tests are established and commonly performed in both rats and mice. The open-field test was widely used to assess locomotor activity and/or anxiety-like behaviour in several disease models. The Morris Water Maze was used to investigate spatial memory in models of dementia, the Forced Swimming Test was used for assessing immobility (as a measure of behaviour despair) in models of depression, and the Drug Self-Administration paradigm was used in substance use disorder models. Moreover, quantification of food intake was used in models of eating disorders for both species, as well as recording seizure frequency in models of epilepsy.

**Table 2:** Behavioural outcomes of DBS in rat and mouse models of neuropsychiatric disorders.

Model	Number of articles assessing behaviour	Reported positive behavioural outcomes	Tests most commonly use
***Rat Studies***			
Anxiety Disorder	7	7 (100%)	EPM, OFT
Dementia/Cognition	17	14 (82%)	MWM, ORT
Depression	43	36 (84%)	FST, SPT
Eating Disorder	6	5 (83%)	FI
Epilepsy	11	9 (82%)	SF
Movement Disorder	68	58 (85%)	CT, OFT
Neurodevelopmental Disorder	4	4 (100%)	3CST
Obsessive-compulsive Disorder	8	6 (75%)	RTT
Physical injury	12	11 (92%)	PMT, OFT
Psychosis	5	4 (80%)	PPI
Substance abuse/addictive Disorders	27	25 (93%)	CPP, DSA
Tinnitus	2	2 (100%)	ASR
Trauma/Stress related Disorders	8	5 (63%)	EPM, FC
*Overall*	*218*	*186 (85%)*	*--*
***Mouse Studies***			
Anxiety Disorder	2	2 (100%)	FC
Dementia / Cognition	8	7 (87%)	MWM
Depression	6	6(100%)	FST, TST
Eating Disorder	2	1 (50%)	FI
Epilepsy	3	3 (100%)	SF
Movement Disorder	7	6 (86%)	ART, OFT
Neurodevelopmental Disorder	2	2 (100%)	3CST
Obsessive-compulsive Disorder	1	1 (100%)	SGT, OFT
Physical injury	1	1 (100%)	SMA
Psychosis	1	1 (100%)	OFT
Substance abuse/addictive Disorders	2	1 (50%)	DSA, OFT
*Overall*	*35*	*31 (89%)*	*–*

*3CST* Three Chamber Sociability Test, *ART* Accelerating Rotarod Test, *ASR* Acoustic Startle Response, *CPP* Conditioned Place Preference, *CT* Cylinder Test, *DBS* Deep Brain Stimulation, *DSA* Drug Self-Administration, *EPM* Elevated Plus Maze, *FC* Fear Conditioning, *FI* Food Intake, *FST* Forced Swimming Test, *MWM* Morris Water Maze, *N/A* not applicable, *OFT* Open Field Test, *ORT* Object Recognition Test, *PMT* Pasta Matrix Test, *PPI* Prepulse Inhibition Test, *RTT* Reaction Time Task, *SF* Seizure Frequency, *SGT* Self Grooming Test, *SMA* Spontaneous Motor Activity, *SPT* Sucrose Preference Test, *TST* Tail Suspension Test.

### Adverse events

Only a few studies reported adverse effects from DBS treatment (rat: *n* = 24, mouse: *n* = 1). The most commonly reported side effects were brain injury at the insertion site (i.e., hemorrhage, lesions, inflammation) [70, 112–116] and seizures (onset or worsening) [117–122] when very high frequencies or amplitudes were used [122]. Following DBS, two articles observed depressive-like behaviours in models of movement disorders [69, 109], three articles reported increased impulsivity [21, 22, 123], and two reported tremors [20, 124]. Few studies observed side effects of anxiety-like behaviour [125], mania [126], unexpected weight change [127], fragmented sleep-wake cycles [53], impaired memory [128], and kindling [55].

## Discussion

### Rodent models of DBS

The strength of rodent models for DBS research is rooted in dimensions of face (model’s ability to reflect clinical symptoms), construct (similar disease etiology between human condition and preclinical models), and predictive (response to treatments seen in clinical populations) validity. Most rodent models used in DBS studies demonstrate high face and construct validity, being valuable for analyzing the pathophysiology and behavioural effects of treatment, thus facilitating the development and optimization of therapies [129–131]. Transgenic models of neurological and psychiatric disorders, as well as healthy strains, have been extensively used to provide insights into the intrinsic mechanisms of DBS, treatment safety, and potential adverse effects of stimulation, significantly contributing to advancing the current knowledge and showing great potential for developing targeted therapies [129–131]. These models, however, do not capture all features of human disease but mimic specific relevant symptoms [129–131]. The unilateral 6-OHDA model is the most common model of PD and involves the degeneration of nigrostriatal dopaminergic neurons [132, 133] leading to major motor dysfunctions (e.g., akinesia, tremor) and basal ganglia neuroplasticity similar to what is observed in patients [134]. Although these features support the strong face validity of the model, the behavioural and cellular effects produced in this model depend on the site of drug injection [133], and the resulting changes in glutamate release and beta band frequency in response to DBS are different from those observed in humans [135]. Transgenic models of PD are a good alternative, as they present multiple and more complex symptoms, such as observed in patients [107–109, 136, 137]. Neurodegenerative diseases of aging, such as AD, have also been studied in rodent models either using aged animals [55], intracranial injection of chemicals [138–142] or transgenic models with mutations in AD-related genes [66, 111, 143–146]. These models exhibit clinically relevant features, including the development of β-amyloid plaques and Tau protein aggregates and cognitive decline, which are excellent models for DBS research.

In epilepsy research, chemoconvulsants (e.g., kainic acid, pilocarpine, pentylenetetrazole) are frequently used to generate rodents with spontaneously recurrent seizures [147]. These drugs induce extensive hippocampal sclerosis and mossy fibre sprouting in the dentate gyrus [148–150], and these models have strong face validity for temporal lobe epilepsy. Electrical kindling models can be generated by delivering chronic stimulation to the hippocampus or the amygdala, allowing for the screening and study of antiseizure treatments for chronic cases of epilepsy [151]. Genetic models are also very attractive to study epilepsy, providing strong construct validity as the mechanisms underlying epileptogenesis more accurately reflect the disease etiology in humans. However, the induction of seizures through chemoconvulsants is not representative of the clinical causes of epilepsy, kindled animals do not typically exhibit spontaneous recurrent seizures, thus limiting the translatability of findings, and transgenic animals may not exhibit the full range of complex alterations observed in patients.

Depression/anxiety are widely investigated models for DBS, having strong predictive and construct validity. Models of depression/anxiety can be generated through neurobiological and genetic manipulation (i.e., selective breeding of rats with a predisposition to depression or knockouts of serotonin-related genes) or via chronic exposure to stressors, which captures the delay in antidepressant efficacy seen in patients [152]. Additionally, these models lead to impairments in the hypothalamic-pituitary-adrenal axis, altered immune reaction, and changes in monoaminergic transmission similar to those detected in patients with depression [153]. Generally, DBS applied to rodent models of depression has demonstrated comparable effects to those observed in patients, such as increased monoamine concentrations in the prefrontal cortex (PFC) [154] and hippocampus [155] and normalization of local field potential activity at the ventral tegmental area (VTA) [156, 157]. Transgenic mice, but not rats, are also widely used in investigations of neurodevelopmental disorders. These include animals with mutations targeting genes known to be related to the disease in humans, such as *MECP2* [58–60]*, Shank3* [57], and *CDKL5* [61]. In contrast, rat studies focused on exposing naïve rats to chemicals to induce epigenetic changes [158] and subsequent ASD-like phenotypes [47–49] or injecting animals with patient-derived antinuclear antibodies to induce Tourette-like symptoms [50].

One major aspect of the experimental DBS landscape that requires dire attention is the need to include females in DBS studies. In this review, we found that only 1.9% of rat studies and 19.2% of mouse studies included females. The consideration of both sexes is important because DBS can have distinct effects on males and females due to differing biological attributes, such as genetics, physiology, brain anatomy, and hormones. For instance, several groups have reported behavioural and morphological differences among male and female 6-OHDA-lesioned rats [159, 160], which may subsequently affect DBS response. Similar observations have been made in models of addiction, whereby female rodents are more likely to consume and seek drugs in self-administration paradigms [161]. Thus, future experimental DBS studies should evolve to include both male and female models in order to address differing therapeutic effects influenced by sex.

### DBS parameters and electrode characteristics

DBS parameters such as amplitude, frequency, pulse width, and duration of treatment can vary across brain targets, disease indication, and electrode design. In the clinic, the ranges within which most stimulation parameters fall are in amplitudes between 1–3.5 V, frequencies between 80–185 Hz, and pulse width between 60–210 µs [162]. By means of translation to human DBS, more groups have started to apply clinically relevant DBS paradigms in rodent models. The use of high- or low-frequency stimulation to specific brain areas plays a large role in the overall effects of DBS in both rodent models and humans and may result in different local and widespread plastic brain changes that can be captured in studies evaluating electrophysiological and neurochemical responses associated with DBS [7]. For instance, high-frequency stimulation (HFS; >90 Hz) of the STN in PD models improves motor symptoms, whereas low-frequency stimulation (<50 Hz) is ineffective or exacerbates symptoms [163]. In contrast, although the majority of addiction studies employed HFS, some applying <20 Hz stimulation to the nAcc [25], lateral habenula [164], ventral striatum [165], or STN [67] demonstrated beneficial behavioural effects. In the same manner, there is debate regarding the use of intermittent vs. continuous stimulation. DBS has traditionally been delivered in a continuous fashion in the clinical setting; nonetheless, recent preclinical studies have suggested that a cyclic DBS programming approach may produce beneficial effects [55, 120]. Furthermore, several groups have begun to develop closed-loop adaptive DBS systems and advancements in this field (using both preclinical models and humans) will help us better understand the neural basis and overall effectiveness of such a technology.

The size, shape, and area of microelectrode implantation can affect the volume of tissue activated by stimulation and overall brain tissue reactivity or potential neural damage [166]. The large majority of DBS studies in rodents used bipolar stainless steel electrodes with a diameter between 125–300 µm for rats and 50–125 µm for mice. In recent years, there has been an increase in options for electrode materials such as platinum-iridium, tungsten, and carbon fibres. Platinum is a relatively non-toxic and biologically magnetic inert material in brain tissue and is similar to that used in humans [167]. Tungsten electrodes present low impedance, high conductivity, uniform plastic deformation, and MRI compatibility [168]. The use of carbon fibre electrodes for DBS and neural recording has also gained traction because of its ability to produce fewer MRI artifacts, improve magnetic field homogeneity, and induce smaller temperature changes in MR environments when compared to other metal-based electrodes [167, 169]. However, there are technical challenges surrounding the assembly and implantation of these flexible fibre electrodes, requiring a high level of surgical expertise [170].

### DBS brain targets

Regarding brain targets, there is currently no universal consensus on the best target for DBS, notwithstanding the disease indication. Thus, preclinical studies provide great value in our understanding and development of potential therapeutic targets. The most evident example is the DBS of the STN in PD, which is now an extensively used target in the clinic. The STN was found to exhibit unusually increased activity in animals with Parkinsonian symptoms, and experimental lesions of the STN in rats resulted in evident improvements in motor dysfunction [171]. These findings supported the hypothesis that pathological activity occurs in the STN in PD, and modulation of this area can improve Parkinsonian symptoms. Accordingly, studies using rodent models have demonstrated that STN-DBS significantly reduces tremors, rigidity, and bradykinesia associated with PD. However, consistent with clinical observations, some rodents treated with STN-DBS also exhibited enhanced depressive-like behaviours [68, 69, 172]. Thus, other key brain structures, such as the entopeduncular nucleus [35, 173], pedunculopontine tegmental nucleus [116, 174–177], substantia nigra [178, 179], and zona incerta [172] have been considered for DBS targeting in movement disorder rodent models in hopes of alleviating non-motor symptoms.

Epilepsy research has concentrated on stimulating various aspects of the thalamus to alleviate seizure activity. Thalamic nuclei are highly interconnected with substantial bidirectional projections via the mammillothalamic, thalamocortical, and spinothalamic tracts. Thus, thalamic DBS has been shown to significantly reduce seizure frequency and increase seizure latency, as detected using electroencephalography [180]. DBS targeting the thalamus has also been studied in the context of AD, whereby high-frequency stimulation was shown to be less effective at facilitating spatial memory than the entorhinal cortex or fornix DBS [141]. In line with this finding, Hamani et al. [128]. reported that DBS delivered to the ANT at high amplitude (i.e., 500 µA) disrupts the acquisition of contextual fear conditioning and, in turn, may explain the impaired performance on spatial alternating tasks observed in these rats. In contrast, a recent study by Chamaa et al. showed that both single and repeated sessions of ANT-DBS may induce a significant increase in neurogenesis in the ipsilateral dentate gyrus [181] and improve spatial reference memory on the Y-maze test [182].

In the field of psychiatric disorders, the nAcc is a key DBS target explored for the treatment of SUD, eating disorders, treatment-resistant depression, OCD, and psychosis. Rodents receiving nAcc stimulation show a decrease in consumption, preference, and/or motivation for consuming several substances such as ethanol [26, 106, 183, 184], cocaine [24, 25, 67, 91, 92, 164, 185, 186], and morphine [93, 112, 187–190]. While most studies showed success in modulating pathologic behaviour using nAcc-DBS, there was conflicting evidence regarding the efficacy of stimulating the nAcc core vs. shell. Several addiction studies showed reduced morphine-induced conditioned place preference and ethanol self-administration regardless of whether the DBS was targeting the nAcc core or shell. In contrast, Vassoler et al. [91] and Wilden et al. [183] demonstrated that DBS of the nAcc shell, but not the nAcc core, reduced cocaine-induced reinstatement. Similar to this observation, Oterdoom et al. [191] reported a reduction in binge-eating behaviour among rats treated with DBS targeting the nAcc shell but not in those targeted at the core aspect of the nucleus. Furthermore, Sesia et al. [21] reported reduced impulsivity when targeting the nAcc core and the opposite effect when targeting the nAcc shell of OCD models. The contradicting findings regarding the optimal DBS target may be explained by the variation in volumes of tissue activated due to differing stimulation parameters applied and differences in cellular characteristics between models.

### Mechanisms of action

In rodent DBS research, the most frequent study goal is to offer a better understanding of the mechanisms of action of treatment. Several hypotheses on the mechanism of DBS have been proposed, such as direct inhibition/excitation of neural activity, whereby DBS modulates pathological oscillatory activity within brain networks [7]. However, the precise action of DBS in each brain target and in each neurological or psychiatric disorder remains elusive.

#### Parkinson’s disease

DBS is considered the standard of care for patients with PD, and the STN represents the most well-studied target for this indication. Several studies describe a neuroprotective effect of STN-DBS on dopaminergic neurons in the substantia nigra [30, 31, 192–194] via increased signalling strength in the BDNF-trkB pathway (i.e., brain-derived neurotrophic factor and its receptor tropomyosin receptor kinase B), and increased autophagy through the blocking of protein phosphatase 2A activation [195, 196]. However, these results failed to be replicated in transgenic models of α-Synuclein pathology [197]. Increased levels of BDNF and cerebral dopamine neurotrophic factor in the nigrostriatal system and primary motor cortex have also been associated with positive outcomes following STN-DBS [115, 195, 198–200]. Furthermore, STN-DBS is thought to reduce neuroinflammation [201–203] due to decreased fractalkine pathway signalling [201–203]. Conflicting results have been reported on extracellular levels of dopamine following STN-DBS [204–208], with some studies showing the increase in dopamine metabolites to be associated with a better outcome [204–208]. STN-DBS has also been reported to inhibit serotonergic neurons in the raphe nuclei [29, 209–211] and decrease serotonergic transmission in the striatum, hippocampus, and PFC [212, 213], leading to depression [109, 172, 212, 213]. This effect was not observed following entopeduncular nucleus DBS, suggesting that the therapeutic effect of DBS via decreased serotonergic transmission is characteristic of STN-DBS [214, 215]. Electrophysiological studies have associated decreased beta oscillations in cortical and basal ganglia networks [43, 208, 211, 216, 217] and increased alpha power in the medial PFC [218] with improved motor and non-motor symptoms following treatment. In models of motor symptoms not associated with dopamine degeneration (e.g., haloperidol-induced catalepsy, pharmacologically-induced tremor and tardive dyskinesia [219, 220]), STN-DBS improves motor function through the modulation of serotonin transmission hippocampal-striatal coherence, and cortical beta oscillations [29, 209–211].

#### Dementias and Alzheimer’s disease

DBS represents an emerging therapeutic approach for patients with dementia. Improved memory and enhanced hippocampal neurogenesis in the dentate gyrus have been reported following DBS of several brain targets, including the fornix [54, 66], nucleus basalis of Meynert (NBM) [221], medial septum [142, 222], entorhinal cortex (EC) [144], ventral striatum [223], and thalamus [181, 182], and this effect was associated with the restoration of theta oscillations in thalamic nuclei and striatum following treatment [224]. Fornix- and NBM-DBS have also been shown to reduce hippocampal neuronal loss and neuroinflammation [54, 66], whereby fornix-DBS increased hippocampal acetylcholine levels and activity [142, 225]. NBM-DBS promoted cholinergic fibre growth in the cingulate cortex without altering hippocampal acetylcholine levels [221]. Additionally, DBS of the intralaminar thalamic nuclei leads to increased dendritic spine density in cortical and hippocampal pyramidal neurons [64, 138, 221] and altered cortical glutamatergic neurotransmission [226]. The neuropathological hallmarks of AD have also been explored following EC- and fornix-DBS treatments. While both EC- and fornix-DBS reduced cortical and hippocampal amyloid deposition [54, 143, 144, 146], EC-DBS also decreased tau protein levels in the cortex and hippocampus [143, 144, 146] by enhancing enzymes involved in tau clearance [143, 144, 146].

#### Epilepsy

The ANT is the primary target for treating epilepsy with DBS, as it is approved by the Food and Drug Administration as an adjunctive treatment for reducing the frequency of partial-onset seizures in adult populations [227]. Positive changes in the hippocampus following ANT-DBS have been shown, with enhanced neurogenesis, increased neurotrophic factors, reduced neuroinflammation, reduced mossy fibre sprouting, and anti-apoptotic and neuroprotective effects [75–80, 228–230]. ANT-DBS anti-seizure effect was also associated with increased adenosine, reduced adenosine kinase levels and changes in gamma-aminobutyric acid (GABA) transmission in the hippocampus [230–234]. Differentially expressed genes involved in ion channel activity, glutamatergic synapse, and regulation of immune response have also been reported in the hippocampus following ANT-DBS [235]. Electrophysiological studies described ANT-DBS to enhance the seizure threshold by decreasing theta oscillations in the hippocampus and cortex and by suppressing delta oscillations during non-rapid eye movement sleep, which results in an increased amount of rapid eye movement sleep [82, 230, 234].

#### Addiction

It is widely accepted that the initial reinforcing effects of addictive drugs are mediated by enhanced synaptic concentrations of dopamine in forebrain subcortical structures, particularly the nAcc [236, 237], and the prolonged use of drugs leads to an imbalance between excitatory glutamate and inhibitory GABA [236, 238]. Preclinical studies on nAcc-DBS for the treatment of addiction have shown antidromic stimulation of the cortex via cortico-accumbal afferents [91, 165, 239], decreased glutamate and increased GABA levels in the VTA, ventral pallidum, and nAcc [190], upregulation of glutamatergic receptors in the nAcc [105] and amygdala [240], and downregulation of glutamatergic receptors in the VTA [164]. Interestingly, the selective blockade of dopamine 1 receptor (D1R) along with nAcc-DBS reverses cocaine-induced hyperlocomotion and plasticity changes in GABAergic medium spiny neurons expressing D1R via activation of metabotropic glutamatergic receptors [105], suggesting an intricate interaction between neurotransmitter systems is required for the therapeutic effects of DBS.

#### Eating Disorder

DBS treatment for eating disorders has been described in patients suffering from morbid obesity. The nAcc and the ventral and/or lateral aspects of the hypothalamus are the targets of choice in this patient population, and although DBS was considered to be a safe procedure with encouraging outcomes, it has been associated with side effects such as hardware infection and stimulation-induced mania [241, 242]. nAcc-DBS in rodent models of obesity is associated with decreased high fat/ high sucrose intake [104, 191], improved glucagon and glucose concentrations in plasma and increased neuronal activation in the lateral hypothalamus [243]. Lateral hypothalamus DBS led to reduced body weight and food intake [244, 245], increased metabolism in the mammillary body, hippocampus, and amygdala, and decreased metabolism in the thalamus, caudate, temporal cortex, and cerebellum [245]. This treatment was also associated with reduced markers of activity-induced synaptic plasticity and memory formation in the hippocampus and EC and vesicular GABA transporter [246].

#### Obsessive-compulsive disorder

Changes in the mesolimbic dopaminergic reward system play a significant role in the pathological habit formation observed in OCD [236, 237]. In clinical populations, the anterior limb of the internal capsule, the nAcc, and the inferior thalamic peduncle are common targets for neuromodulatory treatments of drug-resistant OCD [247–249]. In rodent models of OCD, DBS targeting the internal capsule led to increased neuronal activity in the targeted area and in the PFC, which was associated with therapeutic effects [62]. Similarly, DBS of the ventral striatum increased neuronal activity in the targeted area and in the PFC, orbitofrontal cortex (OFC), and amygdala [62, 123]. When targeting the core aspect of the nAcc specifically, DBS reduced OFC neuronal firing and enhanced spontaneous local field potential oscillatory activity in the slow frequency band [239, 250]. nAcc-DBS was also shown to increase dopaminergic and serotonergic in nAcc [21]. Thalamic DBS, however, has a distinct behavioural outcome when different subnuclei are targeted; while DBS of the mediodorsal nucleus of the thalamus increases impulsivity and decreases neuronal activity in cerebellar nuclei and PFC [22], DBS of the parafascicular nucleus of the thalamus alleviates prepulse inhibition [251], a measure of sensorimotor gating which is disrupted in OCD [252, 253]

#### Depression

Given its key role in emotional processing and depressotypic behaviour, the infralimbic (IL) nucleus (also referred to as the mouse ventromedial PFC, a structure considered to be homologous to the human subgenual cingulum) is the most commonly studied DBS target in models of depression. IL-DBS was shown to enhance serotonin release and induce long-term alterations of serotonin receptor expression [85, 254, 255], an effect that can be blocked by depleting serotonergic transmission [255], and is thought to be mediated by direct modulation of prefrontal projections to the dorsal raphe nucleus [254–257]. Enhanced levels of serotonin were also reported following DBS of the nAcc [258–260], lateral habenula, and medial forebrain bundle [261, 262]. In addition to serotonergic transmission, IL-DBS is also modulated by adenosinergic and glutamatergic neurotransmission as pre-treatment with selective antagonists attenuates the antidepressant effect of treatment [257, 263]. Furthermore, IL-DBS induces beneficial neuroplasticity, such as hippocampal neurogenesis [264, 265], and increased dendrite length [154, 266], with associated increases in BDNF [264, 265, 267–270].

#### Post-traumatic stress disorder

The amygdala and IL have been the primary DBS targets for treating PTSD, as hyperactivity of the basolateral amygdala (BLA) is associated with PTSD symptom severity [271]. BLA-DBS has been shown to decrease and normalize local neuronal activation [272, 273] and reverse fear condition-induced changes to synaptic plasticity in the cortical-amygdala connections, suggesting BLA-DBS may disrupt the long-term retention of fear memory [274]. However, it is important to note that the chronic electrical stimulation of the temporal lobes (especially the amygdala) is known to induce seizures, therefore being a well-established model of kindling [275, 276]. IL-DBS was shown to mitigate PTSD-like behaviours while reducing BLA neuronal activity by activating GABAergic interneurons through PFC-BLA projections [100], being an effective and safer target for DBS.

#### Neurodevelopmental disorders

ASD and Tourette’s syndrome are the most commonly studied neurodevelopmental disorders in preclinical DBS research. In ASD models, IL-DBS restores the abnormal serotonergic transmission and modulates the expression of glutamatergic and GABAergic receptors in the targeted area while improving social deficits, anxiety-like behaviour, and hyperactivity [48]. Fornix-DBS in ASD models shows an antidepressant effect [58] and reduces memory deficits [60, 61] via normalization of the expression of genes related to intellectual disabilities [58] and restoration of hippocampal synchrony, spontaneous excitatory postsynaptic currents, long-term potentiation and feedforward inhibition [59–61]. Thalamus-DBS has been shown to improve repetitive behaviours in ASD models by restoring functional connectivity in corticostriatal and corticolimbic circuits and decreasing dopaminergic receptor expression in the striatum [47]. In a Tourette’s syndrome model, however, thalamus-DBS reduced local theta band activity and local field potentials in the striatum [49]. In this model, entopeduncular DBS decreased dopamine concentration and dopamine transporter in the striatum, leading to decreased tic behaviour [50].

#### Schizophrenia and psychosis

Several targets have been investigated for improving positive symptoms in models of schizophrenia and psychosis. While targeting the hippocampus with DBS restores the dopaminergic activity of the VTA [277], targeting the VTA with DBS leads to increased GABA neurotransmission but not dopaminergic activity [278]. Medial septum-DBS has also been shown to restore dopaminergic transmission while improving serotonergic activity [94, 95] and reducing hippocampal gamma oscillations [20], which leads to attenuated psychotic schizophrenic symptoms. Similarly, thalamic-DBS modulates neural oscillations, alleviating aberrant thalamic oscillatory activity and modulating coherence in thalamocortical networks [279].

#### Traumatic Brain Injury

In the context of TBI, DBS has been used to address decreased levels of consciousness, deficits in cognitive function, and motor recovery. The hypothalamus is a key region involved with wakefulness and alertness, particularly through orexin neurons of the lateral hypothalamus that have widespread projections throughout the brain [280]. In a weight-drop model of TBI, lateral hypothalamus-DBS promoted consciousness recovery along with increases in adenosine A1 receptor, decreases in GABA receptor, increases in orexin receptor, and decreased low-frequency delta oscillations in the PFC [280]. In addition, brain regions with strong connectivity with the hippocampus, such as the medial septum, have also been targeted, as these regions play a critical role in cognition. DBS of the medial septum was linked to the restoration of hippocampal theta oscillations and improved cognitive functions following TBI [281]. Furthermore, the cerebellum has been targeted for its role in influencing motor function through extensive thalamocortical projections, and DBS of the cerebellum was shown to enhance motor functions alongside the elevation of perilesional neuronal activity and suppression of neuroinflammation and apoptotic markers [45].

### Limitations of DBS studies with rodent models

A few limitations need to be taken into account when considering the direct translation of data from rodent models to the clinical setting. Although experimental models have provided much value to our understanding of the underlying mechanisms and effects of DBS, it remains important to consider that these models do not fully reflect human disease but rather mimic a series of key symptoms. For instance, DBS of the fornix and lateral hypothalamus has shown promising outcomes in rodent models of AD [54, 111] and obesity [244, 245], respectively. However, the application of these approaches has not been as successful within the clinical population [282, 283], which alludes to the caution that needs to be taken when translating such therapeutic regimens to humans. The high rate of positive behavioural responses observed in these studies should also be interpreted with caution, as there is a tendency in scientific publications to focus on publishing positive results rather than negative ones. Furthermore, the anatomical organization of rodent brains is similar to that of the human brain in many aspects; however, key differences in cortical processing and, therefore, cognitive abilities prevent the direct translation of findings, especially in the context of top-down inhibitory control of motivated behaviours. Also, anatomical differences between rodents and humans in specific clinically relevant brain targets may be impeditive to a translation to rodent models. Lastly, the make of DBS electrodes, the stimulation parameters and stimulation settings (i.e., acute, chronic) used in rodents are highly variable and are typically different from those used in humans [284].

Despite these limitations, rodent studies have contributed substantially to our current knowledge of the mechanisms underlying DBS treatment. Advancements in the field have led to the development of novel stimulation techniques and rodent DBS devices that are fully implantable and/or with wireless stimulators for long-term use in freely moving animals [285]. These applications are only growing as new technologies such as closed-loop circuits [286] and specialized stimulation electrodes [167–169] improve the precision of targeting and decrease adverse effects.

### Ethical considerations

While DBS is an efficacious surgical treatment for many neurological and psychiatric disorders in adult and pediatric populations, the precise neurobiological mechanism of action of DBS treatment remains elusive. Thus, translational research is needed to advance our understanding of disease processes and treatment mechanisms to develop novel, less invasive and more efficacious therapies. Animal experimentation, however, must be performed under strict ethical guidelines to answer sound scientific questions that cannot be addressed in in-vitro or in-silico models [287–289]. The “Three Rs” principle (Reduction, Refinement, and Replacement) sets the ethical standard for in-vivo research. The reduction principle refers to reducing the number of animals used per experiment or study while keeping statistical power, the Refinement principle refers to the improvement of all methods used in research to minimize pain, suffering, distress or lasting harm and improve animal welfare, and the Replacement principle refers to the full or partial replacement of live animals with technologies or alternative approaches [287–289].

In clinical settings, distinct ethical considerations must be made when performing DBS trials. Questions on how to provide the best care for vulnerable patients at the late stages of life, especially those with progressive degenerative disorders, must be made to assess if the benefits of treatment are superior to the potential complications [290, 291]. In patients with psychiatric disorders, major concerns arise related to the patient’s capacity to provide informed consent, the possibility of altering behaviour via brain stimulation, as well as the risks and benefits compared to traditional and less invasive treatment methods [292, 293]. Also, DBS for treating psychiatric disorders shares many features with psychosurgeries and, therefore, raises several ethical and legal concerns that must be openly discussed [294]. For the pediatric population, it is crucial to assert the potential risks and benefits of DBS, the optimal time for offering the treatment and the long-term consequences of brain stimulation [295]. Also, it is necessary to discuss the role of the informed assent given by the child in addition to the informed consent signed by the parent/guardian [295]. A bioethical framework to advance ethical discussions regarding pediatric DBS has been proposed and involves the protection of the child’s best interest, the consideration of the developmental context, the creation of strategies for mitigating known and unknown risks, the critical appraisal of the adult literature, and fostering communication and collaboration among practitioners [296]. Finally, there are several concerns related to specific devices’ capacity for detecting neuronal activity and the safe storage of these data [297]. With the advancement of DBS devices, new policies must be developed to maximize benefits and minimize harm to patients.

## Conclusion

Though there has been extensive research into the effects of DBS targeted to key brain structures, there remains a dire need for standardized, protocol-based approaches to find optimal stimulation targets and parameters for distinct pathologies. Studies on rodent models have not only shed light on possible mechanisms of action of DBS treatment but also improved our understanding of the underlying disease processes. Models of psychiatric and neurodevelopmental disorders have implicated dysfunctions in the monoaminergic system and in the functional connectivity of the mesocorticolimbic network in the pathophysiology of diseases, and, therefore, modulation of these aspects with DBS resulted in improved behaviour. Similarly, the cellular and molecular complexity of PD has been further explored in rodent models, which are capable of capturing both motor and non-motor symptoms of the disease. In these models, DBS improves symptoms via modulation of the striatal dopaminergic system and suggests that treatment response is associated with dopamine availability. Although there are several limitations in translating findings from preclinical to clinical settings, these studies have shown that direct modulation of neural activity (both cellular and molecular) is among the major mechanisms of action, albeit further research is necessary for a complete understanding of this neuromodulation therapy.

### Supplementary information

Supplememntary Figure 1

Supplementary Tables

Supplementary References

## Data Availability

All articles included here are available in PubMed, and the complete reference list can be found in the Supplementary References.
