# Supplementary material for: Systematic review of rodent studies of deep brain stimulation for the treatment of neurological, developmental and neuropsychiatric disorders
Source: Transl Psychiatry. 2024 Apr 11;14:186. doi: 10.1038/s41398-023-02727-5 (PMC11009311; doi:10.1038/s41398-023-02727-5)
Supplement: Supplementary file 1 — Supplememntary Figure 1 [file 41398_2023_2727_MOESM1_ESM.pdf]

Kristina K. Zhang, Rafi Matin, Carolina Gorodetsky, George M. Ibrahim, Flavia Venetucci Gouveia

SUPPLEMENTARY FIGURE

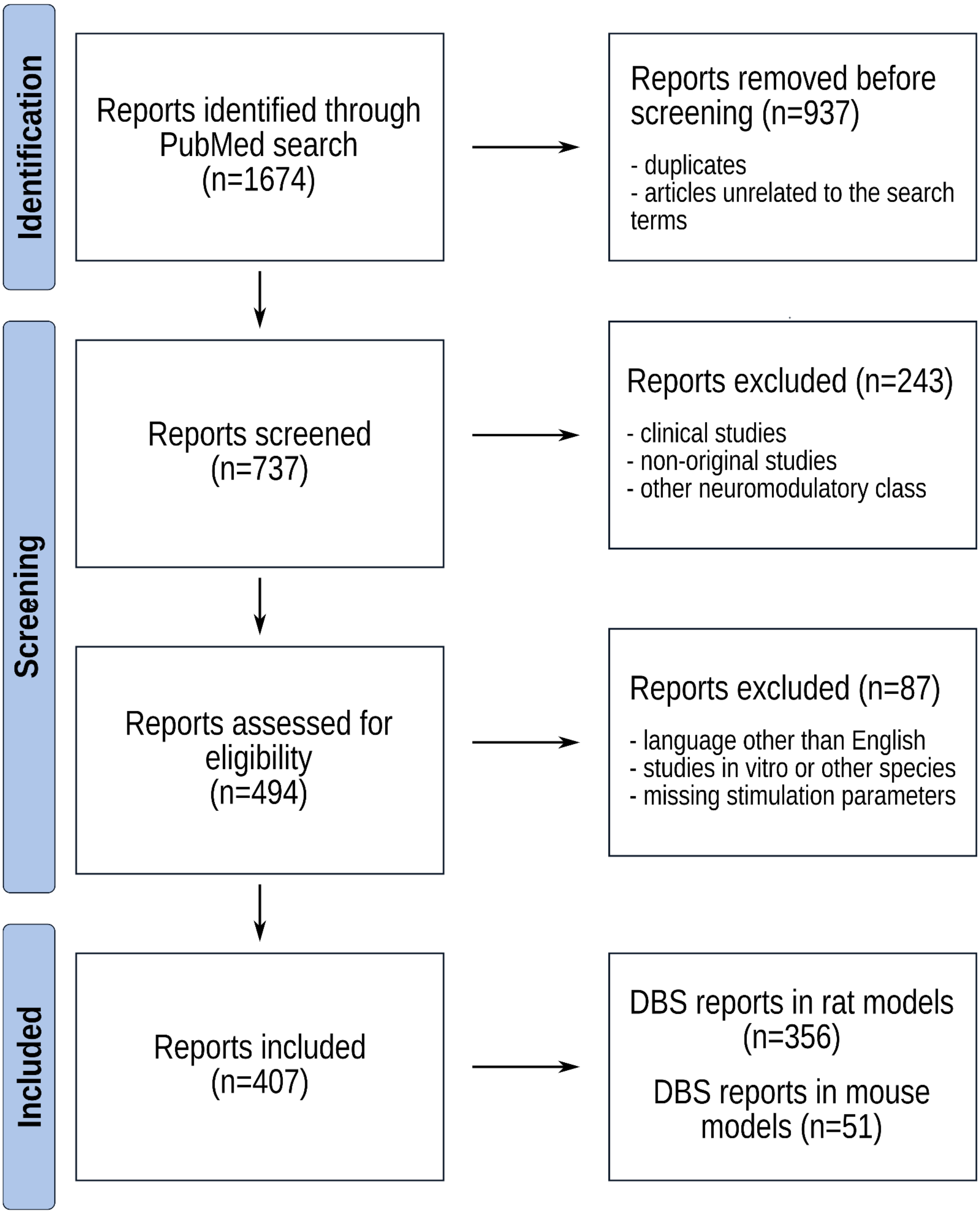

Supplementary Figure 1. PRISMA Flowchart. Flow diagram detailing the systematic review process performed in this study. Abbreviations: DBS: deep brain stimulation; PRISMA: Preferred Reporting Items for Systematic Reviews and Meta-Analyses.
