# Supplementary material for: Systematic review of rodent studies of deep brain stimulation for the treatment of neurological, developmental and neuropsychiatric disorders
Source: Transl Psychiatry. 2024 Apr 11;14:186. doi: 10.1038/s41398-023-02727-5 (PMC11009311; doi:10.1038/s41398-023-02727-5)
Supplement: Supplementary file 2 — Supplementary Tables [file 41398_2023_2727_MOESM2_ESM.pdf]

SUPPLEMENTARY TABLES

Supplementary Table 1. Classification of rodent models

Disease model class	Disease model subclass for rats	Disease model subclass for mice
Anxiety disorders	No subclass	
Bladder function	No subclass	Not studied in mice
Dementia/Cognition	Alzheimer's disease<br>Dementia<br>Cognitive performance	Alzheimer's disease<br>Dementia<br>Cognitive performance
Depression	No subclass	
Eating disorders	Food intake<br>Hedonic feeding<br>Glucose metabolism<br>Obesity	Hedonic feeding<br>Obesity
Epilepsy	No subclass	
Movement disorders	Dyskinesia<br>Motor impairment (catalepsy)<br>Parkinson's disease<br>Tremor	Ataxia<br>Parkinson's disease<br>Tremor
Neurodevelopmental disorders	Autism Spectrum Disorder<br>Tourette syndrome	Autism Spectrum Disorder<br>Rett syndrome
Obsessive-compulsive disorder	No subclass	
Physical injury	Ischemia<br>Pain<br>Spinal cord injury<br>Stroke<br>Traumatic brain injury	Stroke<br>Traumatic brain injury
Psychosis	No subclass	
Sleep-wake disorders	Sleep cycle	Narcolepsy
Substance use disorder	Alcohol<br>Cocaine<br>Heroin<br>Methamphetamine<br>Morphine	Alcohol<br>Cocaine
Tinnitus	No subclass	
Trauma/stressor-related disorders	Fear<br>Post-traumatic stress disorder	Not studied in mice

Supplementary Table 2. Summary of brain targets, rodent strains, efficacy, and stimulation parameters from studies of deep brain stimulation in rat and mouse models.

**Disease Model (No. of articles included)**	**Brain targets**	**Rodent strain**	**No. articles reporting symptom improvement**	**Average DBS parameters**		
				**Amplitude**	**Frequency (Hz)**	**Pulse Width (µs)**
Anxiety Disorder (8)	- BNST<br>- Caudate n.<br>- Dorsal Raphe n.<br>- Hypothalamus<br>- Internal capsule<br>- n. Accumbens<br>- Periaqueductal grey<br>- Frontal cortex<br>- Ventral striatum	- 129S1/SvImJ<br>- C67BL/6<br>- Sprague-Dawley<br>- Wistar	7 (87.5%)	Rat<br>183.3µA<br><br>Mouse<br>100µA; 3V	Rat<br>111.4<br><br>Mouse<br>84.0	Rat<br>162.9<br><br>Mouse<br>56.7
Bladder function (1)	- Locus coeruleus<br>- Periaqueductal grey<br>- Pedunculopontine tegmental n.<br>- Pontine reticular n.	- Sprague-Dawley	1 (100%)	Rat<br>2.5V	Rat<br>50	Rat<br>500
Dementia/Cognition (29)	- Anterior thalamic n.<br>- Entorhinal cortex<br>- Entopeduncular n.<br>- Fornix<br>- Frontal cortex<br>- Hippocampus<br>- Medial septum<br>- n. basalis of Meynert<br>- Subthalamic n.<br>- Ventral striatum	- 3xTg<br>- APP/PS1<br>- C57BL/6<br>- Sprague-Dawley<br>- TgCRN8<br>- tgHD<br>- Wistar	17 (59%)	Rat<br>155.1µA<br><br>Mouse<br>137.5 µA	Rat<br>83.9<br><br>Mouse<br>93.5	Rat<br>113.7<br><br>Mouse<br>91.7
Depression (56)	- Caudate/putamen<br>- Cerebellar vermis<br>- Cingulate cortex<br>- Entopeduncular n.<br>- Frontal cortex<br>- Lateral habenula<br>- Medial forebrain bundle<br>- n. Accumbens<br>- Subthalamic n.<br>- Ventral tegmental area	- BALB/c<br>- FSL<br>- Pet1-tdTomato<br>- Sprague-Dawley	48 (83%)	Rat<br>200.7µA; 2.3V<br><br>Mouse<br>110µA; 2.5V	Rat<br>106.6<br><br>Mouse<br>130.0	Rat<br>111.4<br><br>Mouse<br>81.7
Eating Disorder (14)	- Hypothalamus<br>- n. Accumbens<br>- Ventral tegmental area	- C57BL/6<br>- Sprague-Dawley<br>- Wistar<br>- Zucker	5 (36%)	Rat<br>159.3µA; 2V<br><br>Mouse<br>108.3µA	Rat<br>122.0<br><br>Mouse<br>145	Rat<br>85<br><br>Mouse<br>75
Epilepsy (40)	- Basolateral amygdala<br>- Hippocampus<br>- Medial septum<br>- Olfactory bulb<br>- Thalamus<br>- Substantia nigra<br>- Ventral pallidum	- C57BL/6<br>- Q54<br>- GAERS<br>- Sprague-Dawley<br>- WAG/Rij<br>- Wistar	36 (88%)	Rat<br>254.9µA; 1V<br><br>Mouse<br>100µA; 2V	Rat<br>109.1<br><br>Mouse<br>43.2	Rat<br>96.4<br><br>Mouse<br>96.7
Healthy strains (67)	- Entorhinal cortex<br>- Entopeduncular n.<br>- Dorsal Raphe n.<br>- Fornix<br>- Frontal cortex<br>- Globus pallidum<br>- Internal capsule	- BALB/c<br>- C57BL/6<br>- ePet-cre<br>- Fischer<br>- Lewis<br>- Long Evans<br>- Sprague-Dawley	--	Rat<br>229.6µA; 5.1V<br><br>Mouse<br>91.4µA; 2.75V	Rat<br>118.7<br><br>Mouse<br>139.4	Rat<br>143.4<br><br>Mouse<br>142.2

	- Locus coeruleus<br>- Medial septum<br>- Medial forebrain bundle<br>- Motor cortex<br>- n. Accumbens<br>- Periaqueductal grey<br>- Pedunculopontine tegmental n.<br>- Pontine reticular n.<br>- Substantia nigra<br>- Thalamus	- Wistar				
Movement Disorder<br>(104)	- Cerebellar dentate n.<br>- Entopeduncular n.<br>- Globus pallidum<br>- Hypothalamus<br>- Inferior colliculus<br>- Parietal association cortex<br>- Pedunculopontine tegmental n.<br>- Substantia nigra<br>- Thalamus<br>- Ventral striatum<br>- Zona incerta	- C57BL/6<br>- Car8wdl<br>- Drd1a BacTRAP<br>- Drd2 BacTRAP<br>- Fischer<br>- FVB/NJ<br>- Lewis<br>- Listar hooded<br>- Long Evans<br>- Sprague-Dawley<br>- Wistar	84 (81%)	Rat<br>159µA;<br>1.6V<br><br>Mouse<br>90.9µA	Rat<br>110<br><br>Mouse<br>125	Rat<br>84.3<br><br>Mouse<br>83
Neurodevelopmental Disorder<br>(9)	- Entopeduncular n.<br>- Fornix<br>- Frontal cortex<br>- Thalamus	- CDKL5$^{-/-}$<br>-Mecp2$^{-/-}$<br>- Sprague-Dawley<br>- Shank3B$^{-/-}$<br>- Wistar<br>- Viaat-Mecp2$^{-/y}$	9 (100%)	Rat<br>265µA<br><br>Mouse<br>40µA	Rat<br>130.0<br><br>Mouse<br>130	Rat<br>76.3<br><br>Mouse<br>60
Obsessive-compulsive Disorder<br>(10)	- Entopeduncular n.<br>- Internal capsule<br>- n. Accumbes<br>- Thalamus<br>- Ventral striatum	- Lewis<br>- Long Evans<br>- Sapap3$^{-/-}$<br>- Sprague-Dawley<br>- Wistar	8 (80%)	Rat<br>112.2µA<br><br>Mouse<br>300µA	Rat<br>130<br><br>Mouse<br>120	Rat<br>91.1<br><br>Mouse<br>80
Physical injury<br>(17)	- Cuneiform n.<br>- Dorsal Raphe n.<br>- Entorhinal cortex<br>- Hippocampus<br>- Hypothalamus<br>- Locus coeruleus<br>- Mesencephalic locomotor n.<br>- Posterior insula<br>- Pedunculopontine tegmental n.<br>- Thalamus	- C57BL/6<br>- Lewis<br>- Long Evans<br>- Sprague-Dawley<br>- Wistar	13 (100%)	Rat<br>100µA;<br>2.1V<br><br>Mouse<br>150µA; 4V	Rat<br>84.9<br><br>Mouse<br>120	Rat<br>201<br><br>Mouse<br>145
Psychosis<br>(10)	- Entopeduncular n.<br>- Frontal cortex<br>- Hippocampus<br>- Medial septum<br>- n. Accumbens<br>- Thalamus<br>- Ventral tegmental area	- DAT-ires-cre<br>- Long Evans<br>- Sprague-Dawley<br>- Vgat-ires-cre<br>- Wistar	9 (100%)	Rat<br>175µA<br><br>Mouse<br>100µA	Rat<br>109.4<br><br>Mouse<br>130	Rat<br>108.8<br><br>Mouse<br>60
Sleep-wake Disorders<br>(2)	- Entopeduncular n.<br>- Hypothalamus<br>- Zona incerta	- Sprague-Dawley<br>- Tg(HCRT-MJD)^{1}Stak	2 (100%)	Rat<br>ND<br><br>Mouse<br>2.5V	Rat<br>100<br><br>Mouse<br>15	Rat<br>ND<br><br>Mouse<br>ND
Substance	- Amygdala	- C57BL/6	26 (90%)	Rat	Rat	Rat

abuse/addictive Disorders (29)	- Anterior insula<br>- Hippocampus<br>- Hypothalamus<br>- Lateral habenula<br>- n. Accumbens<br>- Orbitofrontal cortex<br>- Substantia nigra<br>- Ventral pallidum<br>- Ventral striatum<br>- Ventral tegmental area	- Lister hooded<br>- Long Evans<br>- Sprague-Dawley<br>- Wistar		162.9µA; 2V<br><br>Mouse 200µA	105.1<br><br>Mouse 88.3	119.2<br><br>Mouse 45.8
Tinnitus (5)	- Caudate n.<br>- Cerebellar dentate n.<br>- Inferior colliculus<br>- Thalamus	- Sprague-Dawley	2 (40%)	Rat 96.4 µA	Rat 82	Rat 44.3
Trauma/Stress-related Disorders (6)	- Amygdala<br>- Frontal cortex	- Sprague-Dawley<br>- Wistar	6 (100%)	Rat 200µA; 2.6V	Rat 140	Rat 132.5

Supplementary Table 3. Validity of rodent models of disease.

Disorder	Models	Face validity	Construct validity	Predictive validity	Main limitations	Description
Anxiety Disorder	Behavioural tests (i.e., EPM, elevated T-maze, OF, light/dark box, etc)	+	-	+	- Potential for learned behaviours to influence performance<br>- Lack of specificity, easily influenced by external factors<br>- Lack of construct validity	Etiologically relevant (anxiety-like levels in response to new environments, innate avoidance, and/or aversive stimuli) behaviours with pathophysiological changes such as altered serotonin system and HPA axis; Responsive to anxiolytic drugs and/or therapies
	Chronic unpredictable stress	+	+	+	- Difficult to reproduce<br>- Easily influenced by stressor type and stress exposure length	
	Fear conditioning	+	+	+	- Susceptible to rapid extinction process<br>- Influenced by strain, cue type and magnitude, and aversive conditioning levels	
Dementia	Spontaneous (i.e., age-induced)	+	+	+	- Difficulty in assessing whether memory deficits are caused by aging or other background factors<br>- High inter-animal variability in onset of age-related cognitive deficits<br>- Slow and prolonged experimental timeline	Induce memory deficits; Pathophysiological changes such as neuronal loss, synaptic dysfunction and increased beta-amyloid deposition and tau accumulation in regions involved with memory/ cognition; Responsive to cholinesterase inhibitors and glutamate receptor antagonists among other prevention strategies.
	Chemically induced (i.e., scopolamine, amyloid b-peptide, 192 IgG-saporin)	+	+	+	- Do not represent natural etiology of disorder	
	Genetic manipulation (i.e. transgenic)	+	+	+	- Focus on specific aspect of disorder, resulting in a limited set histopathological changes	
Depression	Chronic stressors (i.e. chronic unpredictable stress, learned helplessness, maternal deprivation)	+	+	+	- Difficult to reproduce<br>- Easily influenced by stressor type and stress exposure length	Behaviours comparable to symptoms observed in patients with depression (i.e., despair, anhedonia); Pathophysiological changes such as altered HPA axis, monoamine systems, and corticosterone; Responsive to antidepressant drugs and electroconvulsive therapy.
	FST, TST	-	-	+	- Lack of face and construct validity<br>- Short-term stress, detects acute responses to treatments	
	Olfactory	+	+	+	- Enhanced	

	bulbectomy				hyperactivity in females<br>- Potential for sensory deficits independent of depression-related mechanisms<br>- Short-term behavioural changes	
	Genetic manipulation (i.e., selective breeding, transgenic)	+	+	+	- Does not capture polygenic nature of depression	
Eating Disorder - Obesity	Diet-induced	+	+	+	- Intervention time required for obesity development varies widely<br>- Different biological effect depending on type of diet administered	Exhibit enhanced food intake or body weight; Display some comorbidities such as hyperglycemia, insulin resistance, or diabetes-like syndromes; Responsive to anti-obesity drugs and therapies
	Zucker rat	+	+	+	- Variability in disease progression<br>- Comorbid selective memory impairment	
Epilepsy	Chemoconvulsive agents (i.e. Pilocarpine, Kainic acid, PTZ)	+	+	+	- Do not represent natural etiology of disorder	Induce electrographic and behavioral seizures; Generate pathological alterations in the hippocampus, amygdala, and cortical regions; Can be used to model both epileptogenesis and chronic state; Responsive to anti-seizure medication
	Kindling (i.e. electrical, chemical)	+	+	+	- Electrical kindling requires surgical procedure<br>- Do not represent natural etiology of disorder	
	Genetic manipulation (i.e., selective breeding, transgenic)	+	+	+	- Monogenic mutations do not account for complexity of disorder<br>- Complex genetic environment induced by polygenic mutations can introduce confounds	
Parkinson's Disease	6-OHDA	+	+	+	- Does not capture full range of aberrant changes observed in PD<br>- Does not capture the progressive and gradual degeneration of neurons observed in PD	Induce motor deficits associated with PD (i.e., tremors, rigidity, bradykinesia); Model the depletion of striatal DA; Responsive to PD treatments with similar side effects such as dyskinesia
	Alpha-synuclein overexpression	+	+	+	- Does not represent natural etiology of disorder<br>- Limited to synucleinopathy	

					aspects of PD<br>- Does not capture multifaceted nature of disorder	
Tardive dyskinesia	Haloperidol decanoate	+	+	+	- Does not replicate complexity of the disorder	Induces abnormal motor behaviours including VCMs and cataplexy; Histopathophysiological changes and degeneration in substantia nigra; Responsive to treatments such as DA receptor antagonists and antioxidant agents
Essential tremor	Harmaline	+	+	+	- Variability in tremor type<br>- Unlikely to recapitulate chronic aspects of essential tremor	Induce both postural and action tremors; Induce neuronal loss in the cerebellum; Responsive to treatments such as beta blockers, L-DOPA, and DA receptor agonists
ASD	Genetic manipulation (i.e., selective breeding, transgenic)	+	+	+	- Some possess monogenic rather than polygenic forms of ASD<br>- Genetically un-identical strains between vendors<br>- Intra- and inter-laboratory phenotype variability	Behaviours comparable to symptoms observed in ASD patients (i.e., repetitive behaviours, social deficits); Pathophysiological changes such as ASD-related genetic mutations or altered monoamine systems; Responsive to ASD-related pharmacological intervention
	VPA exposure	+	+	+	- Behaviour influenced by timing of VPA exposure<br>- Models only ASD cases caused by exposure to drugs with HDAC inhibitory activity<br>- 1:1 male to female impact, unlike general ASD population	
Tourette syndrome	Pharmacological (i.e., apomorphine, serum antinuclear antibodies)	+	+	+	- Not unique to modelling Tourette syndrome<br>- Models only aspects of key tic phenomenology, but not complex neuropsychiatric abnormalities	Behavioural stereotypies such as motor tics, vocal tics, and/or hyperactivity; Pathophysiological changes such as basal ganglia and/or dopamine dysfunction; Responsive to antipsychotic drugs
OCD	5-CSRTT, prepulse	+	-	+	- Limited to compulsive-like behaviours	Emergence of observable

	inhibition				- Prepulse inhibition deficits are not specific to OCD	behaviours reminiscent of compulsive behaviour; Dysfunction in cortico-striatal-thalamo-cortical circuitry; Responsive to serotonin reuptake inhibitors and/or neuromodulation
	Genetic manipulation (i.e. transgenic)	+	+	+	- Lack of clear susceptibility genes<br>- Targeted mutations may induce comorbid dysfunctions	
	Pharmacological (i.e., quinpirole)	+	+	+	- Limited OCD behavioural repertoire exhibited (i.e., increased repetition)	
Pain	Sciatic nerve injury	+	+	+	- Induce non-pain sensory abnormalities<br>- Cannot differentiate between responses to pain and non-pain sensation; behaviours can be misinterpreted as responses to pain	Induces neuropathic pain with similar etiology to human disorder; Display mechanical allodynia, thermal hyperalgesia, and altered spontaneous motor activity; Responsive to analgesics and modulators of the endocannabinoid system
Spinal cord injury	Mechanical contusion	+	+	+	- Variability in the induced injury severity	Exhibit neurological deficits associated with spinal cord injury; Pathophysiological changes including increased inflammation, oxidative stress, and neuronal cell death; Responsive to replacement therapies and anti-inflammatory approaches
	Partial transection	+	+	+	- Variability in lesion pattern and resulting injury severity	
Traumatic brain injury	Fluid percussion injury	+	+	+	- Variability in injury outcome and severity<br>- Lack of control in region of injury	Broad neurological impairment in motor, sensory, cognitive, and emotional function; Induce primary neural injury with inflammation, changes in neurotransmitter signalling; Responsive to plasticity and anti-inflammatory based therapeutic approaches
	Weight drop	+	+	+	- Variability in injury outcome and severity<br>- Induces focal injury and does not capture diffuse underlying sources of injury	
Psychosis	Behavioural tests (i.e., prepulse inhibition, latent inhibition, hyperactivity)	+	-	+	- Inability to interpret motivation underlying behaviours<br>- Behaviours influenced by sex<br>- Prepulse inhibition deficits are not specific	Behaviours comparable to symptoms observed in patients with psychosis (i.e., hyperactivity, altered sensory-motor

					to psychosis	gating); Pathophysiological changes such as DAergic and glutamatergic dysfunction, neuroinflammation, and oxidative stress; Responsive to anti-psychotic drugs and therapies
	Developmental manipulations (i.e., maternal immune activation, methylazoxymethanol acetate, neonatal ventral hippocampal lesion)	+	+	+	- Maternal immune activation and neonatal ventral hippocampal lesion are laborious, time-consuming, and present risk of mortality<br>- Methylazoxymethanol acetate is toxic and carcinogenic	
	Pharmacological (i.e., ketamine, MK-801)	+	+	+	- Differing drug dosage and regimens lead to divergent results<br>- MK-801 induces acute psychosis rather than chronic<br>- Limited value in understanding etiopathology	
Sleep-Wake Disorders	Genetic manipulation (i.e. transgenic)	+	+	+	- High inter-animal variability in induced narcolepsy-like behaviours<br>- Rodent sleep patterns are not representative of normal human sleep patterns	Irregular sleep state patterns and cataplexy-like behaviours; Capture the progressive degeneration of orexin neurons similar to patients with narcolepsy; Responsive to orexin cell replacement therapy and orexin receptor agonist
Substance abuse/addictive disorders	Conditioned place preference	-	-	+	- Lack of animal-driven behaviour<br>- Not exclusive to drugs of abuse<br>- Lacks face and construct validity	Behaviours comparable to symptoms of substance abuse/addictive disorders (i.e., increased consumption, preference, and/or motivation for addictive substance or reward); Neurochemical and neuroanatomical substrates involved in drug-intake behaviour similar to those observed in humans (i.e., dysfunctional reward circuitry); Responsive to addiction-related interventions
	Drug self-administration	+	+	+	- Short lifespan of intravenous catheters for drug infusions<br>- Long and complex training sessions/ protocols	
	Intracranial self-stimulation	+	+	+	- Involves direct activation of reward circuits, bypassing input side of system<br>- Labour-intensive, requires surgery	
Tinnitus	Noise trauma-	+	+	+	- Does not capture non-	Induces tinnitus-like

	induced				cochlear damage related causes of tinnitus<br>- High variability in animals that display tinnitus-like behaviour	behaviours such as altered auditory startle responses through a natural etiology; Alongside primary cochlear damage, pathophysiological changes include neural firing patterns, increased neuroinflammation, and plasticity changes; Responsive to therapeutic approaches with GABA and NMDA modulators, neurotrophic factors, and antidepressants.
Trauma/ Stress related Disorders (PTSD)	Fear conditioning	+	+	+	- Physical stressors (i.e., electric foot shock) may result in physical injury<br>- Lacks ability to model delayed or chronic forms of PTSD<br>- No clear protocol distinction between fear conditioning and PTSD models	Induces biological and behavioural sequelae of PTSD such as long-lasting mood symptoms, history of stress exposure, altered HPA axis, and activation of amygdala and memory circuits; Responsive to anxiety and panic syndrome therapies

Abbreviations: 5-CSRTT: 5-choice serial reaction time task; 6-OHDA: 6-hydroxydopamine; ASD: autism spectrum disorder; Atx: ataxin-3; DA: dopamine;EPM: elevated plus maze; FST: forced swimming test; GABA: gamma-aminobutyric acid; HDAC: histone deacetylase; HPA: hypothalamic-pituitary-adrenal; L-DOPA: levodopa; NMDA: N-methyl-D-aspartate; OCD: obsessive-compulsive disorder; OF: open field; PD: Parkinson's disease;PTSD: post-traumatic stress disorder; PTZ: pentylenetetrazol; TST: tail suspension test; VCM: vacuous chewing movement; VPA: valproic acid

Supplementary Table 4. Summary of deep brain stimulation parameters employed in rodent studies

Rat Studies

Model	Amplitude				Frequency (Hz)				Pulse Width (µs)			
	Avg ± SDev	Range	Median	Mode	Avg ± SDev	Range	Median	Mode	Avg ± SDev	Range	Median	Mode
Anxiety Disorder	183.3 µA ± 75.3	100-300 µA	200 µA	200 µA	111.4 ± 14.6	100-130	100	100	162.9 ± 158.5	40-500	100	100
Dementia/ Cognition	155.1 µA ± 135.2	3-1500 µA	100 µA	100 µA	83.9 ± 44.5	1-130	100	100	113.7 ± 97.1	25-500	100	100
Depression	200.7 µA ± 108.8<br>2.3 V ± 0.3	50-500 µA<br>2-2.5 V	200 µA<br>2.5 V	100 µA<br>2.5 V	106.6 ± 44.4	5-150	130	130	111.4 ± 56.6	60-200000	90	90
Eating Disorder	159.3 µA ± 127.2<br>2 V	10-500 µA<br>2 V	150 µA<br>2 V	150 µA<br>N/A	122.0 ± 48.3	10-200	130	130	85.0 ± 44.0	60-100000	60	60
Epilepsy	254.9 µA ± 174.2<br>1 V	50-800 µA<br>1 V	200 µA<br>1 V	100 µA<br>N/A	109.1 ± 72.3	1-390	130	130	96.4 ± 53.6	60-1000	90	90
Movement Disorder	159.0 µA ± 160.8<br>1.6 V ± 1.0	1-1500 µA<br>0.5-3 V	100 µA<br>1.5 V	100 µA<br>1.5 V	110.0 ± 53.0	1-350	130	130	84.3 ± 58.2	0.5-1000	60	60
Neurodevelopmental Disorder	265.0 µA ± 165.0	150-500 µA	205 µA	150 µA	130.0 ± 23.5	100-160	130	130	76.3 ± 58.2	25-160	60	60
No pathology (healthy strains)	229.6 µA ± 178.6<br>5.1 V ± 6.7	3-3600<br>0.8-20 V	200<br>2.5 V	100<br>2.5 V	118.7 ± 91.4	6-1000	127	130	143.4 ± 119.3	50-9000	100	100
Obsessive-Compulsive Disorder	112.2 µA ± 124.6	3-500 µA	100 µA	100 µA	130	130	130	130	91.1 ± 42.6	60-100	60	60
Physical Injury	100.0 µA ± 103.0<br>2.1 V ± 0.8	20-250 µA<br>1-4 V	65 µA<br>2 V	N/A<br>2.5 V	84.9 ± 53.4	7.7-200	75	130	201.0 ± 174.7	20-500	141	20
Psychosis	175.0 µA ± 69.2	75-300 µA	150 µA	150 µA	109.4 ± 41.3	5-130	130	130	108.8 ± 37.2	90-200	100	100
Tinnitus	96.4 µA ± 30.4	50-150 µA	100 µA	100 µA	82.0 ± 40.3	10-100	100	100	44.3 ± 21.5	10-60	60	60
Trauma/Stressor Related Disorders	200.0 µA ± 141.4<br>2.6 V ± 0.2	100-300 µA<br>2.5-3 V	200 µA<br>2.5 V	N/A<br>2.5 V	140.0 ± 60.8	10-200	160	160	132.5 ± 47.2	90-120000	160	120
Substance Use Disorders	162.9 µA ± 88.8<br>2 V	20-500 µA<br>2 V	150 µA<br>2 V	150 µA<br>2 V	105.1 ± 54.4	8-160	130	130	119.2 ± 118.7	60-100000	95	60
**Overall**	**184.1 µA ± 150.1**<br>**2.7 V ± 3.2**	**1-3600 µA**<br>**0.5-20 V**	**150 µA**<br>**2 V**	**100 µA**<br>**2.5 V**	**109.8 ± 63.6**	**1-1000**	**130**	**130**	**109.9 ± 90.2**	**0.5-200000**	**90**	**60**

Mouse Studies

Model	Amplitude				Frequency (Hz)				Pulse Width (µs)			
	Avg ± SDev[#]	Range	Median	Mode	Avg ± SDev[#]	Range	Median	Mode	Avg ± SDev[#]	Range	Median	Mode
Anxiety Disorder	100 µA<br>3 V ± 1.4	100 µA<br>2-4 V	100 µA<br>3 V	N/A<br>N/A	84.0 ± 71.2	2-130	120	N/A	56.7± 45.1	10-100	60	N/A
Dementia/ Cognition	137.5 µA ± 110.9	50-1000000 µA	100 µA	100 µA	93.5 ± 47.5	10-130	115	130	91.7 ± 4.1	90-100	90	90
Depression	110.0 µA ± 22.4<br>2.5 V	100-40000 µA<br>2.5 V	100 µA<br>2.5 V	100 µA<br>N/A	130 ± 21.2	100-160	130	130	81.7 ± 17.2	60-100	90	90
Eating Disorder	108.3 µA ± 38.2	75-150 µA	100 µA	N/A	145.0 ± 21.2	130-160	145	N/A	75.0 ± 21.2	60-90	75	N/A

Epilepsy	100 µA	100 µA	100 µA	N/A	43.2 ± 56.8	1-130	12.5	N/A	96.7 ± 5.8	90-100	100	100
	2.0 V	2.0 V	2.0 V	N/A								
Movement Disorder	90.9 µA ± 63.7	15-200 µA	80 µA	100 µA	125 ± 7.1	120-130	125	N/A	83.0 ± 33.7	60-150	60	60
Neurodevelopmental Disorder	40.0 µA ± 14.1	30-50 µA	40 µA	N/A	130	130	130	130	60	60	60	60
No pathology (healthy strains)	91.4 µA ± 37.2	25-150 µA	90 µA	75 µA	139.4 ± 117.1	10-1000	130	130	142.2 ± 140.4	60-7780	95	60
	2.75 V ± 0.4	2.5-3 V	2.75 V	N/A								
Obsessive-Compulsive Disorder	300 µA	300 µA	300 µA	N/A	120.0	120.0	120.0	N/A	80	80	80	N/A
Physical Injury	150 µA	150 µA	150 µA	N/A	120.0	120.0	120.0	N/A	145.0 ± 77.8	90-200	145	N/A
	4.0 V	4.0 V	4.0 V	N/A								
Psychosis	100 µA	100 µA	100 µA	N/A	130.0	130.0	130.0	N/A	60	60	60	N/A
Sleep-wake Disorder	2.5 V ± 2.1	1-4	2.5	N/A	15.0	15.0	15.0	N/A	n/d	n/d	n/d	n/d
Substance Use Disorders	200.0 µA ± 141.4	100-300 µA	200 µA	N/A	88.3 ± 72.2	5-130	130	130	45.8 ± 51	0.3-90	46.5	90
**Overall**	**107.3 ± 66.0**	**15-1000000**	**100**	**100**	**106.4 ± 74.0**	**1-1000**	**130**	**130**	**89.9 ± 68.1**	**0.3-780**	**90**	**60**
	**2.8 V ± 1.1**	**1-4 V**	**2.5 V**	**N/A**								

#: Average (Avg) and standard deviation (SDev) calculated using values below 1000. N/A: unable to be calculated. n/d: not described.

Supplementary Table 5. Behavioural changes associated with deep brain stimulation of various targets in different disease models.

Model	Brain Target	Behavioural Outcome
Anxiety Disorder	nAcc, VS	- Enhanced fear extinction following extinction testing in standard operant chambers<br>- Core DBS during extinction training reduces fear in extinction retrieval during fear conditioning paradigm<br>- No effect in EPM
	LHA	- Reduced anxiety-like behaviour
	PAG	- HFS induced panic-related behaviours
	BNST	- No effect on anxiety-like behaviour
	Caudate nucleus	- Decreased conditioned and unconditioned anxiety
	IC	- Decreased conditioned anxiety
	vmPFC	- Anxiolytic-like effect when coupled with simultaneous exposure to enriched environment
Bladder function	PAG, PnO, PPTg	- Induced near complete inhibition of reflexive isovolumetric bladder contractions, PPTg- DBS was most effective
	LC	- Augmented reflexive isovolumetric bladder contractions
Dementia/ Cognition	EPN	- Attenuated cognitive and motor dysfunction in model of Huntington's disease
Depression	CPu	- No effect on depressive-like behaviours
	CC	- HFS induced anxiolytic-like effects and increased motivation for food
Eating disorder	VTA	- LFS decreased food intake, no effect on motor or anxiety-like behaviors
Epilepsy	Thalamus	- STN DBS induced anti-seizure effects<br>- Mediodorsal thalamic DBS had no effect on seizure development or activity
	Olfactory bulb	- Anti-seizure effects<br>- Improve memory performance
Healthy	PPTg, pontine reticular nucleus, LC	- Nearly completely inhibited reflexive isovolumetric bladder contractions, augmented in LC DBS
OCD	EPN	- No effect on prepulse inhibition
Physical injury	Thalamus	- Decreased pain score and response in rat models of neuropathic pain
	PPTg	- Forced locomotion, associated with stress responses in rat models of SCI<br>- 50 Hz and 2 V DBS reverted bladder voiding efficiency in rat models of TBI
	Cuneiform nucleus	- Improved locomotion and long-term recovery of motor function in highly paraparetic rats with severe SCI
Psychosis	NAcc	- Antipsychotic-like effects
Substance use	LHA	- HFS blocks morphine-induced CPP in male Wistar rats<br>- No effect on consumption or preference for water or natural rewards<br>- Anxiolytic- and anti anhedonia-like effects
	Amygdala, hippocampus	- Reduced acquisition, expression, and reinstatement of drug-seeking
	PFC	- Attenuated reinstatement of cocaine seeking

	LHb	- Reduced sucrose self-administration
	OFC	- HFS decreased morphine preference and reinstatement, LFS not effective
	SN	- HFS induced extinction of methamphetamine-induced CPP and prevented drug-primed reinstatement, LFS impaired extinction
Tinnitus	Inferior colliculus	- Alleviated tinnitus-like behaviour
	Cerebellar Dentate Nucleus	- No effect on tinnitus-like behaviour

Abbreviations: BNST: bed nucleus of the stria terminalis; CC: cingulate cortex; CPP: conditioned place-preference; CPu: caudate putamen; DBS: deep brain stimulation; EPM: elevated plus maze; EPN: entopeduncular nucleus; HFS: high frequency stimulation; IC: internal capsule; LC: locus coeruleus; LFS: low frequency stimulation; LHA: lateral hypothalamus; LHb: lateral habenula; NAcc: nucleus accumbens; OCD: Obsessive-compulsive disorder; OFC: orbitofrontal cortex; PAG: periaqueductal grey; PFC: prefrontal cortex; PnO: pontine reticular nucleus; PPTg: pedunculopontine tegmental nucleus; SCI: spinal cord injury; SN: substantia nigra; STN: subthalamic nucleus; TBI: traumatic brain injury; vmPFC: ventromedial prefrontal cortex; VS: ventral striatum; VTA: ventral tegmental area
