# Supplementary material for: Systematic review of rodent studies of deep brain stimulation for the treatment of neurological, developmental and neuropsychiatric disorders
Source: Transl Psychiatry. 2024 Apr 11;14:186. doi: 10.1038/s41398-023-02727-5 (PMC11009311; doi:10.1038/s41398-023-02727-5)
Supplement: Supplementary file 3 — Supplementary References [file 41398_2023_2727_MOESM3_ESM.pdf]

SUPPLEMENTARY REFERENCES

Adams, W. K., Vonder Haar, C., Tremblay, M., Cocker, P. J., Silveira, M. M., Kaur, S., Baunez, C., & Winstanley, C. A. (2017). Deep-Brain Stimulation of the Subthalamic Nucleus Selectively Decreases Risky Choice in Risk-Preferring Rats. *eNeuro*, *4*(4). https://doi.org/10.1523/ENEURO.0094-17.2017

Agnesi, F., Blaha, C. D., Lin, J., & Lee, K. H. (2010). Local glutamate release in the rat ventral lateral thalamus evoked by high-frequency stimulation. *Journal of Neural Engineering*, *7*(2), 26009.

Ahsan, S. F., Luo, H., Zhang, J., Kim, E., & Xu, Y. (2018). An animal model of deep brain stimulation for treating tinnitus: A proof of concept study. *The Laryngoscope*, *128*(5), 1213–1222.

Akita, H., Honda, Y., Ogata, M., Noda, K., & Saji, M. (2010). Activation of the NMDA receptor involved in the alleviating after-effect of repeated stimulation of the subthalamic nucleus on motor deficits in hemiparkinsonian rats. *Brain Research*, *1306*, 159–167.

Alam, M., Capelle, H.-H., Schwabe, K., & Krauss, J. K. (2014). Effect of deep brain stimulation on levodopa-induced dyskinesias and striatal oscillatory local field potentials in a rat model of Parkinson's disease. *Brain Stimulation*, *7*(1), 13–20.

Alam, M., Heissler, H. E., Schwabe, K., & Krauss, J. K. (2012). Deep brain stimulation of the pedunculopontine tegmental nucleus modulates neuronal hyperactivity and enhanced beta oscillatory activity of the subthalamic nucleus in the rat 6-hydroxydopamine model. *Experimental Neurology*, *233*(1), 233–242.

Aldehri, M., Temel, Y., Jahanshahi, A., & Hescham, S. (2019). Fornix deep brain stimulation induces reduction of hippocampal synaptophysin levels. *Journal of Chemical Neuroanatomy*, *96*, 34–40.

Aleksandrova, L. R., Creed, M. C., Fletcher, P. J., Lobo, D. S. S., Hamani, C., & Nobrega, J. N. (2013). Deep brain stimulation of the subthalamic nucleus increases premature responding in a rat gambling task. *Behavioural Brain Research*, *245*, 76–82.

Almasabi, F., van Zwieten, G., Alosaimi, F., Smit, J. V., Temel, Y., Janssen, M. L. F., & Jahanshahi, A. (2022). The Effect of Noise Trauma and Deep Brain Stimulation of the Medial Geniculate Body on Tissue Activity in the Auditory Pathway. *Brain Sciences*, *12*(8). https://doi.org/10.3390/brainsci12081099

Alosaimi, F., Temel, Y., Hescham, S., Witzig, V. S., Almasabi, F., Tan, S. K. H., & Jahanshahi, A. (2022). High-frequency stimulation of the subthalamic nucleus induces a sustained inhibition of serotonergic system via loss of cell phenotype. *Scientific Reports*, *12*(1), 14011.

Alpaugh, M., Saint-Pierre, M., Dubois, M., Aubé, B., Arsenault, D., Kriz, J., Cicchetti, A., & Cicchetti, F. (2019). A novel wireless brain stimulation device for long-term use in freely moving mice. *Scientific Reports*, *9*(1), 6444.

Amoozegar, S., Pooyan, M., & Roghani, M. (2022). Identification of effective features of LFP signal for making closed-loop deep brain stimulation in parkinsonian rats. *Medical & Biological Engineering & Computing*, *60*(1), 135–149.

Amorim, B. O., Covolan, L., Ferreira, E., Brito, J. G., Nunes, D. P., de Morais, D. G., Nobrega, J. N., Rodrigues, A. M., deAlmeida, A. C. G., & Hamani, C. (2015). Deep brain stimulation induces antiapoptotic and anti-inflammatory effects in epileptic rats. *Journal of Neuroinflammation*, *12*, 162.

Anderson, C. J., Sheppard, D. T., Huynh, R., Anderson, D. N., Polar, C. A., & Dorval, A. D. (2015). Subthalamic deep brain stimulation reduces pathological information transmission to the thalamus in a rat model of parkinsonism. *Frontiers in Neural Circuits*, *9*, 31.

Anderson, C., Sheppard, D., & Dorval, A. D. (2020). Parkinsonism and subthalamic deep brain stimulation dysregulate behavioral motivation in a rodent model. *Brain Research*, *1736*, 146776.

Angelov, S. D., Dietrich, C., Krauss, J. K., & Schwabe, K. (2014). Effect of deep brain stimulation in rats selectively bred for reduced prepulse inhibition. *Brain Stimulation*, *7*(4), 595–602.

Apetz, N., Kordys, E., Simon, M., Mang, B., Aswendt, M., Wiedermann, D., Neumaier, B., Drzezga, A., Timmermann, L., & Endepols, H. (2019). Effects of subthalamic deep brain stimulation on striatal

metabolic connectivity in a rat hemiparkinsonian model. *Disease Models & Mechanisms*, *12*(5). https://doi.org/10.1242/dmm.039065

Arrieta-Cruz, I., Pavlides, C., & Pasinetti, G. M. (2010). Deep Brain Stimulation In Midline Thalamic Region Facilitates Synaptic Transmission And Shortterm Memory In A Mouse Model Of Alzheimer's Disease. *Translational Neuroscience*, *1*(3), 188–194.

Ashouri Vajari, D., Ramanathan, C., Tong, Y., Stieglitz, T., Coenen, V. A., & Döbrössy, M. D. (2020). Medial forebrain bundle DBS differentially modulates dopamine release in the nucleus accumbens in a rodent model of depression. *Experimental Neurology*, *327*, 113224.

Bachmann, L. C., Matis, A., Lindau, N. T., Felder, P., Gullo, M., & Schwab, M. E. (2013). Deep brain stimulation of the midbrain locomotor region improves paretic hindlimb function after spinal cord injury in rats. *Science Translational Medicine*, *5*(208), 208ra146.

Badstuebner, K., Gimsa, U., Weber, I., Tuchscherer, A., & Gimsa, J. (2017). Deep Brain Stimulation of Hemiparkinsonian Rats with Unipolar and Bipolar Electrodes for up to 6 Weeks: Behavioral Testing of Freely Moving Animals. *Parkinson's Disease*, *2017*, 5693589.

Bae, S., Lim, H.-K., Jeong, Y., Kim, S.-G., Park, S.-M., Shon, Y.-M., & Suh, M. (2022). Deep brain stimulation of the anterior nuclei of the thalamus can alleviate seizure severity and induce hippocampal GABAergic neuronal changes in a pilocarpine-induced epileptic mouse brain. *Cerebral Cortex* , *32*(24), 5530–5543.

Bambico, F. R., Bregman, T., Diwan, M., Li, J., Darvish-Ghane, S., Li, Z., Laver, B., Amorim, B. O., Covolan, L., Nobrega, J. N., & Hamani, C. (2015). Neuroplasticity-dependent and -independent mechanisms of chronic deep brain stimulation in stressed rats. *Translational Psychiatry*, *5*(11), e674.

Bambico, F. R., Comai, S., Diwan, M., Hasan, S. M. N., Conway, J. D., Darvish-Ghane, S., Hamani, C., Gobbi, G., & Nobrega, J. N. (2018). High frequency stimulation of the anterior vermis modulates behavioural response to chronic stress: involvement of the prefrontal cortex and dorsal raphe? *Neurobiology of Disease*, *116*, 166–178.

Batra, V., Guerin, G. F., Goeders, N. E., & Wilden, J. A. (2016). A General Method for Evaluating Deep Brain Stimulation Effects on Intravenous Methamphetamine Self-Administration. *Journal of Visualized Experiments: JoVE*, *107*, e53266.

Batra, V., Tran, T. L. N., Caputo, J., Guerin, G. F., Goeders, N. E., & Wilden, J. (2017). Intermittent bilateral deep brain stimulation of the nucleus accumbens shell reduces intravenous methamphetamine intake and seeking in Wistar rats. *Journal of Neurosurgery*, *126*(4), 1339–1350.

Bazzu, G., Serra, P. A., Hamelink, R., Feenstra, M. G. P., Willuhn, I., & Denys, D. (2019). Monitoring deep brain stimulation by measuring regional brain oxygen responses in freely moving mice. *Journal of Neuroscience Methods*, *317*, 20–28.

Bekar, L., Libionka, W., Tian, G.-F., Xu, Q., Torres, A., Wang, X., Lovatt, D., Williams, E., Takano, T., Schnermann, J., Bakos, R., & Nedergaard, M. (2008). Adenosine is crucial for deep brain stimulation-mediated attenuation of tremor. *Nature Medicine*, *14*(1), 75–80.

Bezchlibnyk, Y. B., Stone, S. S. D., Hamani, C., & Lozano, A. M. (2017). High frequency stimulation of the infralimbic cortex induces morphological changes in rat hippocampal neurons. *Brain Stimulation*, *10*(2), 315–323.

Bhaskar, Y., Lim, L. W., & Mitra, R. (2018). Enriched Environment Facilitates Anxiolytic Efficacy Driven by Deep-Brain Stimulation of Medial Prefrontal Cortex. *Frontiers in Behavioral Neuroscience*, *12*, 204.

Bikovsky, L., Hadar, R., Soto-Montenegro, M. L., Klein, J., Weiner, I., Desco, M., Pascau, J., Winter, C., & Hamani, C. (2016). Deep brain stimulation improves behavior and modulates neural circuits in a rodent model of schizophrenia. *Experimental Neurology*, *283*(Pt A), 142–150.

Blik, V. (2015). Electric stimulation of the tuberomamillary nucleus affects epileptic activity and sleep-wake cycle in a genetic absence epilepsy model. *Epilepsy Research*, *109*, 119–125.

Bonizzato, M., James, N. D., Pidpruzhnykova, G., Pavlova, N., Shkorbatova, P., Baud, L., Martinez-Gonzalez, C., Squair, J. W., DiGiovanna, J., Barraud, Q., Micera, S., & Courtine, G. (2021). Multi-pronged neuromodulation intervention engages the residual motor circuitry to facilitate walking

in a rat model of spinal cord injury. *Nature Communications*, *12*(1), 1925.
Bregman, T., Nona, C., Volle, J., Diwan, M., Raymond, R., Fletcher, P. J., Nobrega, J. N., & Hamani, C. (2018). Deep brain stimulation induces antidepressant-like effects in serotonin transporter knockout mice. *Brain Stimulation*, *11*(2), 423–425.
Bregman, T., Reznikov, R., Diwan, M., Raymond, R., Butson, C. R., Nobrega, J. N., & Hamani, C. (2015). Antidepressant-like Effects of Medial Forebrain Bundle Deep Brain Stimulation in Rats are not Associated With Accumbens Dopamine Release. *Brain Stimulation*, *8*(4), 708–713.
Brown, A. R., Antle, M. C., Hu, B., & Teskey, G. C. (2011). High frequency stimulation of the subthalamic nucleus acutely rescues motor deficits and neocortical movement representations following 6-hydroxydopamine administration in rats. *Experimental Neurology*, *231*(1), 82–90.
Bruchim-Samuel, M., Lax, E., Gazit, T., Friedman, A., Ahdoot, H., Bairachnaya, M., Pinhasov, A., & Yadid, G. (2016). Electrical stimulation of the vmPFC serves as a remote control to affect VTA activity and improve depressive-like behavior. *Experimental Neurology*, *283*(Pt A), 255–263.
Bühning, F., Miguel Telega, L., Tong, Y., Pereira, J., Coenen, V. A., & Döbrössy, M. D. (2022). Electrophysiological and molecular effects of bilateral deep brain stimulation of the medial forebrain bundle in a rodent model of depression. *Experimental Neurology*, *355*, 114122.
Calleja-Castillo, J. M., De La Cruz-Aguilera, D. L., Manjarrez, J., Velasco-Velázquez, M. A., Morales-Espinoza, G., Moreno-Aguilar, J., Hernández, M. E., Aguirre-Cruz, L., & Pavón, L. (2013). Chronic deep brain stimulation of the hypothalamic nucleus in wistar rats alters circulatory levels of corticosterone and proinflammatory cytokines. *Clinical & Developmental Immunology*, *2013*, 698634.
Campos, A. C. P., Kikuchi, D. S., Paschoa, A. F. N., Kuroki, M. A., Fonoff, E. T., Hamani, C., Pagano, R. L., & Hernandes, M. S. (2020). Unraveling the Role of Astrocytes in Subthalamic Nucleus Deep Brain Stimulation in a Parkinson's Disease Rat Model. *Cellular and Molecular Neurobiology*, *40*(6), 939–954.
Capozzo, A., Vitale, F., Mattei, C., Mazzone, P., & Scarnati, E. (2014). Continuous stimulation of the pedunculopontine tegmental nucleus at 40 Hz affects preparative and executive control in a delayed sensorimotor task and reduces rotational movements induced by apomorphine in the 6-OHDA parkinsonian rat. *Behavioural Brain Research*, *271*, 333–342.
Casquero-Veiga, M., Bueno-Fernandez, C., Romero-Miguel, D., Lamanna-Rama, N., Nacher, J., Desco, M., & Soto-Montenegro, M. L. (2021). Exploratory study of the long-term footprint of deep brain stimulation on brain metabolism and neuroplasticity in an animal model of obesity. *Scientific Reports*, *11*(1), 5580.
Casquero-Veiga, M., García-García, D., Desco, M., & Soto-Montenegro, M. L. (2018). Understanding Deep Brain Stimulation: Metabolic Consequences of the Electrode Insertional Effect. *BioMed Research International*, *2018*, 8560232.
Casquero-Veiga, M., García-García, D., Pascau, J., Desco, M., & Soto-Montenegro, M. L. (2018). Stimulating the nucleus accumbens in obesity: A positron emission tomography study after deep brain stimulation in a rodent model. *PloS One*, *13*(9), e0204740.
Casquero-Veiga, M., Hadar, R., Pascau, J., Winter, C., Desco, M., & Soto-Montenegro, M. L. (2016). Response to Deep Brain Stimulation in Three Brain Targets with Implications in Mental Disorders: A PET Study in Rats. *PloS One*, *11*(12), e0168689.
Cassar, I. R., & Grill, W. M. (2022). The cortical evoked potential corresponds with deep brain stimulation efficacy in rats. *Journal of Neurophysiology*, *127*(5), 1253–1268.
Cervera-Ferri, A., Teruel-Martí, V., Barceló-Molina, M., Martínez-Ricós, J., Luque-García, A., Martínez-Bellver, S., & Adell, A. (2016). Characterization of oscillatory changes in hippocampus and amygdala after deep brain stimulation of the infralimbic prefrontal cortex. *Physiological Reports*, *4*(14). https://doi.org/10.14814/phy2.12854
Chakravarty, M. M., Hamani, C., Martinez-Canabal, A., Ellegood, J., Laliberté, C., Nobrega, J. N., Sankar, T., Lozano, A. M., Frankland, P. W., & Lerch, J. P. (2016). Deep brain stimulation of the ventromedial prefrontal cortex causes reorganization of neuronal processes and vasculature. *NeuroImage*, *125*,

422–427.
Chamaa, F., Darwish, B., Nahas, Z., Al-Chaer, E. D., Saadé, N. E., & Abou-Kheir, W. (2021). Long-term stimulation of the anteromedial thalamus increases hippocampal neurogenesis and spatial reference memory in adult rats. *Behavioural Brain Research*, *402*, 113114.
Chamaa, F., Sweidan, W., Nahas, Z., Saade, N., & Abou-Kheir, W. (2016). Thalamic Stimulation in Awake Rats Induces Neurogenesis in the Hippocampal Formation. *Brain Stimulation*, *9*(1), 101–108.
Chang, A. D., Berges, V. A., Chung, S. J., Fridman, G. Y., Baraban, J. M., & Reti, I. M. (2016). High-Frequency Stimulation at the Subthalamic Nucleus Suppresses Excessive Self-Grooming in Autism-Like Mouse Models. *Neuropsychopharmacology: Official Publication of the American College of Neuropsychopharmacology*, *41*(7), 1813–1821.
Chang, H., Gao, C., Sun, K., Xiao, L., Li, X., Jiang, S., Zhu, C., Sun, T., Jin, Z., & Wang, F. (2020). Continuous High Frequency Deep Brain Stimulation of the Rat Anterior Insula Attenuates the Relapse Post Withdrawal and Strengthens the Extinction of Morphine Seeking. *Frontiers in Psychiatry / Frontiers Research Foundation*, *11*, 577155.
Chang, J.-Y., Shi, L.-H., Luo, F., & Woodward, D. J. (2003). High frequency stimulation of the subthalamic nucleus improves treadmill locomotion in unilateral 6-hydroxydopamine lesioned rats. *Brain Research*, *983*(1-2), 174–184.
Chan, H. H., Wathen, C. A., Mathews, N. D., Hogue, O., Modic, J. P., Kundalia, R., Wyant, C., Park, H.-J., Najm, I. M., Trapp, B. D., Machado, A. G., & Baker, K. B. (2018). Lateral cerebellar nucleus stimulation promotes motor recovery and suppresses neuroinflammation in a fluid percussion injury rodent model. *Brain Stimulation*, *11*(6), 1356–1367.
Chen, S.-C., Chu, P.-Y., Hsieh, T.-H., Li, Y.-T., & Peng, C.-W. (2017). Feasibility of deep brain stimulation for controlling the lower urinary tract functions: An animal study. *Clinical Neurophysiology: Official Journal of the International Federation of Clinical Neurophysiology*, *128*(12), 2438–2449.
Chen, Y.-C., Shi, L., Zhu, G.-Y., Wang, X., Liu, D.-F., Liu, Y.-Y., Jiang, Y., Zhang, X., & Zhang, J.-G. (2017). Effects of anterior thalamic nuclei deep brain stimulation on neurogenesis in epileptic and healthy rats. *Brain Research*, *1672*, 65–72.
Chen, Y.-C., Zhu, G.-Y., Wang, X., Shi, L., Du, T.-T., Liu, D.-F., Liu, Y.-Y., Jiang, Y., Zhang, X., & Zhang, J.-G. (2017). Anterior thalamic nuclei deep brain stimulation reduces disruption of the blood-brain barrier, albumin extravasation, inflammation and apoptosis in kainic acid-induced epileptic rats. *Neurological Research*, *39*(12), 1103–1113.
Chen, Y.-C., Zhu, G.-Y., Wang, X., Shi, L., Jiang, Y., Zhang, X., & Zhang, J.-G. (2017). Deep brain stimulation of the anterior nucleus of the thalamus reverses the gene expression of cytokines and their receptors as well as neuronal degeneration in epileptic rats. *Brain Research*, *1657*, 304–311.
Chen, Y., Zhu, G., Liu, D., Zhang, X., Liu, Y., Yuan, T., Du, T., & Zhang, J. (2020). Subthalamic nucleus deep brain stimulation suppresses neuroinflammation by Fractalkine pathway in Parkinson's disease rat model. *Brain, Behavior, and Immunity*, *90*, 16–25.
Collins-Praino, L. E., Paul, N. E., Ledgard, F., Podurgiel, S. J., Kovner, R., Baqi, Y., Müller, C. E., Senatus, P. B., & Salamone, J. D. (2013). Deep brain stimulation of the subthalamic nucleus reverses oral tremor in pharmacological models of parkinsonism: interaction with the effects of adenosine A2A antagonism. *The European Journal of Neuroscience*, *38*(1), 2183–2191.
Cordon, I., Nicolás, M. J., Arrieta, S., Alegre, M., Artieda, J., & Valencia, M. (2018). Theta-phase closed-loop stimulation induces motor paradoxical responses in the rat model of Parkinson disease. *Brain Stimulation*, *11*(1), 231–238.
Covolan, L., de Almeida, A.-C. G., Amorim, B., Cavarsan, C., Miranda, M. F., Aarão, M. C., Madureira, A. P., Rodrigues, A. M., Nobrega, J. N., Mello, L. E., & Hamani, C. (2014). Effects of anterior thalamic nucleus deep brain stimulation in chronic epileptic rats. *PloS One*, *9*(6), e97618.
Creed, M. C., Hamani, C., Bridgman, A., Fletcher, P. J., & Nobrega, J. N. (2012). Contribution of decreased serotonin release to the antidyskinetic effects of deep brain stimulation in a rodent model of tardive dyskinesia: comparison of the subthalamic and entopeduncular nuclei. *The Journal of Neuroscience:*

The Official Journal of the Society for Neuroscience, *32*(28), 9574–9581.
Creed, M. C., Hamani, C., & Nobrega, J. N. (2012). Early gene mapping after deep brain stimulation in a rat model of tardive dyskinesia: comparison with transient local inactivation. *European Neuropsychopharmacology: The Journal of the European College of Neuropsychopharmacology*, *22*(7), 506–517.
Creed, M. C., Hamani, C., & Nobrega, J. N. (2013). Effects of repeated deep brain stimulation on depressive- and anxiety-like behavior in rats: comparing entopeduncular and subthalamic nuclei. *Brain Stimulation*, *6*(4), 506–514.
Creed, M., Hamani, C., & Nobrega, J. N. (2011). Deep brain stimulation of the subthalamic or entopeduncular nucleus attenuates vacuous chewing movements in a rodent model of tardive dyskinesia. *European Neuropsychopharmacology: The Journal of the European College of Neuropsychopharmacology*, *21*(5), 393–400.
Creed, M., Pascoli, V. J., & Lüscher, C. (2015). Addiction therapy. Refining deep brain stimulation to emulate optogenetic treatment of synaptic pathology. *Science*, *347*(6222), 659–664.
Dandekar, M. P., Luse, D., Hoffmann, C., Cotton, P., Peery, T., Ruiz, C., Hussey, C., Giridharan, V. V., Soares, J. C., Quevedo, J., & Fenoy, A. J. (2017). Increased dopamine receptor expression and anti-depressant response following deep brain stimulation of the medial forebrain bundle. *Journal of Affective Disorders*, *217*, 80–88.
Dandekar, M. P., Saxena, A., Scaini, G., Shin, J. H., Migut, A., Giridharan, V. V., Zhou, Y., Barichello, T., Soares, J. C., Quevedo, J., & Fenoy, A. J. (2019). Medial Forebrain Bundle Deep Brain Stimulation Reverses Anhedonic-Like Behavior in a Chronic Model of Depression: Importance of BDNF and Inflammatory Cytokines. *Molecular Neurobiology*, *56*(6), 4364–4380.
da Silva, J. C., Amorim, H., Scorza, F. A., Cavalheiro, E. A., & Cukiert, A. (2013). Brain electrical activity after acute hippocampal stimulation in awake rats. *Neuromodulation: Journal of the International Neuromodulation Society*, *16*(2), 100–104.
da Silva, J. C., Scorza, F. A., Nejm, M. B., Cavalheiro, E. A., & Cukiert, A. (2014). c-FOS expression after hippocampal deep brain stimulation in normal rats. *Neuromodulation: Journal of the International Neuromodulation Society*, *17*(3), 213–217; discussion 216–217.
Degoulet, M., Tiran-Cappello, A., Combrisson, E., Baunez, C., & Pelloux, Y. (2021). Subthalamic low-frequency oscillations predict vulnerability to cocaine addiction. *Proceedings of the National Academy of Sciences of the United States of America*, *118*(14). https://doi.org/10.1073/pnas.2024121118
Dejean, C., Hyland, B., & Arbuthnott, G. (2009). Cortical effects of subthalamic stimulation correlate with behavioral recovery from dopamine antagonist induced akinesia. *Cerebral Cortex* , *19*(5), 1055–1063.
Dela Cruz, J. A. D., Hescham, S., Adriaanse, B., Campos, F. L., Steinbusch, H. W. M., Rutten, B. P. F., Temel, Y., & Jahanshahi, A. (2015). Increased number of TH-immunoreactive cells in the ventral tegmental area after deep brain stimulation of the anterior nucleus of the thalamus. *Brain Structure & Function*, *220*(5), 3061–3066.
Desbonnet, L., Temel, Y., Visser-Vandewalle, V., Blokland, A., Hornikx, V., & Steinbusch, H. W. M. (2004). Premature responding following bilateral stimulation of the rat subthalamic nucleus is amplitude and frequency dependent. *Brain Research*, *1008*(2), 198–204.
Diepenbroek, C., van der Plasse, G., Eggels, L., Rijnsburger, M., Feenstra, M. G. P., Kalsbeek, A., Denys, D., Fliers, E., Serlie, M. J., & la Fleur, S. E. (2013). Alterations in blood glucose and plasma glucagon concentrations during deep brain stimulation in the shell region of the nucleus accumbens in rats. *Frontiers in Neuroscience*, *7*, 226.
Do-Monte, F. H., Rodriguez-Romaguera, J., Rosas-Vidal, L. E., & Quirk, G. J. (2013). Deep brain stimulation of the ventral striatum increases BDNF in the fear extinction circuit. *Frontiers in Behavioral Neuroscience*, *7*, 102.
Dong, X., Ye, W., Tang, Y., Wang, J., Zhong, L., Xiong, J., Liu, H., Lu, G., & Feng, Z. (2021). Wakefulness-Promoting Effects of Lateral Hypothalamic Area-Deep Brain Stimulation in Traumatic

Brain Injury-Induced Comatose Rats: Upregulation of α1-Adrenoceptor Subtypes and Downregulation of Gamma-Aminobutyric Acid β Receptor Expression Via the Orexins Pathway. *World Neurosurgery*, *152*, e321–e331.
Dorval, A. D., & Grill, W. M. (2014). Deep brain stimulation of the subthalamic nucleus reestablishes neuronal information transmission in the 6-OHDA rat model of parkinsonism. *Journal of Neurophysiology*, *111*(10), 1949–1959.
Doucette, W. T., Khokhar, J. Y., & Green, A. I. (2015). Nucleus accumbens deep brain stimulation in a rat model of binge eating. *Translational Psychiatry*, *5*(12), e695.
Dournes, C., Beeské, S., Belzung, C., & Griebel, G. (2013). Deep brain stimulation in treatment-resistant depression in mice: comparison with the CRF1 antagonist, SSR125543. *Progress in Neuro-Psychopharmacology & Biological Psychiatry*, *40*, 213–220.
Du, T.-T., Chen, Y.-C., Lu, Y.-Q., Meng, F.-G., Yang, H., & Zhang, J.-G. (2018). Subthalamic nucleus deep brain stimulation protects neurons by activating autophagy via PP2A inactivation in a rat model of Parkinson's disease. *Experimental Neurology*, *306*, 232–242.
Edemann-Callesen, H., Voget, M., Empl, L., Vogel, M., Wieske, F., Rummel, J., Heinz, A., Mathé, A. A., Hadar, R., & Winter, C. (2015). Medial Forebrain Bundle Deep Brain Stimulation has Symptom-specific Anti-depressant Effects in Rats and as Opposed to Ventromedial Prefrontal Cortex Stimulation Interacts With the Reward System. *Brain Stimulation*, *8*(4), 714–723.
Elle, T., Alam, M., Voigt, C., Krauss, J. K., John, N., & Schwabe, K. (2020). Deep brain stimulation of the thalamic centromedian-parafascicular nucleus improves behavioural and neuronal traits in a rat model of Tourette. *Behavioural Brain Research*, *378*, 112251.
Encinas, J. M., Hamani, C., Lozano, A. M., & Enikolopov, G. (2011). Neurogenic hippocampal targets of deep brain stimulation. *The Journal of Comparative Neurology*, *519*(1), 6–20.
Engelhardt, K.-A., Marchetta, P., Schwarting, R. K. W., & Melo-Thomas, L. (2018). Haloperidol-induced catalepsy is ameliorated by deep brain stimulation of the inferior colliculus. *Scientific Reports*, *8*(1), 2216.
Etiévant, A., Oosterhof, C., Bétry, C., Abrial, E., Novo-Perez, M., Rovera, R., Scarna, H., Devader, C., Mazella, J., Wegener, G., Sánchez, C., Dkhissi-Benyahya, O., Gronfier, C., Coizet, V., Beaulieu, J. M., Blier, P., Lucas, G., & Haddjeri, N. (2015). Astroglial Control of the Antidepressant-Like Effects of Prefrontal Cortex Deep Brain Stimulation. *EBioMedicine*, *2*(8), 898–908.
Ewing, S. G., & Grace, A. A. (2013a). Deep brain stimulation of the ventral hippocampus restores deficits in processing of auditory evoked potentials in a rodent developmental disruption model of schizophrenia. *Schizophrenia Research*, *143*(2-3), 377–383.
Ewing, S. G., & Grace, A. A. (2013b). Long-term high frequency deep brain stimulation of the nucleus accumbens drives time-dependent changes in functional connectivity in the rodent limbic system. *Brain Stimulation*, *6*(3), 274–285.
Faggiani, E., Delaville, C., & Benazzouz, A. (2015). The combined depletion of monoamines alters the effectiveness of subthalamic deep brain stimulation. *Neurobiology of Disease*, *82*, 342–348.
Fakhrieh-Asl, G., Sadr, S. S., Karimian, S. M., & Riahi, E. (2020). Deep brain stimulation of the orbitofrontal cortex prevents the development and reinstatement of morphine place preference. *Addiction Biology*, *25*(4), e12780.
Falowski, S. M., Sharan, A., Reyes, B. A. S., Sikkema, C., Szot, P., & Van Bockstaele, E. J. (2011). An evaluation of neuroplasticity and behavior after deep brain stimulation of the nucleus accumbens in an animal model of depression. *Neurosurgery*, *69*(6), 1281–1290.
Fang, X., Sugiyama, K., Akamine, S., & Namba, H. (2006). Improvements in motor behavioral tests during deep brain stimulation of the subthalamic nucleus in rats with different degrees of unilateral parkinsonism. *Brain Research*, *1120*(1), 202–210.
Fang, X., Sugiyama, K., Akamine, S., Sun, W., & Namba, H. (2010). The different performance among motor tasks during the increasing current intensity of deep brain stimulation of the subthalamic nucleus in rats with different degrees of the unilateral striatal lesion. *Neuroscience Letters*, *480*(1), 64–68.

Fattahi, M., Ashabi, G., Karimian, S. M., & Riahi, E. (2019). Preventing morphine reinforcement with high-frequency deep brain stimulation of the lateral hypothalamic area. *Addiction Biology*, *24*(4), 685–695.

Fauser, M., Ricken, M., Markert, F., Weis, N., Schmitt, O., Gimsa, J., Winter, C., Badstübner-Meeske, K., & Storch, A. (2021). Subthalamic nucleus deep brain stimulation induces sustained neurorestoration in the mesolimbic dopaminergic system in a Parkinson's disease model. *Neurobiology of Disease*, *156*, 105404.

Faust, K., Vajkoczy, P., Xi, B., & Harnack, D. (2021). The Effects of Deep Brain Stimulation of the Subthalamic Nucleus on Vascular Endothelial Growth Factor, Brain-Derived Neurotrophic Factor, and Glial Cell Line-Derived Neurotrophic Factor in a Rat Model of Parkinson's Disease. *Stereotactic and Functional Neurosurgery*, *99*(3), 256–266.

Feng, Z., Wang, Z., Guo, Z., Zhou, W., Cai, Z., & Durand, D. M. (2017). High frequency stimulation of afferent fibers generates asynchronous firing in the downstream neurons in hippocampus through partial block of axonal conduction. *Brain Research*, *1661*, 67–78.

Feng, Z., Yu, Y., Guo, Z., Cao, J., & Durand, D. M. (2014). High frequency stimulation extends the refractory period and generates axonal block in the rat hippocampus. *Brain Stimulation*, *7*(5), 680–689.

Fernández-Cabrera, M. R., Selvas, A., Miguéns, M., Higuera-Matas, A., Vale-Martínez, A., Ambrosio, E., Martí-Nicolovius, M., & Guillazo-Blanch, G. (2017). Parafascicular thalamic nucleus deep brain stimulation decreases NMDA receptor GluN1 subunit gene expression in the prefrontal cortex. *Neuroscience*, *348*, 73–82.

Ferreira, E. S., Vieira, L. G., Moraes, D. M., Amorim, B. O., Malheiros, J. M., Hamani, C., & Covolan, L. (2018). Long-Term Effects of Anterior Thalamic Nucleus Deep Brain Stimulation on Spatial Learning in the Pilocarpine Model of Temporal Lobe Epilepsy. *Neuromodulation: Journal of the International Neuromodulation Society*, *21*(2), 160–167.

Fischer, D. L., Collier, T. J., Cole-Strauss, A., Wohlgenant, S. L., Lipton, J. W., Steece-Collier, K., Manfredsson, F. P., Kemp, C. J., & Sortwell, C. E. (2015). High-Frequency Stimulation of the Rat Entopeduncular Nucleus Does Not Provide Functional or Morphological Neuroprotection from 6-Hydroxydopamine. *PloS One*, *10*(7), e0133957.

Fischer, D. L., Kemp, C. J., Cole-Strauss, A., Polinski, N. K., Paumier, K. L., Lipton, J. W., Steece-Collier, K., Collier, T. J., Buhlinger, D. J., & Sortwell, C. E. (2017). Subthalamic Nucleus Deep Brain Stimulation Employs trkB Signaling for Neuroprotection and Functional Restoration. *The Journal of Neuroscience: The Official Journal of the Society for Neuroscience*, *37*(28), 6786–6796.

Fischer, D. L., Manfredsson, F. P., Kemp, C. J., Cole-Strauss, A., Lipton, J. W., Duffy, M. F., Polinski, N. K., Steece-Collier, K., Collier, T. J., Gombash, S. E., Buhlinger, D. J., & Sortwell, C. E. (2017). Subthalamic Nucleus Deep Brain Stimulation Does Not Modify the Functional Deficits or Axonopathy Induced by Nigrostriatal α-Synuclein Overexpression. *Scientific Reports*, *7*(1), 16356.

Fleischer, M., Endres, H., Sendtner, M., & Volkmann, J. (2020). Development of a Fully Implantable Stimulator for Deep Brain Stimulation in Mice. *Frontiers in Neuroscience*, *14*, 726.

Fomenko, A., Lee, D. J., McKinnon, C., Lee, E. J., de Snoo, M. L., Gondard, E., Neudorfer, C., Hamani, C., Lozano, A. M., Kalia, L. V., & Kalia, S. K. (2020). Deep Brain Stimulation of the Medial Septal Nucleus Induces Expression of a Virally Delivered Reporter Gene in Dentate Gyrus. *Frontiers in Neuroscience*, *14*, 463.

Friedman, A., Lax, E., Abraham, L., Tischler, H., & Yadid, G. (2012). Abnormality of VTA local field potential in an animal model of depression was restored by patterned DBS treatment. *European Neuropsychopharmacology: The Journal of the European College of Neuropsychopharmacology*, *22*(1), 64–71.

Friedman, A., Lax, E., Dikshtein, Y., Abraham, L., Flaumenhaft, Y., Sudai, E., Ben-Tzion, M., Ami-Ad, L., Yaka, R., & Yadid, G. (2010). Electrical stimulation of the lateral habenula produces enduring inhibitory effect on cocaine seeking behavior. *Neuropharmacology*, *59*(6), 452–459.

Friedman, A., Lax, E., Dikshtein, Y., Abraham, L., Flaumenhaft, Y., Sudai, E., Ben-Tzion, M., & Yadid, G.

(2011). Electrical stimulation of the lateral habenula produces an inhibitory effect on sucrose self-administration. *Neuropharmacology*, *60*(2-3), 381–387.

Furlanetti, L. L., Coenen, V. A., Aranda, I. A., & Döbrössy, M. D. (2015). Chronic deep brain stimulation of the medial forebrain bundle reverses depressive-like behavior in a hemiparkinsonian rodent model. *Experimental Brain Research. Experimentelle Hirnforschung. Experimentation Cerebrale*, *233*(11), 3073–3085.

Furlanetti, L. L., Coenen, V. A., & Döbrössy, M. D. (2016). Ventral tegmental area dopaminergic lesion-induced depressive phenotype in the rat is reversed by deep brain stimulation of the medial forebrain bundle. *Behavioural Brain Research*, *299*, 132–140.

Furlanetti, L. L., Döbrössy, M. D., Aranda, I. A., & Coenen, V. A. (2015). Feasibility and safety of continuous and chronic bilateral deep brain stimulation of the medial forebrain bundle in the naïve Sprague-Dawley rat. *Behavioural Neurology*, *2015*, 256196.

Gallino, D., Devenyi, G. A., Germann, J., Guma, E., Anastassiadis, C., & Chakravarty, M. M. (2019). Longitudinal assessment of the neuroanatomical consequences of deep brain stimulation: Application of fornical DBS in an Alzheimer's mouse model. *Brain Research*, *1715*, 213–223.

Gao, F., Guo, Y., Zhang, H., Wang, S., Wang, J., Wu, J.-M., Chen, Z., & Ding, M.-P. (2009). Anterior thalamic nucleus stimulation modulates regional cerebral metabolism: an FDG-MicroPET study in rats. *Neurobiology of Disease*, *34*(3), 477–483.

Gardner, W., Fuchs, F., Durieux, L., Bourgin, P., Coenen, V. A., Döbrössy, M., & Lecourtier, L. (2022). Slow Wave Sleep Deficits in the Flinders Sensitive Line Rodent Model of Depression: Effects of Medial Forebrain Bundle Deep-Brain Stimulation. *Neuroscience*, *498*, 31–49.

Gazit, T., Friedman, A., Lax, E., Samuel, M., Zahut, R., Katz, M., Abraham, L., Tischler, H., Teicher, M., & Yadid, G. (2015). Programmed deep brain stimulation synchronizes VTA gamma band field potential and alleviates depressive-like behavior in rats. *Neuropharmacology*, *91*, 135–141.

Gee, L. E., Walling, I., Ramirez-Zamora, A., Shin, D. S., & Pilitsis, J. G. (2016). Subthalamic deep brain stimulation alters neuronal firing in canonical pain nuclei in a 6-hydroxydopamine lesioned rat model of Parkinson's disease. *Experimental Neurology*, *283*(Pt A), 298–307.

Gidyk, D. C., Diwan, M., Gouveia, F. V., Giacobbe, P., Lipsman, N., & Hamani, C. (2021). Investigating the role of CB1 endocannabinoid transmission in the anti-fear and anxiolytic-like effects of ventromedial prefrontal cortex deep brain stimulation. *Journal of Psychiatric Research*, *135*, 264–269.

Gimenes, C., Malheiros, J. M., Battapady, H., Tannus, A., Hamani, C., & Covolan, L. (2019). The neural response to deep brain stimulation of the anterior nucleus of the thalamus: A MEMRI and c-Fos study. *Brain Research Bulletin*, *147*, 133–139.

Gimenes, C., Motta Pollo, M. L., Diaz, E., Hargreaves, E. L., Boison, D., & Covolan, L. (2022). Deep brain stimulation of the anterior thalamus attenuates PTZ kindling with concomitant reduction of adenosine kinase expression in rats. *Brain Stimulation*, *15*(4), 892–901.

Gondard, E., Chau, H. N., Mann, A., Tierney, T. S., Hamani, C., Kalia, S. K., & Lozano, A. M. (2015). Rapid Modulation of Protein Expression in the Rat Hippocampus Following Deep Brain Stimulation of the Fornix. *Brain Stimulation*, *8*(6), 1058–1064.

Gondard, E., Teves, L., Wang, L., McKinnon, C., Hamani, C., Kalia, S. K., Carlen, P. L., Tymianski, M., & Lozano, A. M. (2019). Deep Brain Stimulation Rescues Memory and Synaptic Activity in a Rat Model of Global Ischemia. *The Journal of Neuroscience: The Official Journal of the Society for Neuroscience*, *39*(13), 2430–2440.

Grembecka, B., Glac, W., Listowska, M., Jerzemowska, G., Plucińska, K., Majkutewicz, I., Badtke, P., & Wrona, D. (2021). Subthalamic Deep Brain Stimulation Affects Plasma Corticosterone Concentration and Peripheral Immunity Changes in Rat Model of Parkinson's Disease. *Journal of Neuroimmune Pharmacology: The Official Journal of the Society on NeuroImmune Pharmacology*, *16*(2), 454–469.

Guercio, L. A., Schmidt, H. D., & Pierce, R. C. (2015). Deep brain stimulation of the nucleus accumbens shell attenuates cue-induced reinstatement of both cocaine and sucrose seeking in rats. *Behavioural Brain Research*, *281*, 125–130.

Guercio, L. A., Wimmer, M. E., Schmidt, H. D., Swinford-Jackson, S. E., Pierce, R. C., & Vassoler, F. M. (2020). Deep brain stimulation of the infralimbic cortex attenuates cocaine priming-induced reinstatement of drug seeking. *Brain Research*, *1746*, 147011.

Guimarães, J., Moura, E., Silva, E., Aguiar, P., Garrett, C., & Vieira-Coelho, M. A. (2013). Locus coeruleus is involved in weight loss in a rat model of Parkinson's disease: an effect reversed by deep brain stimulation. *Brain Stimulation*, *6*(6), 845–855.

Guo, H., Zhang, H., Kuang, Y., Wang, C., Jing, X., Gu, J., & Gao, G. (2014). Electrical stimulation of the substantia nigra pars reticulata (SNr) suppresses chemically induced neocortical seizures in rats. *Journal of Molecular Neuroscience: MN*, *53*(4), 546–552.

Guo, L., Zhou, H., Wang, R., Xu, J., Zhou, W., Zhang, F., Tang, S., Liu, H., & Jiang, J. (2013). DBS of nucleus accumbens on heroin seeking behaviors in self-administering rats. *Drug and Alcohol Dependence*, *129*(1-2), 70–81.

Gut, N. K., & Winn, P. (2015). Deep brain stimulation of different pedunculopontine targets in a novel rodent model of parkinsonism. *The Journal of Neuroscience: The Official Journal of the Society for Neuroscience*, *35*(12), 4792–4803.

Hadar, R., Bikovski, L., Soto-Montenegro, M. L., Schimke, J., Maier, P., Ewing, S., Voget, M., Wieske, F., Götz, T., Desco, M., Hamani, C., Pascau, J., Weiner, I., & Winter, C. (2018). Early neuromodulation prevents the development of brain and behavioral abnormalities in a rodent model of schizophrenia. *Molecular Psychiatry*, *23*(4), 943–951.

Hagains, C. E., He, J.-W., Chiao, J.-C., & Peng, Y. B. (2011). Septal stimulation inhibits spinal cord dorsal horn neuronal activity. *Brain Research*, *1382*, 189–197.

Halpern, C. H., Tekriwal, A., Santollo, J., Keating, J. G., Wolf, J. A., Daniels, D., & Bale, T. L. (2013). Amelioration of binge eating by nucleus accumbens shell deep brain stimulation in mice involves D2 receptor modulation. *The Journal of Neuroscience: The Official Journal of the Society for Neuroscience*, *33*(17), 7122–7129.

Hamani, C., Amorim, B. O., Wheeler, A. L., Diwan, M., Driesslein, K., Covolan, L., Butson, C. R., & Nobrega, J. N. (2014). Deep brain stimulation in rats: different targets induce similar antidepressant-like effects but influence different circuits. *Neurobiology of Disease*, *71*, 205–214.

Hamani, C., Diwan, M., Isabella, S., Lozano, A. M., & Nobrega, J. N. (2010). Effects of different stimulation parameters on the antidepressant-like response of medial prefrontal cortex deep brain stimulation in rats. *Journal of Psychiatric Research*, *44*(11), 683–687.

Hamani, C., Diwan, M., Macedo, C. E., Brandão, M. L., Shumake, J., Gonzalez-Lima, F., Raymond, R., Lozano, A. M., Fletcher, P. J., & Nobrega, J. N. (2010). Antidepressant-like effects of medial prefrontal cortex deep brain stimulation in rats. *Biological Psychiatry*, *67*(2), 117–124.

Hamani, C., Dubiela, F. P., Soares, J. C. K., Shin, D., Bittencourt, S., Covolan, L., Carlen, P. L., Laxton, A. W., Hodaie, M., Stone, S. S. D., Ha, Y., Hutchison, W. D., Lozano, A. M., Mello, L. E., & Oliveira, M. G. M. (2010). Anterior thalamus deep brain stimulation at high current impairs memory in rats. *Experimental Neurology*, *225*(1), 154–162.

Hamani, C., Machado, D. C., Hipólide, D. C., Dubiela, F. P., Suchecki, D., Macedo, C. E., Tescarollo, F., Martins, U., Covolan, L., & Nobrega, J. N. (2012). Deep brain stimulation reverses anhedonic-like behavior in a chronic model of depression: role of serotonin and brain derived neurotrophic factor. *Biological Psychiatry*, *71*(1), 30–35.

Hameleers, R., Blokland, A., Steinbusch, H. W. M., Visser-Vandewalle, V., & Temel, Y. (2007). Hypomobility after DOI administration can be reversed by subthalamic nucleus deep brain stimulation. *Behavioural Brain Research*, *185*(1), 65–67.

Hamilton, J. I., Pahwa, S., Adedigba, J., Frankel, S., O'Connor, G., Thomas, R., Walker, J. R., Killinc, O., Lo, W.-C., Batesole, J., Margevicius, S., Griswold, M., Rajagopalan, S., Gulani, V., & Seiberlich, N. (2020). Simultaneous Mapping of T and T Using Cardiac Magnetic Resonance Fingerprinting in a Cohort of Healthy Subjects at 1.5T. *Journal of Magnetic Resonance Imaging: JMRI*, *52*(4), 1044–1052.

Hamilton, J., Lee, J., & Canales, J. J. (2015). Chronic unilateral stimulation of the nucleus accumbens at high

or low frequencies attenuates relapse to cocaine seeking in an animal model. *Brain Stimulation*, *8*(1), 57–63.

Hao, S., Tang, B., Wu, Z., Ure, K., Sun, Y., Tao, H., Gao, Y., Patel, A. J., Curry, D. J., Samaco, R. C., Zoghbi, H. Y., & Tang, J. (2015). Forniceal deep brain stimulation rescues hippocampal memory in Rett syndrome mice. *Nature*, *526*(7573), 430–434.

Hao, S., Wang, Q., Tang, B., Wu, Z., Yang, T., & Tang, J. (2021). CDKL5 Deficiency Augments Inhibitory Input into the Dentate Gyrus That Can Be Reversed by Deep Brain Stimulation. *The Journal of Neuroscience: The Official Journal of the Society for Neuroscience*, *41*(43), 9031–9046.

Hashtjini, M. M., Jahromi, G. P., Sadr, S. S., Meftahi, G. H., Hatef, B., & Javidnazar, D. (2018). Deep brain stimulation in a rat model of post-traumatic stress disorder modifies forebrain neuronal activity and serum corticosterone. *Iranian Journal of Basic Medical Sciences*, *21*(4), 370–375.

Henderson, M. B., Green, A. I., Bradford, P. S., Chau, D. T., Roberts, D. W., & Leiter, J. C. (2010). Deep brain stimulation of the nucleus accumbens reduces alcohol intake in alcohol-preferring rats. *Neurosurgical Focus*, *29*(2), E12.

Hermann, J. K., Borseth, A., Pucci, F. G., Toth, C., Hogue, O., Chan, H. H., Machado, A. G., & Baker, K. B. (2022). Changes in somatosensory evoked potentials elicited by lateral cerebellar nucleus deep brain stimulation in the naïve rodent. *Neuroscience Letters*, *786*, 136800.

Hescham, S., Jahanshahi, A., Meriaux, C., Lim, L. W., Blokland, A., & Temel, Y. (2015). Behavioral effects of deep brain stimulation of different areas of the Papez circuit on memory- and anxiety-related functions. *Behavioural Brain Research*, *292*, 353–360.

Hescham, S., Jahanshahi, A., Schweimer, J. V., Mitchell, S. N., Carter, G., Blokland, A., Sharp, T., & Temel, Y. (2016). Fornix deep brain stimulation enhances acetylcholine levels in the hippocampus. *Brain Structure & Function*, *221*(8), 4281–4286.

Hescham, S., Lim, L. W., Jahanshahi, A., Steinbusch, H. W. M., Prickaerts, J., Blokland, A., & Temel, Y. (2013). Deep brain stimulation of the forniceal area enhances memory functions in experimental dementia: the role of stimulation parameters. *Brain Stimulation*, *6*(1), 72–77.

Hescham, S., Temel, Y., Schipper, S., Lagiere, M., Schönfeld, L.-M., Blokland, A., & Jahanshahi, A. (2017). Fornix deep brain stimulation induced long-term spatial memory independent of hippocampal neurogenesis. *Brain Structure & Function*, *222*(2), 1069–1075.

He, Z., Jiang, Y., Xu, H., Jiang, H., Jia, W., Sun, P., & Xie, J. (2014). High frequency stimulation of subthalamic nucleus results in behavioral recovery by increasing striatal dopamine release in 6-hydroxydopamine lesioned rat. *Behavioural Brain Research*, *263*, 108–114.

Ho, A. L., Feng, A. Y., Barbosa, D. A. N., Wu, H., Smith, M. L., Malenka, R. C., Tass, P. A., & Halpern, C. H. (2021). Accumbens coordinated reset stimulation in mice exhibits ameliorating aftereffects on binge alcohol drinking. *Brain Stimulation*, *14*(2), 330–334.

Ho, D. X. K., Tan, Y. C., Tan, J., Too, H. P., & Ng, W. H. (2014). High-frequency stimulation of the globus pallidus interna nucleus modulates GFRα1 gene expression in the basal ganglia. *Journal of Clinical Neuroscience: Official Journal of the Neurosurgical Society of Australasia*, *21*(4), 657–660.

Hofer, A.-S., Scheuber, M. I., Sartori, A. M., Good, N., Stalder, S. A., Hammer, N., Fricke, K., Schalbetter, S. M., Engmann, A. K., Weber, R. Z., Rust, R., Schneider, M. P., Russi, N., Favre, G., & Schwab, M. E. (2022). Stimulation of the cuneiform nucleus enables training and boosts recovery after spinal cord injury. *Brain: A Journal of Neurology*, *145*(10), 3681–3697.

Huang, C., Chu, H., Ma, Y., Zhou, Z., Dai, C., Huang, X., Fang, L., Ao, Q., & Huang, D. (2019). The neuroprotective effect of deep brain stimulation at nucleus basalis of Meynert in transgenic mice with Alzheimer's disease. *Brain Stimulation*, *12*(1), 161–174.

Huotarinen, A., Leino, S., Tuominen, R. K., & Laakso, A. (2019). Rat subthalamic stimulation: Evaluating stimulation-induced dyskinesias, choosing stimulation currents and evaluating the anti-akinetic effect in the cylinder test. *MethodsX*, *6*, 2384–2395.

Huotarinen, A., Penttinen, A.-M., Bäck, S., Voutilainen, M. H., Julku, U., Piepponen, T. P., Männistö, P. T., Saarma, M., Tuominen, R., Laakso, A., & Airavaara, M. (2018). Combination of CDNF and Deep

Brain Stimulation Decreases Neurological Deficits in Late-stage Model Parkinson's Disease. *Neuroscience*, *374*, 250–263.

Ihme, H., Schwarting, R. K. W., & Melo-Thomas, L. (2020). Low frequency deep brain stimulation in the inferior colliculus ameliorates haloperidol-induced catalepsy and reduces anxiety in rats. *PloS One*, *15*(12), e0243438.

Insel, N., Pilkiw, M., Nobrega, J. N., Hutchison, W. D., Takehara-Nishiuchi, K., & Hamani, C. (2015). Chronic deep brain stimulation of the rat ventral medial prefrontal cortex disrupts hippocampal-prefrontal coherence. *Experimental Neurology*, *269*, 1–7.

Jackson, J., Young, C. K., Hu, B., & Bland, B. H. (2008). High frequency stimulation of the posterior hypothalamic nucleus restores movement and reinstates hippocampal-striatal theta coherence following haloperidol-induced catalepsy. *Experimental Neurology*, *213*(1), 210–219.

Jakobs, M., Pitzer, C., Sartorius, A., Unterberg, A., & Kiening, K. (2019). Acute 5 Hz deep brain stimulation of the lateral habenula is associated with depressive-like behavior in male wild-type Wistar rats. *Brain Research*, *1721*, 146283.

Jeong, D. U., Lee, J., Chang, W. S., & Chang, J. W. (2017). Identifying the appropriate time for deep brain stimulation to achieve spatial memory improvement on the Morris water maze. *BMC Neuroscience*, *18*(1), 29.

Jeong, D. U., Lee, J. E., Lee, S. E., Chang, W. S., Kim, S. J., & Chang, J. W. (2014). Improvements in memory after medial septum stimulation are associated with changes in hippocampal cholinergic activity and neurogenesis. *BioMed Research International*, *2014*, 568587.

Jia, L., Sun, Z., Shi, D., Wang, M., Jia, J., He, Y., Xue, F., Ren, Y., Yang, J., & Ma, X. (2019). Effects of different patterns of electric stimulation of the ventromedial prefrontal cortex on hippocampal-prefrontal coherence in a rat model of depression. *Behavioural Brain Research*, *356*, 179–188.

Jiang, Y., Liu, D.-F., Zhang, X., Liu, H.-G., Zhang, C., & Zhang, J.-G. (2022). Modulation of the rat hippocampal-cortex network and episodic-like memory performance following entorhinal cortex stimulation. *CNS Neuroscience & Therapeutics*, *28*(3), 448–457.

Jiménez-Sánchez, L., Castañé, A., Pérez-Caballero, L., Grifoll-Escoda, M., López-Gil, X., Campa, L., Galofré, M., Berrocoso, E., & Adell, A. (2016). Activation of AMPA Receptors Mediates the Antidepressant Action of Deep Brain Stimulation of the Infralimbic Prefrontal Cortex. *Cerebral Cortex* , *26*(6), 2778–2789.

Jiménez-Sánchez, L., Linge, R., Campa, L., Valdizán, E. M., Pazos, Á., Díaz, Á., & Adell, A. (2016). Behavioral, neurochemical and molecular changes after acute deep brain stimulation of the infralimbic prefrontal cortex. *Neuropharmacology*, *108*, 91–102.

Jokara, Z., Khatamsaz, S., Alaei, H., & Shariati, M. (2022). Effect of electrical stimulation of central nucleus of the amygdala on morphine conditioned place preference in male rats. *Iranian Journal of Basic Medical Sciences*, *25*(5), 604–610.

Jouve, L., Salin, P., Melon, C., & Kerkerian-Le Goff, L. (2010). Deep brain stimulation of the center median-parafascicular complex of the thalamus has efficient anti-parkinsonian action associated with widespread cellular responses in the basal ganglia network in a rat model of Parkinson's disease. *The Journal of Neuroscience: The Official Journal of the Society for Neuroscience*, *30*(29), 9919–9928.

Kale, R. P., Nguyen, T. T. L., Price, J. B., Yates, N. J., Walder, K., Berk, M., Sillitoe, R. V., Kouzani, A. Z., & Tye, S. J. (2021). Mood Regulatory Actions of Active and Sham Nucleus Accumbens Deep Brain Stimulation in Antidepressant Resistant Rats. *Frontiers in Human Neuroscience*, *15*, 644921.

Kallupi, M., Kononoff, J., Melas, P. A., Qvist, J. S., de Guglielmo, G., Kandel, E. R., & George, O. (2022). Deep brain stimulation of the nucleus accumbens shell attenuates cocaine withdrawal but increases cocaine self-administration, cocaine-induced locomotor activity, and GluR1/GluA1 in the central nucleus of the amygdala in male cocaine-dependent rats. *Brain Stimulation*, *15*(1), 13–22.

Kaminer, J., Thakur, P., & Evinger, C. (2015). Effects of subthalamic deep brain stimulation on blink abnormalities of 6-OHDA lesioned rats. *Journal of Neurophysiology*, *113*(9), 3038–3046.

Kaszuba, B. C., Maietta, T., Walling, I., Feustel, P., Stapleton, A., Shin, D. S., Slyer, J., & Pilitsis, J. G. (2019). Effects of subthalamic deep brain stimulation with gabapentin and morphine on mechanical and thermal thresholds in 6-hydroxydopamine lesioned rats. *Brain Research*, *1715*, 66–72.

Kaszuba, B. C., Walling, I., Gee, L. E., Shin, D. S., & Pilitsis, J. G. (2017). Effects of subthalamic deep brain stimulation with duloxetine on mechanical and thermal thresholds in 6OHDA lesioned rats. *Brain Research*, *1655*, 233–241.

Keenan, D. M., Quinkert, A. W., & Pfaff, D. W. (2015). Stochastic modeling of mouse motor activity under deep brain stimulation: the extraction of arousal information. *PLoS Computational Biology*, *11*(2), e1003883.

Kerkerian-Le Goff, L., Jouve, L., Melon, C., & Salin, P. (2009). Rationale for targeting the thalamic centre-median parafascicular complex in the surgical treatment of Parkinson's disease. *Parkinsonism & Related Disorders*, *15 Suppl 3*, S167–S170.

Khodadadi, M., Zare, M., Rezaei, M., Bakhtiarzadeh, F., Barkley, V., Shojaei, A., Raoufy, M. R., & Mirnajafi-Zadeh, J. (2022). Effect of low frequency stimulation of olfactory bulb on seizure severity, learning, and memory in kindled rats. *Epilepsy Research*, *188*, 107055.

Kile, K. B., Tian, N., & Durand, D. M. (2010). Low frequency stimulation decreases seizure activity in a mutation model of epilepsy. *Epilepsia*, *51*(9), 1745–1753.

Kim, J., Eun Lee, S., Sik Min, K., Jung, H. H., Lee, J. E., Kim, S. J., & Chang, J. W. (2013). Ventral posterolateral deep brain stimulation treatment for neuropathic pain shortens pain response after cold stimuli. *Journal of Neuroscience Research*, *91*(7), 997–1004.

Kim, J., Kim, J., Min, K. S., Lee, S. E., Kim, S. J., & Chang, J. W. (2012). VPL-DBS on neuropathic pain rat model is effective in mechanical allodynia than cold allodynia. *Neurological Sciences: Official Journal of the Italian Neurological Society and of the Italian Society of Clinical Neurophysiology*, *33*(6), 1265–1270.

Kim, J., Lee, S. E., Shin, J., Jung, H. H., Kim, S. J., & Chang, J. W. (2015). The neuromodulation of neuropathic pain by measuring pain response rate and pain response duration in animal. *Journal of Korean Neurosurgical Society*, *57*(1), 6–11.

Kim, Y., McGee, S., Czeczor, J. K., Walker, A. J., Kale, R. P., Kouzani, A. Z., Walder, K., Berk, M., & Tye, S. J. (2016). Nucleus accumbens deep-brain stimulation efficacy in ACTH-pretreated rats: alterations in mitochondrial function relate to antidepressant-like effects. *Translational Psychiatry*, *6*(6), e842.

Kim, Y., Morath, B., Hu, C., Byrne, L. K., Sutor, S. L., Frye, M. A., & Tye, S. J. (2016). Antidepressant actions of lateral habenula deep brain stimulation differentially correlate with CaMKII/GSK3/AMPK signaling locally and in the infralimbic cortex. *Behavioural Brain Research*, *306*, 170–177.

King, N. O., Anderson, C. J., & Dorval, A. D. (2016). Deep brain stimulation exacerbates hypokinetic dysarthria in a rat model of Parkinson's disease. *Journal of Neuroscience Research*, *94*(2), 128–138.

Klanker, M., Feenstra, M., Willuhn, I., & Denys, D. (2017). Deep brain stimulation of the medial forebrain bundle elevates striatal dopamine concentration without affecting spontaneous or reward-induced phasic release. *Neuroscience*, *364*, 82–92.

Klanker, M., Post, G., Joosten, R., Feenstra, M., & Denys, D. (2013). Deep brain stimulation in the lateral orbitofrontal cortex impairs spatial reversal learning. *Behavioural Brain Research*, *245*, 7–12.

Klein, J., Hadar, R., Götz, T., Männer, A., Eberhardt, C., Baldassarri, J., Schmidt, T. T., Kupsch, A., Heinz, A., Morgenstern, R., Schneider, M., Weiner, I., & Winter, C. (2013). Mapping brain regions in which deep brain stimulation affects schizophrenia-like behavior in two rat models of schizophrenia. *Brain Stimulation*, *6*(4), 490–499.

Knapp, C. M., Tozier, L., Pak, A., Ciraulo, D. A., & Kornetsky, C. (2009). Deep brain stimulation of the nucleus accumbens reduces ethanol consumption in rats. *Pharmacology, Biochemistry, and Behavior*, *92*(3), 474–479.

Kocabicak, E., Jahanshahi, A., Schonfeld, L., Hescham, S.-A., Temel, Y., & Tan, S. (2015). Deep Brain Stimulation of the Rat Subthalamic Nucleus Induced Inhibition of Median Raphe Serotonergic and Dopaminergic Neurotransmission. *Turkish Neurosurgery*, *25*(5), 721–727.

Koulousakis, P., van den Hove, D., Visser-Vandewalle, V., & Sesia, T. (2020). Cognitive Improvements After Intermittent Deep Brain Stimulation of the Nucleus Basalis of Meynert in a Transgenic Rat Model for Alzheimer's Disease: A Preliminary Approach. *Journal of Alzheimer's Disease: JAD*, *73*(2), 461–466.

Krämer, S. D., Schuhmann, M. K., Schadt, F., Israel, I., Samnick, S., Volkmann, J., & Fluri, F. (2022). Changes of cerebral network activity after invasive stimulation of the mesencephalic locomotor region in a rat stroke model. *Experimental Neurology*, *347*, 113884.

Kumar, G., Asthana, P., Yung, W. H., Kwan, K. M., Tin, C., & Ma, C. H. E. (2022). Deep Brain Stimulation of the Interposed Nucleus Reverses Motor Deficits and Stimulates Production of Anti-inflammatory Cytokines in Ataxia Mice. *Molecular Neurobiology*, *59*(7), 4578–4592.

Lai, H.-Y., Younce, J. R., Albaugh, D. L., Kao, Y.-C. J., & Shih, Y.-Y. I. (2014). Functional MRI reveals frequency-dependent responses during deep brain stimulation at the subthalamic nucleus or internal globus pallidus. *NeuroImage*, *84*, 11–18.

Langevin, J.-P., De Salles, A. A. F., Kosoyan, H. P., & Krahl, S. E. (2010). Deep brain stimulation of the amygdala alleviates post-traumatic stress disorder symptoms in a rat model. *Journal of Psychiatric Research*, *44*(16), 1241–1245.

Laver, B., Diwan, M., Nobrega, J. N., & Hamani, C. (2014). Augmentative therapies do not potentiate the antidepressant-like effects of deep brain stimulation in rats. *Journal of Affective Disorders*, *161*, 87–90.

Lee, D. J., Gurkoff, G. G., Izadi, A., Seidl, S. E., Echeverri, A., Melnik, M., Berman, R. F., Ekstrom, A. D., Muizelaar, J. P., Lyeth, B. G., & Shahlaie, K. (2015). Septohippocampal Neuromodulation Improves Cognition after Traumatic Brain Injury. *Journal of Neurotrauma*, *32*(22), 1822–1832.

Lee, H.-J., Sung, J. H., Hong, J. T., Kim, I. S., Yang, S. H., & Cho, C. B. (2019). Change of Extracellular Glutamate Level in Striatum during Deep Brain Stimulation of the Entopeduncular Nucleus in Rats. *Journal of Korean Neurosurgical Society*, *62*(2), 166–174.

Lee, J., & Chang, S.-Y. (2019). Altered Primary Motor Cortex Neuronal Activity in a Rat Model of Harmaline-Induced Tremor During Thalamic Deep Brain Stimulation. *Frontiers in Cellular Neuroscience*, *13*, 448.

Lee, J., Kim, J., Cortez, J., & Chang, S.-Y. (2022). Thalamo-cortical network is associated with harmaline-induced tremor in rodent model. *Experimental Neurology*, *358*, 114210.

Lee, K. H., Kristic, K., van Hoff, R., Hitti, F. L., Blaha, C., Harris, B., Roberts, D. W., & Leiter, J. C. (2007). High-frequency stimulation of the subthalamic nucleus increases glutamate in the subthalamic nucleus of rats as demonstrated by in vivo enzyme-linked glutamate sensor. *Brain Research*, *1162*, 121–129.

Lee, L.-H. N., Huang, C.-S., Wang, R.-W., Lai, H.-J., Chung, C.-C., Yang, Y.-C., & Kuo, C.-C. (2022). Deep brain stimulation rectifies the noisy cortex and irresponsive subthalamus to improve parkinsonian locomotor activities. *NPJ Parkinson's Disease*, *8*(1), 77.

Lehto, L. J., Canna, A., Wu, L., Sierra, A., Zhurakovskaya, E., Ma, J., Pearce, C., Shaio, M., Filip, P., Johnson, M. D., Low, W. C., Gröhn, O., Tanila, H., Mangia, S., & Michaeli, S. (2020). Orientation selective deep brain stimulation of the subthalamic nucleus in rats. *NeuroImage*, *213*, 116750.

Lehto, L. J., Filip, P., Laakso, H., Sierra, A., Slopsema, J. P., Johnson, M. D., Eberly, L. E., Low, W. C., Gröhn, O., Tanila, H., Mangia, S., & Michaeli, S. (2018). Tuning Neuromodulation Effects by Orientation Selective Deep Brain Stimulation in the Rat Medial Frontal Cortex. *Frontiers in Neuroscience*, *12*, 899.

Leplus, A., Lauritzen, I., Melon, C., Kerkerian-Le Goff, L., Fontaine, D., & Checler, F. (2019). Chronic fornix deep brain stimulation in a transgenic Alzheimer's rat model reduces amyloid burden, inflammation, and neuronal loss. *Brain Structure & Function*, *224*(1), 363–372.

Li, H.-T., Donegan, D. C., Peleg-Raibstein, D., & Burdakov, D. (2022). Hypothalamic deep brain stimulation as a strategy to manage anxiety disorders. *Proceedings of the National Academy of Sciences of the United States of America*, *119*(16), e2113518119.

Lim, L. W., Blokland, A., Visser-Vandewalle, V., Vlamings, R., Sesia, T., Steinbusch, H., Schruers, K., Griez, E., & Temel, Y. (2008). High-frequency stimulation of the dorsolateral periaqueductal gray and ventromedial hypothalamus fails to inhibit panic-like behaviour. *Behavioural Brain Research*, *193*(2),

197–203.
Lim, L. W., Prickaerts, J., Huguet, G., Kadar, E., Hartung, H., Sharp, T., & Temel, Y. (2015). Electrical stimulation alleviates depressive-like behaviors of rats: investigation of brain targets and potential mechanisms. *Translational Psychiatry*, *5*(3), e535.
Lindemann, C., Krauss, J. K., & Schwabe, K. (2012). Deep brain stimulation of the subthalamic nucleus in the 6-hydroxydopamine rat model of Parkinson's disease: effects on sensorimotor gating. *Behavioural Brain Research*, *230*(1), 243–250.
Lin, H.-C., Pan, H.-C., Lin, S.-H., Lo, Y.-C., Shen, E. T.-H., Liao, L.-D., Liao, P.-H., Chien, Y.-W., Liao, K.-D., Jaw, F.-S., Chu, K.-W., Lai, H.-Y., & Chen, Y.-Y. (2015). Central Thalamic Deep-Brain Stimulation Alters Striatal-Thalamic Connectivity in Cognitive Neural Behavior. *Frontiers in Neural Circuits*, *9*, 87.
Lin, T.-C., Lo, Y.-C., Lin, H.-C., Li, S.-J., Lin, S.-H., Wu, H.-F., Chu, M.-C., Lee, C.-W., Lin, I.-C., Chang, C.-W., Liu, Y.-C., Chen, T.-C., Lin, Y.-J., Ian Shih, Y.-Y., & Chen, Y.-Y. (2019). MR imaging central thalamic deep brain stimulation restored autistic-like social deficits in the rat. *Brain Stimulation*, *12*(6), 1410–1420.
Li, S., Arbuthnott, G. W., Jutras, M. J., Goldberg, J. A., & Jaeger, D. (2007). Resonant antidromic cortical circuit activation as a consequence of high-frequency subthalamic deep-brain stimulation. *Journal of Neurophysiology*, *98*(6), 3525–3537.
Li, S.-J., Lo, Y.-C., Lai, H.-Y., Lin, S.-H., Lin, H.-C., Lin, T.-C., Chang, C.-W., Chen, T.-C., Chin-Jung Hsieh, C., Yang, S.-H., Chiu, F.-M., Kuo, C.-H., & Chen, Y.-Y. (2020). Uncovering the Modulatory Interactions of Brain Networks in Cognition with Central Thalamic Deep Brain Stimulation Using Functional Magnetic Resonance Imaging. *Neuroscience*, *440*, 65–84.
Liu, D.-F., Chen, Y.-C., Zhu, G.-Y., Wang, X., Jiang, Y., Liu, H.-G., & Zhang, J.-G. (2020). Effects of anterior thalamic nuclei stimulation on gene expression in a rat model of temporal lobe epilepsy. *Acta Neurologica Belgica*, *120*(6), 1361–1370.
Liu, H., Wolters, A., Temel, Y., Alosaimi, F., Jahanshahi, A., & Hescham, S. (2022). Deep brain stimulation of the nucleus basalis of Meynert in an experimental rat model of dementia: Stimulation parameters and mechanisms. *Neurobiology of Disease*, *171*, 105797.
Liu, H.-Y., Jin, J., Tang, J.-S., Sun, W.-X., Jia, H., Yang, X.-P., Cui, J.-M., & Wang, C.-G. (2008). Chronic deep brain stimulation in the rat nucleus accumbens and its effect on morphine reinforcement. *Addiction Biology*, *13*(1), 40–46.
Li, X.-H., Wang, J.-Y., Gao, G., Chang, J.-Y., Woodward, D. J., & Luo, F. (2010). High-frequency stimulation of the subthalamic nucleus restores neural and behavioral functions during reaction time task in a rat model of Parkinson's disease. *Journal of Neuroscience Research*, *88*(7), 1510–1521.
Li, Z., Guo, Y., Bao, X., Lei, J., Shen, Z., Wang, X., Li, L., Li, Y., & Wang, R. (2021). Effects of Subthalamic Deep Brain Stimulation With Different Frequencies in a Parkinsonian Rat Model. *Neuromodulation: Journal of the International Neuromodulation Society*, *24*(2), 220–228.
Lu, C., Feng, Y., Li, H., Gao, Z., Zhu, X., & Hu, J. (2022). A preclinical study of deep brain stimulation in the ventral tegmental area for alleviating positive psychotic-like behaviors in mice. *Frontiers in Human Neuroscience*, *16*, 945912.
Lu, H., Ash, R. T., He, L., Kee, S. E., Wang, W., Yu, D., Hao, S., Meng, X., Ure, K., Ito-Ishida, A., Tang, B., Sun, Y., Ji, D., Tang, J., Arenkiel, B. R., Smirnakis, S. M., & Zoghbi, H. Y. (2016). Loss and Gain of MeCP2 Cause Similar Hippocampal Circuit Dysfunction that Is Rescued by Deep Brain Stimulation in a Rett Syndrome Mouse Model. *Neuron*, *91*(4), 739–747.
Luyck, K., Tambuyzer, T., Deprez, M., Rangarajan, J., Nuttin, B., & Luyten, L. (2017). Electrical stimulation of the bed nucleus of the stria terminalis reduces anxiety in a rat model. *Translational Psychiatry*, *7*(2), e1033.
Magdaleno-Madrigal, V. M., Contreras-Murillo, G., Camacho-Abrego, I., Negrete-Díaz, J. V., Valdés-Cruz, A., Fernández-Mas, R., Almazán-Alvarado, S., & Flores, G. (2017). Short-term deep brain stimulation of the thalamic reticular nucleus modifies aberrant oscillatory activity in a neurodevelopment model of

schizophrenia. *Neuroscience*, *357*, 99–109.
Magdaleno-Madrigal, V. M., Pantoja-Jiménez, C. R., Bazaldúa, A., Fernández-Mas, R., Almazán-Alvarado, S., Bolaños-Alejos, F., Ortíz-López, L., & Ramírez-Rodriguez, G. B. (2016). Acute deep brain stimulation in the thalamic reticular nucleus protects against acute stress and modulates initial events of adult hippocampal neurogenesis. *Behavioural Brain Research*, *314*, 65–76.
Mahoney, E. C., Zeng, A., Yu, W., Rowe, M., Sahai, S., Feustel, P. J., Ramirez-Zamora, A., Pilitsis, J. G., & Shin, D. S. (2018). Ventral pallidum deep brain stimulation attenuates acute partial, generalized and tonic-clonic seizures in two rat models. *Epilepsy Research*, *142*, 36–44.
Ma, J., & Leung, L. S. (2014). Deep brain stimulation of the medial septum or nucleus accumbens alleviates psychosis-relevant behavior in ketamine-treated rats. *Behavioural Brain Research*, *266*, 174–182.
Mann, A., Gondard, E., Tampellini, D., Milsted, J. A. T., Marillac, D., Hamani, C., Kalia, S. K., & Lozano, A. M. (2018). Chronic deep brain stimulation in an Alzheimer's disease mouse model enhances memory and reduces pathological hallmarks. *Brain Stimulation*, *11*(2), 435–444.
Martínez-Rivera, F. J., Rodriguez-Romaguera, J., Lloret-Torres, M. E., Do Monte, F. H., Quirk, G. J., & Barreto-Estrada, J. L. (2016). Bidirectional Modulation of Extinction of Drug Seeking by Deep Brain Stimulation of the Ventral Striatum. *Biological Psychiatry*, *80*(9), 682–690.
Martins Pereira, R. C., Medeiros, P., Coimbra, N. C., Machado, H. R., & de Freitas, R. L. (2022). Cortical Neurostimulation and N-Methyl-D-Aspartate Glutamatergic Receptor Activation in the Dysgranular Layer of the Posterior Insular Cortex Modulate Chronic Neuropathic Pain. *Neuromodulation: Journal of the International Neuromodulation Society*. https://doi.org/10.1016/j.neurom.2022.05.009
Ma, W., Feng, Z., Hu, H., Wang, Z., & Zhou, W. (2018). Synchronous Responses of Population Neurons to the Changes of Inter-Pulse-Intervals during Stimulations of Afferent Fibers. *Conference Proceedings: ... Annual International Conference of the IEEE Engineering in Medicine and Biology Society. IEEE Engineering in Medicine and Biology Society. Conference*, *2018*, 2178–2181.
McConnell, G. C., So, R. Q., & Grill, W. M. (2016). Failure to suppress low-frequency neuronal oscillatory activity underlies the reduced effectiveness of random patterns of deep brain stimulation. *Journal of Neurophysiology*, *115*(6), 2791–2802.
McConnell, G. C., So, R. Q., Hilliard, J. D., Lopomo, P., & Grill, W. M. (2012). Effective deep brain stimulation suppresses low-frequency network oscillations in the basal ganglia by regularizing neural firing patterns. *The Journal of Neuroscience: The Official Journal of the Society for Neuroscience*, *32*(45), 15657–15668.
McCracken, C. B., & Grace, A. A. (2007). High-frequency deep brain stimulation of the nucleus accumbens region suppresses neuronal activity and selectively modulates afferent drive in rat orbitofrontal cortex in vivo. *The Journal of Neuroscience: The Official Journal of the Society for Neuroscience*, *27*(46), 12601–12610.
McCracken, C. B., & Grace, A. A. (2009). Nucleus accumbens deep brain stimulation produces region-specific alterations in local field potential oscillations and evoked responses in vivo. *The Journal of Neuroscience: The Official Journal of the Society for Neuroscience*, *29*(16), 5354–5363.
McCracken, C. B., & Kiss, Z. H. T. (2014). Time and frequency-dependent modulation of local field potential synchronization by deep brain stimulation. *PloS One*, *9*(7), e102576.
Meissner, W., Harnack, D., Paul, G., Reum, T., Sohr, R., Morgenstern, R., & Kupsch, A. (2002). Deep brain stimulation of subthalamic neurons increases striatal dopamine metabolism and induces contralateral circling in freely moving 6-hydroxydopamine-lesioned rats. *Neuroscience Letters*, *328*(2), 105–108.
Melon, C., Chassain, C., Bielicki, G., Renou, J.-P., Kerkerian-Le Goff, L., Salin, P., & Durif, F. (2015). Progressive brain metabolic changes under deep brain stimulation of subthalamic nucleus in parkinsonian rats. *Journal of Neurochemistry*, *132*(6), 703–712.
Melo-Thomas, L., Gil-Martínez, A. L., Cuenca, L., Estrada, C., Gonzalez-Cuello, A., Schwarting, R. K., & Herrero, M. T. (2018). Electrical stimulation or MK-801 in the inferior colliculus improve motor deficits in MPTP-treated mice. *Neurotoxicology*, *65*, 38–43.
Melo-Thomas, L., Tacken, L., Richter, N., Almeida, D., Rapôso, C., de Melo, S. R., Thomas, U., de Paiva, Y.

B., Medeiros, P., Coimbra, N. C., & Schwarting, R. (2022). Lateralization in hemi-parkinsonian rats is affected by deep brain stimulation or glutamatergic neurotransmission in the inferior colliculus. *eNeuro*, *9*(4). https://doi.org/10.1523/ENEURO.0076-22.2022

Melo-Thomas, L., & Thomas, U. (2015). Deep brain stimulation of the inferior colliculus: a possible animal model to study paradoxical kinesia observed in some parkinsonian patients? *Behavioural Brain Research*, *279*, 1–8.

Melse, M., Temel, Y., Tan, S. K., & Jahanshahi, A. (2016). Deep brain stimulation of the rostromedial tegmental nucleus: An unanticipated, selective effect on food intake. *Brain Research Bulletin*, *127*, 23–28.

Meng, D.-W., Liu, H.-G., Yang, A.-C., Zhang, K., & Zhang, J.-G. (2016). Stimulation of Anterior Thalamic Nuclei Protects Against Seizures and Neuronal Apoptosis in Hippocampal CA3 Region of Kainic Acid-induced Epileptic Rats. *Chinese Medical Journal*, *129*(8), 960–966.

Meng, H., Wang, Y., Huang, M., Lin, W., Wang, S., & Zhang, B. (2011). Chronic deep brain stimulation of the lateral habenula nucleus in a rat model of depression. *Brain Research*, *1422*, 32–38.

Mihály, I., Molnár, T., Berki, Á.-J., Bod, R.-B., Orbán-Kis, K., Gáll, Z., & Szilágyi, T. (2021). Short-Term Amygdala Low-Frequency Stimulation Does not Influence Hippocampal Interneuron Changes Observed in the Pilocarpine Model of Epilepsy. *Cells* , *10*(3). https://doi.org/10.3390/cells10030520

Mihály, I., Orbán-Kis, K., Gáll, Z., Berki, Á.-J., Bod, R.-B., & Szilágyi, T. (2020). Amygdala Low-Frequency Stimulation Reduces Pathological Phase-Amplitude Coupling in the Pilocarpine Model of Epilepsy. *Brain Sciences*, *10*(11). https://doi.org/10.3390/brainsci10110856

Miller, K. M., Patterson, J. R., Kochmanski, J., Kemp, C. J., Stoll, A. C., Onyekpe, C. U., Cole-Strauss, A., Steece-Collier, K., Howe, J. W., Luk, K. C., & Sortwell, C. E. (2021). Striatal Afferent BDNF Is Disrupted by Synucleinopathy and Partially Restored by STN DBS. *The Journal of Neuroscience: The Official Journal of the Society for Neuroscience*, *41*(9), 2039–2052.

Minbashi Moeini, M., Sadr, S. S., & Riahi, E. (2021). Deep Brain Stimulation of the Lateral Hypothalamus Facilitates Extinction and Prevents Reinstatement of Morphine Place Preference in Rats. *Neuromodulation: Journal of the International Neuromodulation Society*, *24*(2), 240–247.

Miranda, M. F., Hamani, C., de Almeida, A.-C. G., Amorim, B. O., Macedo, C. E., Fernandes, M. J. S., Nobrega, J. N., Aarão, M. C., Madureira, A. P., Rodrigues, A. M., Andersen, M. L., Tufik, S., Mello, L. E., & Covolan, L. (2014). Role of adenosine in the antiepileptic effects of deep brain stimulation. *Frontiers in Cellular Neuroscience*, *8*, 312.

Mirski, M. A., Ziai, W. C., Chiang, J., Hinich, M., & Sherman, D. (2009). Anticonvulsant serotonergic and deep brain stimulation in anterior thalamus. *Seizure: The Journal of the British Epilepsy Association*, *18*(1), 64–70.

Miterko, L. N., Lin, T., Zhou, J., van der Heijden, M. E., Beckinghausen, J., White, J. J., & Sillitoe, R. V. (2021). Neuromodulation of the cerebellum rescues movement in a mouse model of ataxia. *Nature Communications*, *12*(1), 1295.

Moers-Hornikx, V. M. P., Sesia, T., Basar, K., Lim, L. W., Hoogland, G., Steinbusch, H. W. M., Gavilanes, D. A. W. D., Temel, Y., & Vles, J. S. H. (2009). Cerebellar nuclei are involved in impulsive behaviour. *Behavioural Brain Research*, *203*(2), 256–263.

Mokhtari Hashtjini, M., Pirzad Jahromi, G., Meftahi, G. H., Esmaeili, D., & Javidnazar, D. (2018). Aqueous extract of saffron administration along with amygdala deep brain stimulation promoted alleviation of symptoms in post-traumatic stress disorder (PTSD) in rats. *Avicenna Journal of Phytomedicine*, *8*(4), 358–369.

Mundt, A., Klein, J., Joel, D., Heinz, A., Djodari-Irani, A., Harnack, D., Kupsch, A., Orawa, H., Juckel, G., Morgenstern, R., & Winter, C. (2009). High-frequency stimulation of the nucleus accumbens core and shell reduces quinpirole-induced compulsive checking in rats. *The European Journal of Neuroscience*, *29*(12), 2401–2412.

Musacchio, T., Rebenstorff, M., Fluri, F., Brotchie, J. M., Volkmann, J., Koprich, J. B., & Ip, C. W. (2017). Subthalamic nucleus deep brain stimulation is neuroprotective in the A53T α-synuclein Parkinson's

disease rat model. *Annals of Neurology*, *81*(6), 825–836.
Navailles, S., Benazzouz, A., Bioulac, B., Gross, C., & De Deurwaerdère, P. (2010). High-frequency stimulation of the subthalamic nucleus and L-3,4-dihydroxyphenylalanine inhibit in vivo serotonin release in the prefrontal cortex and hippocampus in a rat model of Parkinson's disease. *The Journal of Neuroscience: The Official Journal of the Society for Neuroscience*, *30*(6), 2356–2364.
Nikbakhtzadeh, M., Ashabi, G., Keshavarz, M., & Riahi, E. (2023). Deep brain stimulation of the lateral hypothalamus to block morphine reward: Does the intensity of stimulation matter? *Behavioural Brain Research*, *437*, 114159.
Nishida, N., Huang, Z.-L., Mikuni, N., Miura, Y., Urade, Y., & Hashimoto, N. (2007). Deep brain stimulation of the posterior hypothalamus activates the histaminergic system to exert antiepileptic effect in rat pentylenetetrazol model. *Experimental Neurology*, *205*(1), 132–144.
Noor, M. S., Murari, K., McCracken, C. B., & Kiss, Z. H. T. (2016). Spatiotemporal dynamics of cortical perfusion in response to thalamic deep brain stimulation. *NeuroImage*, *126*, 131–139.
Oterdoom, D. L. M., Lok, R., van Beek, A. P., den Dunnen, W. F. A., Emous, M., van Dijk, J. M. C., & van Dijk, G. (2020). Deep Brain Stimulation in the Nucleus Accumbens for Binge Eating Disorder: a Study in Rats. *Obesity Surgery*, *30*(10), 4145–4148.
Oza, C. S., Brocker, D. T., Behrend, C. E., & Grill, W. M. (2018). Patterned low-frequency deep brain stimulation induces motor deficits and modulates cortex-basal ganglia neural activity in healthy rats. *Journal of Neurophysiology*, *120*(5), 2410–2422.
Papp, M., Gruca, P., Faron-Górecka, A., Kusmider, M., & Willner, P. (2019). Genomic Screening of Wistar and Wistar-Kyoto Rats Exposed to Chronic Mild Stress and Deep Brain Stimulation of Prefrontal Cortex. *Neuroscience*, *423*, 66–75.
Papp, M., Gruca, P., Lason, M., Litwa, E., Solecki, W., & Willner, P. (2020). AMPA receptors mediate the pro-cognitive effects of electrical and optogenetic stimulation of the medial prefrontal cortex in antidepressant non-responsive Wistar-Kyoto rats. *Journal of Psychopharmacology* , *34*(12), 1418–1430.
Park, K., Clare, K., Volkow, N. D., Pan, Y., & Du, C. (2022). Cocaine's effects on the reactivity of the medial prefrontal cortex to ventral tegmental area stimulation: optical imaging study in mice. *Addiction* , *117*(8), 2242–2253.
Parthoens, J., Verhaeghe, J., Stroobants, S., & Staelens, S. (2014). Deep brain stimulation of the prelimbic medial prefrontal cortex: quantification of the effect on glucose metabolism in the rat brain using [(18) F]FDG microPET. *Molecular Imaging and Biology: MIB: The Official Publication of the Academy of Molecular Imaging*, *16*(6), 838–845.
Perez-Caballero, L., Pérez-Egea, R., Romero-Grimaldi, C., Puigdemont, D., Molet, J., Caso, J.-R., Mico, J.-A., Pérez, V., Leza, J.-C., & Berrocoso, E. (2014). Early responses to deep brain stimulation in depression are modulated by anti-inflammatory drugs. *Molecular Psychiatry*, *19*(5), 607–614.
Perez, S. M., Shah, A., Asher, A., & Lodge, D. J. (2013). Hippocampal deep brain stimulation reverses physiological and behavioural deficits in a rodent model of schizophrenia. *The International Journal of Neuropsychopharmacology / Official Scientific Journal of the Collegium Internationale Neuropsychopharmacologicum* , *16*(6), 1331–1339.
Pinhal, C. M., van den Boom, B. J. G., Santana-Kragelund, F., Fellinger, L., Bech, P., Hamelink, R., Feng, G., Willuhn, I., Feenstra, M. G. P., & Denys, D. (2018). Differential Effects of Deep Brain Stimulation of the Internal Capsule and the Striatum on Excessive Grooming in Sapap3 Mutant Mice. *Biological Psychiatry*, *84*(12), 917–925.
Pinheiro Campos, A. C., Martinez, R. C. R., Auada, A. V. V., Lebrun, I., Fonoff, E. T., Hamani, C., & Pagano, R. L. (2022). Effect of Subthalamic Stimulation and Electrode Implantation in the Striatal Microenvironment in a Parkinson's Disease Rat Model. *International Journal of Molecular Sciences*, *23*(20). https://doi.org/10.3390/ijms232012116
Pohodich, A. E., Yalamanchili, H., Raman, A. T., Wan, Y.-W., Gundry, M., Hao, S., Jin, H., Tang, J., Liu, Z., & Zoghbi, H. Y. (2018). Forniceal deep brain stimulation induces gene expression and splicing changes

that promote neurogenesis and plasticity. *eLife*, *7*. https://doi.org/10.7554/eLife.34031

Polar, C. A., Gupta, R., Lehmkuhle, M. J., & Dorval, A. D. (2018). Correlation between cortical beta power and gait speed is suppressed in a parkinsonian model, but restored by therapeutic deep brain stimulation. *Neurobiology of Disease*, *117*, 137–148.

Pol, S., Temel, Y., & Jahanshahi, A. (2021). A Custom Made Electrode Construct and Reliable Implantation Method That Allows for Long-Term Bilateral Deep Brain Stimulation in Mice. *Neuromodulation: Journal of the International Neuromodulation Society*, *24*(2), 212–219.

Posch, D. K., Schwabe, K., Krauss, J. K., & Lütjens, G. (2012). Deep brain stimulation of the entopeduncular nucleus in rats prevents apomorphine-induced deficient sensorimotor gating. *Behavioural Brain Research*, *232*(1), 130–136.

Praveen Rajneesh, C., Lai, C.-H., Chen, S.-C., Hsieh, T.-H., Chin, H.-Y., & Peng, C.-W. (2019). Improved voiding function by deep brain stimulation in traumatic brain-injured animals with bladder dysfunctions. *International Urology and Nephrology*, *51*(1), 41–52.

Praveen Rajneesh, C., Liou, J.-C., Hsieh, T.-H., Chin, H.-Y., & Peng, C.-W. (2020). Efficacy of Deep Brain Stimulation on the Improvement of the Bladder Functions in Traumatic Brain Injured Rats. *Brain Sciences*, *10*(11). https://doi.org/10.3390/brainsci10110850

Prinz, P., Kobelt, P., Scharner, S., Goebel-Stengel, M., Harnack, D., Faust, K., Winter, Y., Rose, M., & Stengel, A. (2017). Deep brain stimulation alters light phase food intake microstructure in rats. *Journal of Physiology and Pharmacology: An Official Journal of the Polish Physiological Society*, *68*(3), 345–354.

Qiu, C., Feng, Z., Zheng, L., & Huang, L. (2018). Frequency-Dependent Inhibition Induced by Stimulations in Rat Hippocampus. *Conference Proceedings: ... Annual International Conference of the IEEE Engineering in Medicine and Biology Society. IEEE Engineering in Medicine and Biology Society. Conference*, *2018*, 2182–2185.

Qiu, C., Feng, Z., Zheng, L., & Ma, W. (2019). Selective modulation of neuronal firing by pulse stimulations with different frequencies in rat hippocampus. *Biomedical Engineering Online*, *18*(1), 79.

Qiu, M. H., Chen, M. C., Wu, J., Nelson, D., & Lu, J. (2016). Deep brain stimulation in the globus pallidus externa promotes sleep. *Neuroscience*, *322*, 115–120.

Quinkert, A. W., & Pfaff, D. W. (2012). Temporal patterns of deep brain stimulation generated with a true random number generator and the logistic equation: effects on CNS arousal in mice. *Behavioural Brain Research*, *229*(2), 349–358.

Quinkert, A. W., Schiff, N. D., & Pfaff, D. W. (2010). Temporal patterning of pulses during deep brain stimulation affects central nervous system arousal. *Behavioural Brain Research*, *214*(2), 377–385.

Rauch, F., Schwabe, K., & Krauss, J. K. (2010). Effect of deep brain stimulation in the pedunculopontine nucleus on motor function in the rat 6-hydroxydopamine Parkinson model. *Behavioural Brain Research*, *210*(1), 46–53.

Rea, E., Rummel, J., Schmidt, T. T., Hadar, R., Heinz, A., Mathé, A. A., & Winter, C. (2014). Anti-anhedonic effect of deep brain stimulation of the prefrontal cortex and the dopaminergic reward system in a genetic rat model of depression: an intracranial self-stimulation paradigm study. *Brain Stimulation*, *7*(1), 21–28.

Reyes-Garcés, N., Diwan, M., Boyacı, E., Gómez-Ríos, G. A., Bojko, B., Nobrega, J. N., Bambico, F. R., Hamani, C., & Pawliszyn, J. (2019). In Vivo Brain Sampling Using a Microextraction Probe Reveals Metabolic Changes in Rodents after Deep Brain Stimulation. *Analytical Chemistry*, *91*(15), 9875–9884.

Reznikov, R., Bambico, F. R., Diwan, M., Raymond, R. J., Nashed, M. G., Nobrega, J. N., & Hamani, C. (2018). Prefrontal Cortex Deep Brain Stimulation Improves Fear and Anxiety-Like Behavior and Reduces Basolateral Amygdala Activity in a Preclinical Model of Posttraumatic Stress Disorder. *Neuropsychopharmacology: Official Publication of the American College of Neuropsychopharmacology*, *43*(5), 1099–1106.

Rodriguez-Romaguera, J., Do Monte, F. H. M., & Quirk, G. J. (2012). Deep brain stimulation of the ventral striatum enhances extinction of conditioned fear. *Proceedings of the National Academy of Sciences of*

the United States of America, *109*(22), 8764–8769.

Rodriguez-Romaguera, J., Do-Monte, F. H., Tanimura, Y., Quirk, G. J., & Haber, S. N. (2015). Enhancement of fear extinction with deep brain stimulation: evidence for medial orbitofrontal involvement. *Neuropsychopharmacology: Official Publication of the American College of Neuropsychopharmacology*, *40*(7), 1726–1733.

Rodriguez-Romaguera, J., Greenberg, B. D., Rasmussen, S. A., & Quirk, G. J. (2016). An Avoidance-Based Rodent Model of Exposure With Response Prevention Therapy for Obsessive-Compulsive Disorder. *Biological Psychiatry*, *80*(7), 534–540.

Roet, M., Pol, S., Schaper, F. L. W. V. J., Hoogland, G., Jahanshahi, A., & Temel, Y. (2019). Severe seizures as a side effect of deep brain stimulation in the dorsal peduncular cortex in a rat model of depression. *Epilepsy & Behavior: E&B*, *92*, 269–275.

Rogers, A. A., Aiani, L. M., Blanpain, L. T., Yuxian, S., Moore, R., & Willie, J. T. (2020). Deep brain stimulation of hypothalamus for narcolepsy-cataplexy in mice. *Brain Stimulation*, *13*(5), 1305–1316.

Ronaghi, A., Zibaii, M. I., Pandamooz, S., Nourzei, N., Motamedi, F., Ahmadiani, A., & Dargahi, L. (2019). Entorhinal cortex stimulation induces dentate gyrus neurogenesis through insulin receptor signaling. *Brain Research Bulletin*, *144*, 75–84.

Rouaud, T., Lardeux, S., Panayotis, N., Paleressompoulle, D., Cador, M., & Baunez, C. (2010). Reducing the desire for cocaine with subthalamic nucleus deep brain stimulation. *Proceedings of the National Academy of Sciences of the United States of America*, *107*(3), 1196–1200.

Rummel, J., Voget, M., Hadar, R., Ewing, S., Sohr, R., Klein, J., Sartorius, A., Heinz, A., Mathé, A. A., Vollmayr, B., & Winter, C. (2016). Testing different paradigms to optimize antidepressant deep brain stimulation in different rat models of depression. *Journal of Psychiatric Research*, *81*, 36–45.

Sahai, S., Effendi, E. T., Mahoney, E. C., Tucker, H. R., Moolick, B. J., Mamone, G., Mikkilineni, S., Gupta, M., Nicholson, A., Chua, F. Y., Akhtar, K., Hirschstein, Z., Molho, E. S., Pilitsis, J. G., & Shin, D. S. (2020). Effects of subthalamic nucleus deep brain stimulation on neuronal spiking activity in the substantia nigra pars compacta in a rat model of Parkinson's disease. *Neuroscience Letters*, *739*, 135443.

Saillet, S., Gharbi, S., Charvet, G., Deransart, C., Guillemaud, R., Depaulis, A., & David, O. (2013). Neural adaptation to responsive stimulation: a comparison of auditory and deep brain stimulation in a rat model of absence epilepsy. *Brain Stimulation*, *6*(3), 241–247.

Sani, S., Jobe, K., Smith, A., Kordower, J. H., & Bakay, R. A. E. (2007). Deep brain stimulation for treatment of obesity in rats. *Journal of Neurosurgery*, *107*(4), 809–813.

Sarica, C., Ozkan, M., Hacioglu Bay, H., Sehirli, U., Onat, F., & Ziyal, M. I. (2018). Prelimbic Cortex Deep Brain Stimulation Reduces Binge Size in a Chronic Binge Eating Rat Model. *Stereotactic and Functional Neurosurgery*, *96*(1), 33–39.

Saryyeva, A., Nakamura, M., Krauss, J. K., & Schwabe, K. (2011). c-Fos expression after deep brain stimulation of the pedunculopontine tegmental nucleus in the rat 6-hydroxydopamine Parkinson model. *Journal of Chemical Neuroanatomy*, *42*(3), 210–217.

Schippers, M. C., Bruinsma, B., Gaastra, M., Mesman, T. I., Denys, D., De Vries, T. J., & Pattij, T. (2017). Deep Brain Stimulation of the Nucleus Accumbens Core Affects Trait Impulsivity in a Baseline-Dependent Manner. *Frontiers in Behavioral Neuroscience*, *11*, 52.

Schmuckermair, C., Gaburro, S., Sah, A., Landgraf, R., Sartori, S. B., & Singewald, N. (2013). Behavioral and neurobiological effects of deep brain stimulation in a mouse model of high anxiety- and depression-like behavior. *Neuropsychopharmacology: Official Publication of the American College of Neuropsychopharmacology*, *38*(7), 1234–1244.

Schor, J. S., Gonzalez Montalvo, I., Spratt, P. W. E., Brakaj, R. J., Stansil, J. A., Twedell, E. L., Bender, K. J., & Nelson, A. B. (2022). Therapeutic deep brain stimulation disrupts movement-related subthalamic nucleus activity in parkinsonian mice. *eLife*, *11*. https://doi.org/10.7554/eLife.75253

Schor, J. S., & Nelson, A. B. (2019). Multiple stimulation parameters influence efficacy of deep brain stimulation in parkinsonian mice. *The Journal of Clinical Investigation*, *129*(9), 3833–3838.

Schumacher, A., Haegele, M., Spyth, J., & Moser, A. (2020). Electrical high frequency stimulation of the nucleus accumbens shell does not modulate depressive-like behavior in rats. *Behavioural Brain Research*, *378*, 112277.

Sesia, T., Bizup, B., & Grace, A. A. (2014). Nucleus accumbens high-frequency stimulation selectively impacts nigrostriatal dopaminergic neurons. *The International Journal of Neuropsychopharmacology / Official Scientific Journal of the Collegium Internationale Neuropsychopharmacologicum* , *17*(3), 421–427.

Sesia, T., Bulthuis, V., Tan, S., Lim, L. W., Vlamings, R., Blokland, A., Steinbusch, H. W. M., Sharp, T., Visser-Vandewalle, V., & Temel, Y. (2010). Deep brain stimulation of the nucleus accumbens shell increases impulsive behavior and tissue levels of dopamine and serotonin. *Experimental Neurology*, *225*(2), 302–309.

Shehab, S., Al-Nahdi, A., Al-Zaabi, F., Al-Mugaddam, F., Al-Sultan, M., & Ljubisavljevic, M. (2011). Effective inhibition of substantia nigra by deep brain stimulation fails to suppress tonic epileptic seizures. *Neurobiology of Disease*, *43*(3), 725–735.

Shih, Y.-Y. I., Yash, T. V., Rogers, B., & Duong, T. Q. (2014). FMRI of deep brain stimulation at the rat ventral posteromedial thalamus. *Brain Stimulation*, *7*(2), 190–193.

Shi, L.-H., Luo, F., Woodward, D., & Chang, J.-Y. (2006a). Deep brain stimulation of the substantia nigra pars reticulata exerts long lasting suppression of amygdala-kindled seizures. *Brain Research*, *1090*(1), 202–207.

Shi, L.-H., Luo, F., Woodward, D. J., & Chang, J.-Y. (2006b). Basal ganglia neural responses during behaviorally effective deep brain stimulation of the subthalamic nucleus in rats performing a treadmill locomotion test. *Synapse* , *59*(7), 445–457.

Shi, L.-H., Woodward, D. J., Luo, F., Anstrom, K., Schallert, T., & Chang, J.-Y. (2004). High-frequency stimulation of the subthalamic nucleus reverses limb-use asymmetry in rats with unilateral 6-hydroxydopamine lesions. *Brain Research*, *1013*(1), 98–106.

Silk, E., Diwan, M., Rabelo, T., Katzman, H., Campos, A. C. P., Gouveia, F. V., Giacobbe, P., Lipsman, N., & Hamani, C. (2022). Serotonin 5-HT receptors mediate the antidepressant- and anxiolytic-like effects of ventromedial prefrontal cortex deep brain stimulation in a mouse model of social defeat. *Psychopharmacology*, *239*(12), 3875–3892.

Smit, J. V., Jahanshahi, A., Janssen, M. L. F., Stokroos, R. J., & Temel, Y. (2017). Hearing assessment during deep brain stimulation of the central nucleus of the inferior colliculus and dentate cerebellar nucleus in rat. *PeerJ*, *5*, e3892.

Smit, J. V., Janssen, M. L. F., van Zwieten, G., Jahanshahi, A., Temel, Y., & Stokroos, R. J. (2016). Deep brain stimulation of the inferior colliculus in the rodent suppresses tinnitus. *Brain Research*, *1650*, 118–124.

Song, N., Du, J., Gao, Y., & Yang, S. (2020). Epitranscriptome of the ventral tegmental area in a deep brain-stimulated chronic unpredictable mild stress mouse model. *Translational Neuroscience*, *11*(1), 402–418.

So, R. Q., McConnell, G. C., August, A. T., & Grill, W. M. (2012). Characterizing effects of subthalamic nucleus deep brain stimulation on methamphetamine-induced circling behavior in hemi-Parkinsonian rats. *IEEE Transactions on Neural Systems and Rehabilitation Engineering: A Publication of the IEEE Engineering in Medicine and Biology Society*, *20*(5), 626–635.

So, R. Q., McConnell, G. C., & Grill, W. M. (2017). Frequency-dependent, transient effects of subthalamic nucleus deep brain stimulation on methamphetamine-induced circling and neuronal activity in the hemiparkinsonian rat. *Behavioural Brain Research*, *320*, 119–127.

Soto-Montenegro, M. L., Pascau, J., & Desco, M. (2014). Response to deep brain stimulation in the lateral hypothalamic area in a rat model of obesity: in vivo assessment of brain glucose metabolism. *Molecular Imaging and Biology: MIB: The Official Publication of the Academy of Molecular Imaging*, *16*(6), 830–837.

Spieles-Engemann, A. L., Behbehani, M. M., Collier, T. J., Wohlgenant, S. L., Steece-Collier, K., Paumier,

K., Daley, B. F., Gombash, S., Madhavan, L., Mandybur, G. T., Lipton, J. W., Terpstra, B. T., & Sortwell, C. E. (2010). Stimulation of the rat subthalamic nucleus is neuroprotective following significant nigral dopamine neuron loss. *Neurobiology of Disease*, *39*(1), 105–115.

Spieles-Engemann, A. L., Steece-Collier, K., Behbehani, M. M., Collier, T. J., Wohlgenant, S. L., Kemp, C. J., Cole-Strauss, A., Levine, N. D., Gombash, S. E., Thompson, V. B., Lipton, J. W., & Sortwell, C. E. (2011). Subthalamic nucleus stimulation increases brain derived neurotrophic factor in the nigrostriatal system and primary motor cortex. *Journal of Parkinson's Disease*, *1*(1), 123–136.

Spivak, Y. S., Karan, A. A., Dobryakova, Y. V., Medvedeva, T. M., Markevich, V. A., & Bolshakov, A. P. (2022). Deep Brain Stimulation of the Medial Septal Area Can Modulate Gene Expression in the Hippocampus of Rats under Urethane Anesthesia. *International Journal of Molecular Sciences*, *23*(11). https://doi.org/10.3390/ijms23116034

Sprengers, M., Raedt, R., Larsen, L. E., Delbeke, J., Wadman, W. J., Boon, P., & Vonck, K. (2020). Deep brain stimulation reduces evoked potentials with a dual time course in freely moving rats: Potential neurophysiological basis for intermittent as an alternative to continuous stimulation. *Epilepsia*, *61*(5), 903–913.

Srejic, L. R., Hamani, C., & Hutchison, W. D. (2015). High-frequency stimulation of the medial prefrontal cortex decreases cellular firing in the dorsal raphe. *The European Journal of Neuroscience*, *41*(9), 1219–1226.

Stidd, D. A., Vogelsang, K., Krahl, S. E., Langevin, J.-P., & Fellous, J.-M. (2013). Amygdala deep brain stimulation is superior to paroxetine treatment in a rat model of posttraumatic stress disorder. *Brain Stimulation*, *6*(6), 837–844.

Stone, S. S. D., Teixeira, C. M., Devito, L. M., Zaslavsky, K., Josselyn, S. A., Lozano, A. M., & Frankland, P. W. (2011). Stimulation of entorhinal cortex promotes adult neurogenesis and facilitates spatial memory. *The Journal of Neuroscience: The Official Journal of the Society for Neuroscience*, *31*(38), 13469–13484.

Sui, L., Huang, S., Peng, B., Ren, J., Tian, F., & Wang, Y. (2014). Deep brain stimulation of the amygdala alleviates fear conditioning-induced alterations in synaptic plasticity in the cortical-amygdala pathway and fear memory. *Journal of Neural Transmission* , *121*(7), 773–782.

Summerson, S. R., Aazhang, B., & Kemere, C. T. (2014). Characterizing motor and cognitive effects associated with deep brain stimulation in the GPi of hemi-Parkinsonian rats. *IEEE Transactions on Neural Systems and Rehabilitation Engineering: A Publication of the IEEE Engineering in Medicine and Biology Society*, *22*(6), 1218–1227.

Sun, Z., Jia, L., Shi, D., He, Y., Ren, Y., Yang, J., & Ma, X. (2022). Deep brain stimulation improved depressive-like behaviors and hippocampal synapse deficits by activating the BDNF/mTOR signaling pathway. *Behavioural Brain Research*, *419*, 113709.

Sutton, A. C., O'Connor, K. A., Pilitsis, J. G., & Shin, D. S. (2015). Stimulation of the subthalamic nucleus engages the cerebellum for motor function in parkinsonian rats. *Brain Structure & Function*, *220*(6), 3595–3609.

Sutton, A. C., Yu, W., Calos, M. E., Smith, A. B., Ramirez-Zamora, A., Molho, E. S., Pilitsis, J. G., Brotchie, J. M., & Shin, D. S. (2013). Deep brain stimulation of the substantia nigra pars reticulata improves forelimb akinesia in the hemiparkinsonian rat. *Journal of Neurophysiology*, *109*(2), 363–374.

Tabansky, I., Quinkert, A. W., Rahman, N., Muller, S. Z., Lofgren, J., Rudling, J., Goodman, A., Wang, Y., & Pfaff, D. W. (2014). Temporally-patterned deep brain stimulation in a mouse model of multiple traumatic brain injury. *Behavioural Brain Research*, *273*, 123–132.

Tang, W., He, X., Feng, L., Liu, D., Yang, Z., Zhang, J., Xiao, B., & Yang, Z. (2021). The Role of Hippocampal Neurogenesis in ANT-DBS for LiCl-Pilocarpine-Induced Epileptic Rats. *Stereotactic and Functional Neurosurgery*, *99*(1), 55–64.

Tan, S. K. H., Hartung, H., Schievink, S., Sharp, T., & Temel, Y. (2013). High-frequency stimulation of the substantia nigra induces serotonin-dependent depression-like behavior in animal models. *Biological Psychiatry*, *73*(2), e1–e3.

Tan, S. K. H., Hartung, H., Visser-Vandewalle, V., Steinbusch, H. W. M., Temel, Y., & Sharp, T. (2012). A combined in vivo neurochemical and electrophysiological analysis of the effect of high-frequency stimulation of the subthalamic nucleus on 5-HT transmission. *Experimental Neurology*, *233*(1), 145–153.

Tan, S. Z. K., Neoh, J., Lawrence, A. J., Wu, E. X., & Lim, L. W. (2020). Prelimbic Cortical Stimulation Improves Spatial Memory Through Distinct Patterns of Hippocampal Gene Expression in Aged Rats. *Neurotherapeutics: The Journal of the American Society for Experimental NeuroTherapeutics*, *17*(4), 2054–2068.

Temel, Y., Blokland, A., & Lim, L. W. (2012). Deactivation of the parvalbumin-positive interneurons in the hippocampus after fear-like behaviour following electrical stimulation of the dorsolateral periaqueductal gray of rats. *Behavioural Brain Research*, *233*(2), 322–325.

Temel, Y., Boothman, L. J., Blokland, A., Magill, P. J., Steinbusch, H. W. M., Visser-Vandewalle, V., & Sharp, T. (2007). Inhibition of 5-HT neuron activity and induction of depressive-like behavior by high-frequency stimulation of the subthalamic nucleus. *Proceedings of the National Academy of Sciences of the United States of America*, *104*(43), 17087–17092.

Temel, Y., Cao, C., Vlamings, R., Blokland, A., Ozen, H., Steinbusch, H. W. M., Michelsen, K. A., von Hörsten, S., Schmitz, C., & Visser-Vandewalle, V. (2006). Motor and cognitive improvement by deep brain stimulation in a transgenic rat model of Huntington's disease. *Neuroscience Letters*, *406*(1-2), 138–141.

Temel, Y., Visser-Vandewalle, V., Aendekerk, B., Rutten, B., Tan, S., Scholtissen, B., Schmitz, C., Blokland, A., & Steinbusch, H. W. M. (2005). Acute and separate modulation of motor and cognitive performance in parkinsonian rats by bilateral stimulation of the subthalamic nucleus. *Experimental Neurology*, *193*(1), 43–52.

Temel, Y., Visser-Vandewalle, V., Kaplan, S., Kozan, R., Daemen, M. A. R. C., Blokland, A., Schmitz, C., & Steinbusch, H. W. M. (2006). Protection of nigral cell death by bilateral subthalamic nucleus stimulation. *Brain Research*, *1120*(1), 100–105.

Thiele, S., Furlanetti, L., Pfeiffer, L.-M., Coenen, V. A., & Döbrössy, M. D. (2018). The effects of bilateral, continuous, and chronic Deep Brain Stimulation of the medial forebrain bundle in a rodent model of depression. *Experimental Neurology*, *303*, 153–161.

Thiele, S., Sörensen, A., Weis, J., Braun, F., Meyer, P. T., Coenen, V. A., & Döbrössy, M. D. (2020). Deep Brain Stimulation of the Medial Forebrain Bundle in a Rodent Model of Depression: Exploring Dopaminergic Mechanisms with Raclopride and Micro-PET. *Stereotactic and Functional Neurosurgery*, *98*(1), 8–20.

Torres-Sanchez, S., Perez-Caballero, L., Mico, J. A., Celada, P., & Berrocoso, E. (2018). Effect of Deep Brain Stimulation of the ventromedial prefrontal cortex on the noradrenergic system in rats. *Brain Stimulation*, *11*(1), 222–230.

Tsai, S.-T., Chen, L.-J., Wang, Y.-J., Chen, S.-Y., & Tseng, G.-F. (2016). Rostral Intralaminar Thalamic Deep Brain Stimulation Triggered Cortical and Hippocampal Structural Plasticity and Enhanced Spatial Memory. *Stereotactic and Functional Neurosurgery*, *94*(2), 108–117.

Tsai, S.-T., Chen, S.-Y., Lin, S.-Z., & Tseng, G.-F. (2020). Rostral intralaminar thalamic deep brain stimulation ameliorates memory deficits and dendritic regression in β-amyloid-infused rats. *Brain Structure & Function*, *225*(2), 751–761.

Tseng, H.-T., Hsiao, Y.-T., Yi, P.-L., & Chang, F.-C. (2020). Deep Brain Stimulation Increases Seizure Threshold by Altering REM Sleep and Delta Powers During NREM Sleep. *Front. Neurology*, *11*, 752.

Tucker, H. R., Mahoney, E., Akhtar, K., Kao, T.-J., Mamone, G., Mikkilineni, S., Ravi, M., Watkins, H., Terrelonge, D.-L., Martin, C., Unger, K., Kim, G., Fiber, K., Gupta, M., Indajang, J., Kochman, E. M., Sachs, N., Feustel, P., Molho, E. S., … Shin, D. S. (2021). Motor Thalamic Deep Brain Stimulation Alters Cortical Activity and Shows Therapeutic Utility for Treatment of Parkinson's Disease Symptoms in a Rat Model. *Neuroscience*, *460*, 88–106.

Urino, T., Hashizume, K., Maehara, M., Kato, K., Okada, Y., Hori, T., & Tanaka, T. (2010). Epileptic focus

stimulation and seizure control in the rat model of kainic acid-induced limbic seizures. *Neurologia Medico-Chirurgica*, *50*(5), 355–360.
Usui, N., Maesawa, S., Kajita, Y., Endo, O., Takebayashi, S., & Yoshida, J. (2005). Suppression of secondary generalization of limbic seizures by stimulation of subthalamic nucleus in rats. *Journal of Neurosurgery*, *102*(6), 1122–1129.
Van Den Berge, N., Albaugh, D. L., Salzwedel, A., Vanhove, C., Van Holen, R., Gao, W., Stuber, G. D., & Shih, Y.-Y. I. (2017). Functional circuit mapping of striatal output nuclei using simultaneous deep brain stimulation and fMRI. *NeuroImage*, *146*, 1050–1061.
Van Den Berge, N., Keereman, V., Vanhove, C., Van Nieuwenhuyse, B., van Mierlo, P., Raedt, R., Vonck, K., Boon, P., & Van Holen, R. (2015). Hippocampal deep brain stimulation reduces glucose utilization in the healthy rat brain. *Molecular Imaging and Biology: MIB: The Official Publication of the Academy of Molecular Imaging*, *17*(3), 373–383.
Van Den Berge, N., Vanhove, C., Descamps, B., Dauwe, I., van Mierlo, P., Vonck, K., Keereman, V., Raedt, R., Boon, P., & Van Holen, R. (2015). Functional MRI during Hippocampal Deep Brain Stimulation in the Healthy Rat Brain. *PloS One*, *10*(7), e0133245.
van der Plasse, G., Schrama, R., van Seters, S. P., Vanderschuren, L. J. M. J., & Westenberg, H. G. M. (2012). Deep brain stimulation reveals a dissociation of consummatory and motivated behaviour in the medial and lateral nucleus accumbens shell of the rat. *PloS One*, *7*(3), e33455.
van Dijk, A., Klanker, M., van Oorschot, N., Post, R., Hamelink, R., Feenstra, M. G. P., & Denys, D. (2013). Deep brain stimulation affects conditioned and unconditioned anxiety in different brain areas. *Translational Psychiatry*, *3*(7), e289.
van Dijk, A., Klompmakers, A. A., Feenstra, M. G. P., & Denys, D. (2012). Deep brain stimulation of the accumbens increases dopamine, serotonin, and noradrenaline in the prefrontal cortex. *Journal of Neurochemistry*, *123*(6), 897–903.
van Dijk, A., Mason, O., Klompmakers, A. A., Feenstra, M. G. P., & Denys, D. (2011). Unilateral deep brain stimulation in the nucleus accumbens core does not affect local monoamine release. *Journal of Neuroscience Methods*, *202*(2), 113–118.
Van Nieuwenhuyse, B., Raedt, R., Delbeke, J., Wadman, W. J., Boon, P., & Vonck, K. (2015). In search of optimal DBS paradigms to treat epilepsy: bilateral versus unilateral hippocampal stimulation in a rat model for temporal lobe epilepsy. *Brain Stimulation*, *8*(2), 192–199.
van Zwieten, G., Roberts, M. J., Schaper, F. L. V. W., Smit, J. V., Temel, Y., & Janssen, M. L. F. (2021). Noise-induced neurophysiological alterations in the rat medial geniculate body and thalamocortical desynchronization by deep brain stimulation. *Journal of Neurophysiology*, *125*(2), 661–671.
Varatharajan, R., Joseph, K., Neto, S. C., Hofmann, U. G., Moser, A., & Tronnier, V. (2015). Electrical high frequency stimulation modulates GABAergic activity in the nucleus accumbens of freely moving rats. *Neurochemistry International*, *90*, 255–260.
Vassoler, F. M., Schmidt, H. D., Gerard, M. E., Famous, K. R., Ciraulo, D. A., Kornetsky, C., Knapp, C. M., & Pierce, R. C. (2008). Deep brain stimulation of the nucleus accumbens shell attenuates cocaine priming-induced reinstatement of drug seeking in rats. *The Journal of Neuroscience: The Official Journal of the Society for Neuroscience*, *28*(35), 8735–8739.
Vassoler, F. M., White, S. L., Hopkins, T. J., Guercio, L. A., Espallergues, J., Berton, O., Schmidt, H. D., & Pierce, R. C. (2013). Deep brain stimulation of the nucleus accumbens shell attenuates cocaine reinstatement through local and antidromic activation. *The Journal of Neuroscience: The Official Journal of the Society for Neuroscience*, *33*(36), 14446–14454.
Vedam-Mai, V., Baradaran-Shoraka, M., Reynolds, B. A., & Okun, M. S. (2016). Tissue Response to Deep Brain Stimulation and Microlesion: A Comparative Study. *Neuromodulation: Journal of the International Neuromodulation Society*, *19*(5), 451–458.
Veerakumar, A., Challis, C., Gupta, P., Da, J., Upadhyay, A., Beck, S. G., & Berton, O. (2014). Antidepressant-like effects of cortical deep brain stimulation coincide with pro-neuroplastic adaptations of serotonin systems. *Biological Psychiatry*, *76*(3), 203–212.

Viana, M. B., Martins, R. S., Silva, M. S. C. F., Xapelli, S., Vaz, S. H., & Sebastião, A. M. (2021). Deep Brain Stimulation of the dorsal raphe abolishes serotonin 1A facilitation of AMPA receptor-mediated synaptic currents in the ventral hippocampus. *Behavioural Brain Research*, *403*, 113134.

Visanji, N. P., Kamali Sarvestani, I., Creed, M. C., Shams Shoaei, Z., Nobrega, J. N., Hamani, C., & Hazrati, L.-N. (2015). Deep brain stimulation of the subthalamic nucleus preferentially alters the translational profile of striatopallidal neurons in an animal model of Parkinson's disease. *Frontiers in Cellular Neuroscience*, *9*, 221.

Volle, J., Bregman, T., Scott, B., Diwan, M., Raymond, R., Fletcher, P. J., Nobrega, J. N., & Hamani, C. (2018). Deep brain stimulation and fluoxetine exert different long-term changes in the serotonergic system. *Neuropharmacology*, *135*, 63–72.

Walker, R. H., Koch, R. J., Moore, C., & Meshul, C. K. (2009). Subthalamic nucleus stimulation and lesioning have distinct state-dependent effects upon striatal dopamine metabolism. *Synapse* , *63*(2), 136–146.

Wang, H., Shi, Z., Sun, W., Zhang, J., Wang, J., Shi, Y., Yang, R., Li, C., Chen, D., Wu, J., Gongyao, G., & Xu, Y. (2020). Development of a Non-invasive Deep Brain Stimulator With Precise Positioning and Real-Time Monitoring of Bioimpedance. *Frontiers in Neuroinformatics*, *14*, 574189.

Wang, M., Jia, L., Wu, X., Sun, Z., Xu, Z., Kong, C., Ma, L., Zhao, R., & Lu, S. (2020). Deep Brain Stimulation Improves Motor Function in Rats with Spinal Cord Injury by Increasing Synaptic Plasticity. *World Neurosurgery*, *140*, e294–e303.

Wang, S., Wu, D.-C., Fan, X.-N., Zhu, M.-Z., Hu, Q.-Y., Zhou, D., Ding, M.-P., & Chen, Z. (2010). Mediodorsal thalamic stimulation is not protective against seizures induced by amygdaloid kindling in rats. *Neuroscience Letters*, *481*(2), 97–101.

Wang, X., Hu, W.-H., Zhang, K., Zhou, J.-J., Liu, D.-F., Zhang, M.-Y., & Zhang, J.-G. (2018). Acute Fornix Deep Brain Stimulation Improves Hippocampal Glucose Metabolism in Aged Mice. *Chinese Medical Journal*, *131*(5), 594–599.

Wang, Y., Shen, Y., Cai, X., Yu, J., Chen, C., Tan, B., Tan, N., Cheng, H., Fan, X., Wu, X., Liu, J., Wang, S., Wang, Y., & Chen, Z. (2021). Deep brain stimulation in the medial septum attenuates temporal lobe epilepsy via entrainment of hippocampal theta rhythm. *CNS Neuroscience & Therapeutics*, *27*(5), 577–586.

Wang, Z., Feng, Z., & Wei, X. (2018). Axonal Stimulations With a Higher Frequency Generate More Randomness in Neuronal Firing Rather Than Increase Firing Rates in Rat Hippocampus. *Frontiers in Neuroscience*, *12*, 783.

Wang, Z., Feng, Z., Yuan, Y., & Zheng, L. (2021). Suppressing synchronous firing of epileptiform activity by high-frequency stimulation of afferent fibers in rat hippocampus. *CNS Neuroscience & Therapeutics*, *27*(3), 352–362.

Whittle, N., Schmuckermair, C., Gunduz Cinar, O., Hauschild, M., Ferraguti, F., Holmes, A., & Singewald, N. (2013). Deep brain stimulation, histone deacetylase inhibitors and glutamatergic drugs rescue resistance to fear extinction in a genetic mouse model. *Neuropharmacology*, *64*(4), 414–423.

Wilden, J. A., Qing, K. Y., Hauser, S. R., McBride, W. J., Irazoqui, P. P., & Rodd, Z. A. (2014). Reduced ethanol consumption by alcohol-preferring (P) rats following pharmacological silencing and deep brain stimulation of the nucleus accumbens shell. *Journal of Neurosurgery*, *120*(4), 997–1005.

Winter, C., Bregman, T., Voget, M., Raymond, R., Hadar, R., Nobrega, J. N., & Hamani, C. (2015). Acute high frequency stimulation of the prefrontal cortex or nucleus accumbens does not increase hippocampal neurogenesis in rats. *Journal of Psychiatric Research*, *68*, 27–29.

Wscieklica, T., Silva, M. S. C. F., Lemes, J. A., Melo-Thomas, L., Céspedes, I. C., & Viana, M. B. (2017). Deep brain stimulation of the dorsal raphe inhibits avoidance and escape reactions and activates forebrain regions related to the modulation of anxiety/panic. *Behavioural Brain Research*, *321*, 193–200.

Wu, D.-C., Zhu-Ge, Z.-B., Yu, C.-Y., Fang, Q., Wang, S., Jin, C.-L., Zhang, S.-H., & Chen, Z. (2008). Low-frequency stimulation of the tuberomammillary nucleus facilitates electrical amygdaloid-kindling

acquisition in Sprague-Dawley rats. *Neurobiology of Disease*, *32*(1), 151–156.
Wu, G., Wang, L., Hong, Z., Ren, S., & Zhou, F. (2017). Hippocampal low-frequency stimulation inhibits afterdischarge and increases GABA (A) receptor expression in amygdala-kindled pharmacoresistant epileptic rats. *Neurological Research*, *39*(8), 733–743.
Wu, H.-F., Chen, Y.-J., Chu, M.-C., Hsu, Y.-T., Lu, T.-Y., Chen, I.-T., Chen, P. S., & Lin, H.-C. (2018). Deep Brain Stimulation Modified Autism-Like Deficits via the Serotonin System in a Valproic Acid-Induced Rat Model. *International Journal of Molecular Sciences*, *19*(9). https://doi.org/10.3390/ijms19092840
Wu, H., Kakusa, B., Neuner, S., Christoffel, D. J., Heifets, B. D., Malenka, R. C., & Halpern, C. H. (2022). Local accumbens in vivo imaging during deep brain stimulation reveals a strategy-dependent amelioration of hedonic feeding. *Proceedings of the National Academy of Sciences of the United States of America*, *119*(1). https://doi.org/10.1073/pnas.2109269118
Wu, L., Canna, A., Narvaez, O., Ma, J., Sang, S., Lehto, L. J., Sierra, A., Tanila, H., Zhang, Y., Gröhn, O., Low, W. C., Filip, P., Mangia, S., & Michaeli, S. (2022). Orientation selective DBS of entorhinal cortex and medial septal nucleus modulates activity of rat brain areas involved in memory and cognition. *Scientific Reports*, *12*(1), 8565.
Wu, Z., Sun, F., Li, Z., Liu, M., Tian, X., Guo, D., Wei, P., Shan, Y., Liu, T., Guo, M., Zhu, Z., Zheng, W., Wang, Y., Zhao, G., & Wang, W. (2020). Electrical stimulation of the lateral cerebellar nucleus promotes neurogenesis in rats after motor cortical ischemia. *Scientific Reports*, *10*(1), 16563.
Wyckhuys, T., Boon, P., Raedt, R., Van Nieuwenhuyse, B., Vonck, K., & Wadman, W. (2010). Suppression of hippocampal epileptic seizures in the kainate rat by Poisson distributed stimulation. *Epilepsia*, *51*(11), 2297–2304.
Wyckhuys, T., De Smedt, T., Claeys, P., Raedt, R., Waterschoot, L., Vonck, K., Van den Broecke, C., Mabilde, C., Leybaert, L., Wadman, W., & Boon, P. (2007). High frequency deep brain stimulation in the hippocampus modifies seizure characteristics in kindled rats. *Epilepsia*, *48*(8), 1543–1550.
Xia, F., Yiu, A., Stone, S. S. D., Oh, S., Lozano, A. M., Josselyn, S. A., & Frankland, P. W. (2017). Entorhinal Cortical Deep Brain Stimulation Rescues Memory Deficits in Both Young and Old Mice Genetically Engineered to Model Alzheimer's Disease. *Neuropsychopharmacology: Official Publication of the American College of Neuropsychopharmacology*, *42*(13), 2493–2503.
Xiao, G., Song, Y., Zhang, Y., Xing, Y., Xu, S., Wang, M., Wang, J., Chen, D., Chen, J., & Cai, X. (2020). Dopamine and Striatal Neuron Firing Respond to Frequency-Dependent DBS Detected by Microelectrode Arrays in the Rat Model of Parkinson's Disease. *Biosensors*, *10*(10).
Xie, J., Chen, Z., He, T., Zhu, H., Chen, T., Liu, C., Fu, X., Shen, H., & Li, T. (2022). Deep brain stimulation in the globus pallidus alleviates motor activity defects and abnormal electrical activities of the parafascicular nucleus in parkinsonian rats. *Frontiers in Aging Neuroscience*, *14*, 1020321.
Xie, J., Li, T., He, T., Xu, R., Zhang, X., Wang, X., & Geng, X. (2022). Deep brain stimulation on the external segment of the globus pallidus improves the electrical activity of internal segment of globus pallidus in a rat model of Parkinson's disease. *Brain Research*, *1797*, 148115.
Yamamoto, T., Sakakibara, R., Uchiyama, T., & Kuwabara, S. (2020). Subthalamic Stimulation Inhibits Bladder Contraction by Modulating the Local Field Potential and Catecholamine Level of the Medial Prefrontal Cortex. *Frontiers in Neuroscience*, *14*, 917.
Yamamoto, T., Uchiyama, T., Sakakibara, R., Taniguchi, J., & Kuwabara, S. (2014). The subthalamic activity and striatal monoamine are modulated by subthalamic stimulation. *Neuroscience*, *259*, 43–52.
Yang, C., Qiu, Y., Hu, X., Chen, J., Wu, Y., & Wu, X. (2020). The Effect of High-Frequency Electrical Stimulation of Bilateral Nucleus Accumbens on the Behavior of Morphine-Induced Conditioned Place Preference Rats at Extinction and Reinstatement Phases. *Evidence-Based Complementary and Alternative Medicine: eCAM*, *2020*, 8232809.
Yang, C., Zhang, J.-R., Chen, L., Ge, S.-N., Wang, J.-L., Yan, Z.-Q., Jia, D., Zhu, J.-L., & Gao, G.-D. (2015). High frequency stimulation of the STN restored the abnormal high-voltage spindles in the cortex and the globus pallidus of 6-OHDA lesioned rats. *Neuroscience Letters*, *595*, 122–127.
Yang, H., Shan, W., Fan, J., Deng, J., Luan, G., Wang, Q., Zhang, Y., & You, H. (2022). Mapping the neural

circuits responding to deep brain stimulation of the anterior nucleus of the thalamus in the rat brain. *Epilepsy Research*, *187*, 107027.

Yan, N., Chen, N., Zhu, H., Zhang, J., Sim, M., Ma, Y., & Wang, W. (2013). High-frequency stimulation of nucleus accumbens changes in dopaminergic reward circuit. *PloS One*, *8*(11), e79318.

Ye, X., Feng, Z., Wang, Z., Zheng, L., Yuan, Y., Hu, Y., & Xu, Y. (2022). Activating Interneurons in Local Inhibitory Circuits by High-Frequency Stimulations at the Efferent Fibers of Pyramidal Neurons in Rat Hippocampal CA1 Region. *Brain Sciences*, *12*(10). https://doi.org/10.3390/brainsci12101350

Young, C. K., Koke, S. J., Kiss, Z. H., & Bland, B. H. (2009). Deep brain stimulation of the posterior hypothalamic nucleus reverses akinesia in bilaterally 6-hydroxydopamine-lesioned rats. *Neuroscience*, *162*(1), 1–4.

Yu, W., Walling, I., Smith, A. B., Ramirez-Zamora, A., Pilitsis, J. G., & Shin, D. S. (2016). Deep Brain Stimulation of the Ventral Pallidum Attenuates Epileptiform Activity and Seizing Behavior in Pilocarpine-Treated Rats. *Brain Stimulation*, *9*(2), 285–295.

Yu, Y., Feng, Z., Cao, J., Guo, Z., Wang, Z., Hu, N., & Wei, X. (2016). Modulation of local field potentials by high-frequency stimulation of afferent axons in the hippocampal CA1 region. *Journal of Integrative Neuroscience*, *15*(1), 1–17.

Zepeda, N. C., Crown, L. M., Medvidovic, S., Choi, W., Sheth, M., Bergosh, M., Gifford, R., Folz, C., Lam, P., Lu, G., Featherstone, R., Liu, C. Y., Siegel, S. J., & Lee, D. J. (2022). Frequency-specific medial septal nucleus deep brain stimulation improves spatial memory in MK-801-treated male rats. *Neurobiology of Disease*, *170*, 105756.

Zhang, C., Hu, W.-H., Wu, D.-L., Zhang, K., & Zhang, J.-G. (2015). Behavioral effects of deep brain stimulation of the anterior nucleus of thalamus, entorhinal cortex and fornix in a rat model of Alzheimer's disease. *Chinese Medical Journal*, *128*(9), 1190–1195.

Zhang, C., Wei, N.-L., Wang, Y., Wang, X., Zhang, J.-G., & Zhang, K. (2015). Deep brain stimulation of the nucleus accumbens shell induces anti-obesity effects in obese rats with alteration of dopamine neurotransmission. *Neuroscience Letters*, *589*, 1–6.

Zhang, L., Meng, S., Chen, W., Chen, Y., Huang, E., Zhang, G., Liang, Y., Ding, Z., Xue, Y., Chen, Y., Shi, J., & Shi, Y. (2021). High-Frequency Deep Brain Stimulation of the Substantia Nigra Pars Reticulata Facilitates Extinction and Prevents Reinstatement of Methamphetamine-Induced Conditioned Place Preference. *Frontiers in Pharmacology*, *12*, 705813.

Zhang, Q., Wu, Z. C., Yu, J.-T., Yu, N. N., Zhong, X. L., & Tan, L. (2012). Mode-dependent effect of high-frequency electrical stimulation of the anterior thalamic nucleus on amygdala-kindled seizures in rats. *Neuroscience*, *217*, 113–122.

Zhang, Q., Wu, Z.-C., Yu, J.-T., Zhong, X.-L., Xing, Y.-Y., Tian, Y., Miao, D., & Tan, L. (2012). Anticonvulsant effect of unilateral anterior thalamic high frequency electrical stimulation on amygdala-kindled seizures in rat. *Brain Research Bulletin*, *87*(2-3), 221–226.

Zhang, S., Zhang, X., Zhong, H., Li, X., Wu, Y., Ju, J., Liu, B., Zhang, Z., Yan, H., Wang, Y., Song, K., & Hou, S.-T. (2022). Hypothermia evoked by stimulation of medial preoptic nucleus protects the brain in a mouse model of ischaemia. *Nature Communications*, *13*(1), 6890.

Zhao, M., Wang, X., Deng, J., Guan, Y., Zhou, J., Li, T., & Luan, G. (2019). Globus Pallidus Internus Electric High-Frequency Stimulation Modulates Dopaminergic Activity in the Striatum of a Rat Model of Tourette Syndrome. *World Neurosurgery*, *127*, e881–e887.

Zhou, Q., Dong, J., Xu, T., & Cai, X. (2017). Synaptic potentiation mediated by L-type voltage-dependent calcium channels mediates the antidepressive effects of lateral habenula stimulation. *Neuroscience*, *362*, 25–32.

Zhu, G., Meng, D., Chen, Y., Du, T., Liu, Y., Liu, D., Shi, L., Jiang, Y., Zhang, X., & Zhang, J. (2018). Anterior nucleus of thalamus stimulation inhibited abnormal mossy fiber sprouting in kainic acid-induced epileptic rats. *Brain Research*, *1701*, 28–35.

Ziai, W. C., Sherman, D. L., Bhardwaj, A., Zhang, N., Keyl, P. M., & Mirski, M. A. (2005). Target-specific catecholamine elevation induced by anticonvulsant thalamic deep brain stimulation. *Epilepsia*, *46*(6),

878–888.
